# Mathematical modeling of antihypertensive therapy

**DOI:** 10.3389/fphys.2022.1070115

**Published:** 2022-12-14

**Authors:** Elena Kutumova, Ilya Kiselev, Ruslan Sharipov, Galina Lifshits, Fedor Kolpakov

**Affiliations:** ^1^ Department of Computational Biology, Sirius University of Science and Technology, Sochi, Russia; ^2^ Laboratory of Bioinformatics, Federal Research Center for Information and Computational Technologies, Novosibirsk, Russia; ^3^ Biosoft.Ru, Ltd., Novosibirsk, Russia; ^4^ Specialized Educational Scientific Center, Novosibirsk State University, Novosibirsk, Russia; ^5^ Laboratory for Personalized Medicine, Center of New Medical Technologies, Institute of Chemical Biology and Fundamental Medicine SB RAS, Novosibirsk, Russia

**Keywords:** mathematical modeling, agent-based modular model, antihypertensive therapy, cardiovascular system, renal system, blood pressure regulation

## Abstract

Hypertension is a multifactorial disease arising from complex pathophysiological pathways. Individual characteristics of patients result in different responses to various classes of antihypertensive medications. Therefore, evaluating the efficacy of therapy based on *in silico* predictions is an important task. This study is a continuation of research on the modular agent-based model of the cardiovascular and renal systems (presented in the previously published article). In the current work, we included in the model equations simulating the response to antihypertensive therapies with different mechanisms of action. For this, we used the pharmacodynamic effects of the angiotensin II receptor blocker losartan, the calcium channel blocker amlodipine, the angiotensin-converting enzyme inhibitor enalapril, the direct renin inhibitor aliskiren, the thiazide diuretic hydrochlorothiazide, and the β-blocker bisoprolol. We fitted therapy parameters based on known clinical trials for all considered medications, and then tested the model’s ability to show reasonable dynamics (expected by clinical observations) after treatment with individual drugs and their dual combinations in a group of virtual patients with hypertension. The extended model paves the way for the next step in personalized medicine that is adapting the model parameters to a real patient and predicting his response to antihypertensive therapy. The model is implemented in the BioUML software and is available at https://gitlab.sirius-web.org/virtual-patient/antihypertensive-treatment-modeling.

## 1 Introduction

Hypertension is an important worldwide public-health challenge and the most common risk factor for the development of cardiovascular (myocardial infarction, stroke, coronary heart disease, heart failure), cerebrovascular (ischemic or hemorrhagic stroke, transient ischemic attack) and renal diseases (Kearney et al., 2005; Oparil et al., 2018; Campbell et al., 2014). The prevalence of hypertension in the world is 30%–45% among the adult population (Kearney et al., 2005; Irazola et al., 2016). This pathological syndrome is a complex multifactorial disease caused by genetic predisposition and complicated interplay between pathophysiological and environmental factors, from dietary characteristics to social living conditions. The maintenance of physiological blood pressure levels involves various regulators, including neural and endocrine effects, balance of fluid and electrolytes, and the action of the renin–angiotensin–aldosterone system (RAAS) (Oparil et al., 2018).

Non-drug therapy of hypertension includes lifestyle changes (weight loss, exercise, dietary modification, smoking cessation, etc.) (Tejada et al., 2006). Pharmacological therapy is used to lower blood pressure and prevent hypertension and its cardiovascular disease sequelae (Oparil et al., 2018). Currently, five major drug classes are recommended for the treatment of hypertension (Williams et al., 2018):

• angiotensin-converting enzyme inhibitors reduce RAAS activity by inhibiting the conversion of angiotensin I into angiotensin II (Dézsi, 2014);
• angiotensin receptor blockers inhibit the binding of angiotensin II to AT1-receptors in a competitive manner (Dézsi, 2014);
• β-adrenoreceptor blockers decrease cardiac output, heart rate, renin release and the sympathetic nervous system activity (Oparil et al., 2018);
• calcium channel blockers, including dihydropyridines (e.g., amlodipine), cause vasodilation (Oparil et al., 2018);
• diuretics (thiazides and thiazide-like diuretics) inhibit sodium and chloride co-transporters in renal tubules, thereby promoting natriuresis (Oparil et al., 2018).

In addition to first-line antihypertensive drugs, reserve medications, such as direct renin inhibitors, are also being considered. The advantages of direct renin inhibitors include possible greater protection from hypertensive complications, additional blood pressure reduction when used in combination therapy, a placebo-like side-effect profile, avid renal concentration, and long duration of action for some compounds (Israili et al., 2010; Bonanni and Dalla Vestra, 2012). The tactics of treatment (mono- or combination therapy, drugs with rapid absorption or with prolonged action) are selected individually for each patient.

As representatives of various classes of antihypertensive drugs in our study, we considered the angiotensin II receptor blocker losartan (Sica et al., 2005), the calcium channel blocker amlodipine (Fares et al., 2016), the angiotensin-converting enzyme inhibitor enalapril (Ferguson et al., 1982), the direct renin inhibitor aliskiren (Gradman et al., 2005), the thiazide diuretic hydrochlorothiazide (Carter et al., 2004), and the β-blocker bisoprolol (Lancaster and Sorkin, 1988).

Despite some progress in the treatment of hypertension, a number of points must be noted:

• the number of patients with uncontrolled (drug-resistant) course of the disease is growing every year (Sarafidis et al., 2013);
• the integrated contribution of multiple genetic effects to the disease pathogenesis is still unclear (Patel et al., 2017), while this contribution can reach 50% (Luft, 2001);
• the impact of changes associated with cellular aging (Fajemiroye et al., 2018) on disease progression is the subject of research;
• the choice of effective and rational combination antihypertensive therapy remains complex (Gradman et al., 2010).

To address these points, mathematical modeling of the circulatory regulation and renal function taking into account the response to antihypertensive therapies with different mechanisms of action has become increasingly important. Such modeling paves the way for solving the following problems:

• to gain mechanistic insights into the complex dynamics of circulatory regulation in response to drug treatment;
• to predict the effectiveness (or ineffectiveness) of the chosen treatment strategy before prescribing drugs to patients and thereby gain time and prevent the development of complications associated with hypertension;
• to assess the risk of disease based on the results of a genome-wide association study (International Consortium for Blood Pressure Genome-Wide Association Studies et al., 2011);
• to explore the processes of general and vascular aging *via* consideration of the age-dependent physiological parameters reproduced in the computational model (e.g., the vascular wall stiffness).

Previously created predictive mathematical models either focus on the renal/body fluid system (Uttamsingh et al., 1985; Karaaslan et al., 2005; Hallow et al., 2014; Karaaslan et al., 2014; Hallow and Gebremichael, 2017) based on the model by Guyton et al. (1972), or simulate cardiovascular and pulmonary physiology (Ottesen et al., 2004; Proshin and Solodyannikov, 2006; Paeme et al., 2011; Rosalina et al., 2019). Using this, we developed a modular agent-based model of blood pressure regulation (Kutumova et al., 2021) that describes the cardiovascular and renal systems in great detail. The main advantage of the model is that it is thoroughly calibrated. Clinical ranges cover 49 out of 132 model parameters and 69 out of 160 model variables. The remaining parameters and variables of the model either cannot be measured in the laboratory, or we could not find available data on such measurements. In addition, the range of acceptable model values is not limited to the dynamics of healthy people or patients with the same combination of cardiovascular diseases, but is sufficient to simulate different states. For example, in our baseline study, we reproduced virtual patients with such diseases as uncomplicated hypertension (Ferlinz, 1980), non-hypertensive and hypertensive diastolic heart failure (Fujimoto et al., 2008), hypertensive left ventricular (LV) hypertrophy (Melenovsky et al., 2007), and pulmonary hypertension with left heart disease (Wright et al., 2017). Detailed documentation describing all equations, parameters and variables of the model, with justification of all formulas and changes made to the primary models, is available in the supplementary file to our basic study (Kutumova et al., 2021).

The first application for this model is an extension to account for the actions of different antihypertensive agents. Note that the modular structure of the model allows this to be achieved by adding a new module responsible for calculating the pharmacodynamic functions for each agent, as well as by defining the related target points in the rest of the model. Some pharmacodynamic effects of therapy are direct. In this case, the target variable of the model is multiplied by the influence function, the value of which depends on the dose of the drug. This approach is applied in the model by Hallow et al. (2014) for a number of antihypertensive medications. Other drug effects may be indirect. For example, RAAS inhibitors cause a change in the concentration of angiotensin II, which exerts physiological actions in many target organs, including the kidneys, adrenal glands, heart, blood vessels and brain (Allen et al., 2000). During the model creation we explored in detail the targets of angiotensin II and introduced the necessary influence functions (Kutumova et al., 2021).

The second significant application is personal predictive medicine. The underlying approach, quantitative systems pharmacology modeling, involves adapting the model to physiological quantities in a particular patient and subsequently analyzing the outcomes predicted by various treatment strategies (Allen et al., 2016; Cheng et al., 2017). To study the effect of parameter variability on treatment results, quantitative systems pharmacology models are simulated using different parameterizations termed virtual patients. A virtual population, or population of “digital twins” (Bruynseels et al., 2018; Kamel Boulos and Zhang, 2021), can be generated by the following rules:

• clinical measures (blood pressure, cardiac output, blood biochemistry, heart rate, etc.) have the same values as in a real patient;
• “hidden” characteristics that were not measured in a particular patient (for example, due to the complexity or high cost of laboratory tests), but in general can be estimated from experiments (e.g., central venous pressure, peak flow rates through the heart valves, number of nephrons per kidney, etc.) or from simulation studies (e.g., sympathetic sensitivity of the systemic microvessels), vary depending on the patient’s diseases within the normal or pathological ranges.

Thus, to generate virtual hypertensive patients, we fit parameters that are either directly related to the disease (and therefore deviate from the norm), or vary within known normal ranges. Parameter fitting consists in minimizing the distances between the clinical measures and the corresponding simulated quantities. In addition, we use constrained optimization techniques to account for the set of required physiological ranges for the model variables. So the concept of thorough calibration is applied to each generated virtual patient, which is a novel approach in quantitative systems pharmacology modeling.

Another challenge that involves the creation of virtual populations is *in silico* clinical trials (Pappalardo et al., 2019). As in the case of real experiments, this task requires the specification of inclusion and exclusion criteria and assumes that clinically measurable characteristics (such as blood pressure, heart rate, etc.) have normal distributions with reasonable means and standard deviations. All *in silico* trials are equally controllable (Pappalardo et al., 2019), i.e., all drugs (or drug combinations) are tested in the same population without lifestyle changes in patients, which is not possible in real studies.

The implementation of both applications paves the way for modeling individual responses to different classes of antihypertensive agents and thus can be used for personalized predictions on a case-by-case basis. For the model development we used the BioUML software (Kolpakov et al., 2022).

## 2 Materials and methods

### 2.1 Mathematical base

We utilize the comprehensive computational model of human cardiovascular and renal systems presented by Kutumova et al. (2021) and available in the BioModels database (Malik-Sheriff et al., 2020) with ID MODEL2202160001^1^.

### 2.2 Modular architecture

A module is defined as part of a complete mathematical model with inputs and outputs to communicate with other modules:

• inputs receive variables calculated outside of the current module;
• outputs pass variables determined in the current module and required outside of it.

**FIGURE 1:**
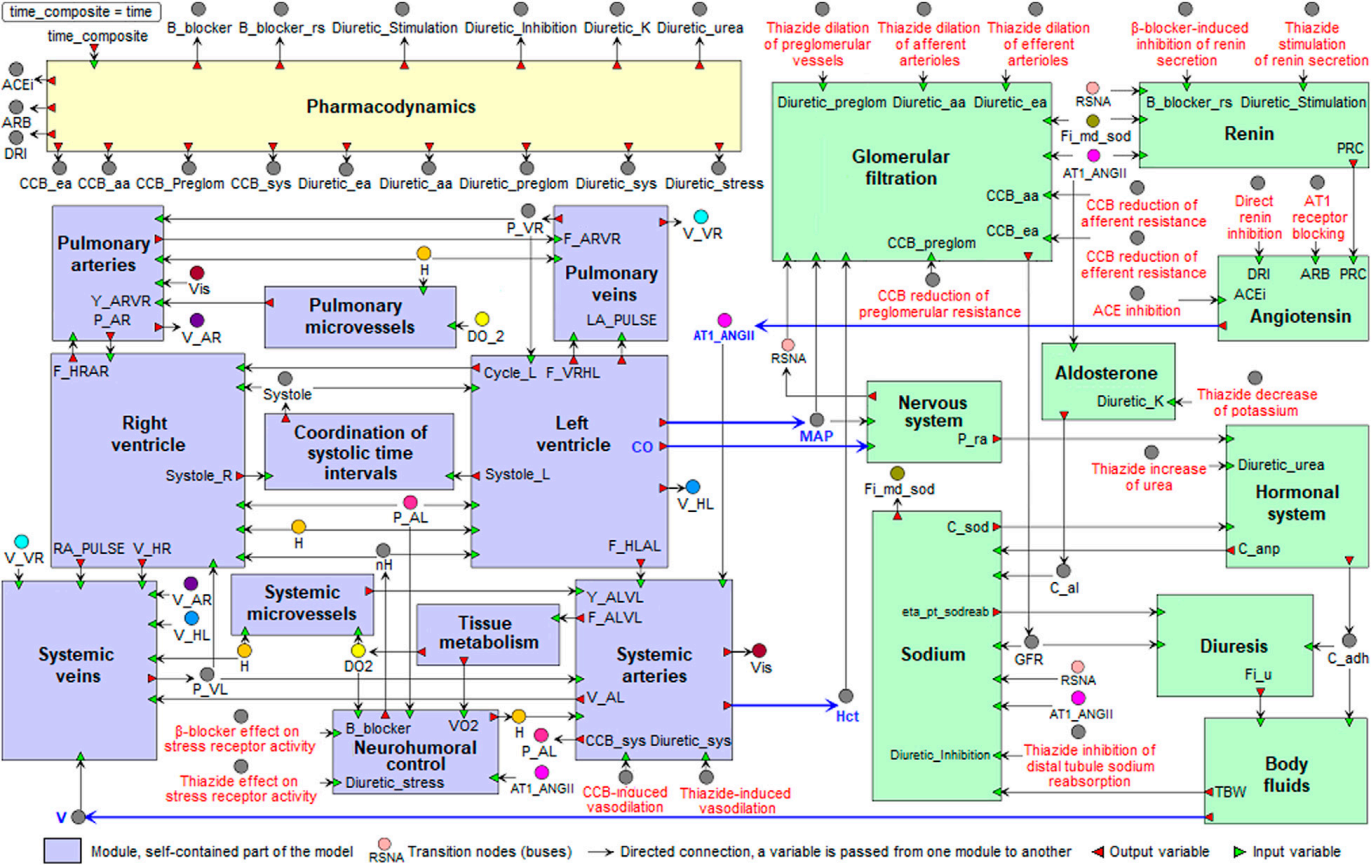
Modular agent-based model of the cardiovascular (purple) and renal (green) systems, including the pharmacodynamic module (yellow). Blue arrows indicate directed connections between the renal and cardiovascular sub-diagrams. For the visual simplicity, we added transition nodes (buses) for connections between modules. Target points of antihypertensive agents are marked in red (details are given in the section “*Modeling antihypertensive effects*” below). Abbreviations in the labels of target points: CCB, calcium channel blocker; ACE, angiotensin-converting enzyme.

The original model (Kutumova et al., 2021) has two modular representations:

• version with two modules characterizes the interaction of the cardiovascular and renal systems;
• version with 20 modules describes in more detail the integration of 11 functional modules in the cardiovascular submodel and 9 functional modules in the renal submodel (purple and green modules in Figure 1, respectively).

### 2.3 Equilibrium states

Since the model describes the dynamics of the circulatory system, its behavior is related to the cardiac cycle. In this regard, we say that the model is in an equilibrium state if all of its variable values either do not change (e.g., systolic/diastolic blood pressure) or oscillate steadily with an amplitude equal to the length of the cardiac cycle (e.g., systemic arterial pressure).

### 2.4 Agent-based simulation

The agent-based approach is used to model complex systems comprising a large number of subsystems, in particular, when it is difficult or impossible to formalize the system behavior at the global level. Within the framework of this approach, the system is considered as an aggregation of multiple parts—agents, each of which acts separately, using a set of rules, and can interact with other agents (Soheilypour and Mofrad, 2018; An et al., 2009; Macal and North, 2010). In our case, the agent is a mathematical model combined with an appropriate numerical solver. The simulation of agents consists in solving the underlying model until the next time point. The interaction between agents is handled by a separate scheduler and consists in the exchange of variable values between models.

In the case of the model of the cardiovascular and renal systems, the agent-based approach is utilized, since different submodels have vast differences in time scales: cardiovascular processes take fractions of a second, while the renal system contains long-term processes lasting minutes, which makes the whole model very stiff. Thus, we divide the model into two agents which are simulated with different solvers and exchange variable values during the simulation. To simulate each agent (i.e., to solve the Cauchy problem), we used a version of the CVODE solver (Hindmarsh et al., 2005) ported to Java and adapted to the BioUML application programming interface.

### 2.5 Frank-Starling mechanism

**FIGURE 2:**
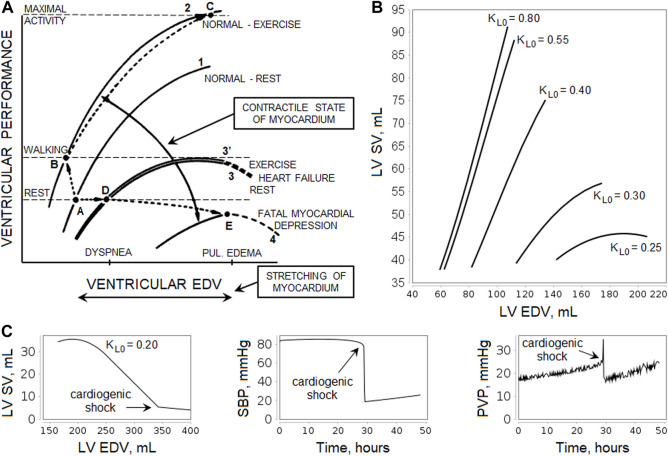
Illustration of the Frank-Starling law. **(A)** Relationship curves between ventricular performance (i.e., stroke volume, SV) and ventricular end-diastolic volume (EDV) depending on the myocardial contractility. An increase in contractility results in a shift of the curve upward and to the left (greater SV for a given level of EDV and lower EDV at any level of SV, curves 1 to 2, points A to B), while depression of contractility leads to a shift downward and to the right (curves 1 to 3, points A to D). The scheme shows the relative levels of EDV that cause dyspnea and pulmonary edema, as well as the levels of ventricular performance required during rest and walking. For more details, see the study by Braunwald et al., 1967. [Reproduced from (Braunwald et al., 1967). Copyright © 2022 Massachusetts Medical Society. Reprinted with permission from Massachusetts Medical Society]. **(B)** Simulation of the left ventricular (LV) Frank-Starling curves in the model. The curves were obtained using the normal equilibrium state by changing the oxygen demand $RO_{2}$ from 4.2 ml/s (≈250 ml/min) to 16.7 ml/s (≈1,000 ml/min) and varying the LV inotropic state $K_{L0}$ above and below the normal value of 0.55. **(C)** At a critically low value of $K_{L0}=0.2$ and a normal value of $RO_{2}=4.2$ ml/sec, the model demonstrates a reduced systolic blood pressure (SBP) and a persistent increase in pulmonary venous pressure (PVP), which after 30 h of the model experiment results in a state interpreted as cardiogenic shock. After this state, the mathematical system becomes unstable and further simulation results are not subject to analysis, since it is assumed that the virtual patient is dead.

The Frank-Starling relationship governs normal ventricular function and ensures that the volume the heart ejects in systole (stroke volume, SV) equals the volume it receives in venous return (Costanzo, 2014). That is, within physiologic limits, the heart pumps all the blood that returns to it by the way of the veins (Hall, 2011). As venous return increases, ventricular end-diastolic volume (EDV) also increases, and due to the cardiac length-tension relationship, SV increases accordingly (Figure 2A). Note that changes in heart contractility (or inotropism) affect the Frank-Starling curves (Hillegass, 2011; Costanzo, 2014). Positive inotropic agents produce an increase in SV for a given EDV and, as a result, an increase in ejection fraction (EF), calculated as

$$EF=\frac{SV}{EDV}∙100\%$$

Negative inotropic agents have the opposite effect (Costanzo, 2014). In experienced endurance athletes, the myocardial contractility is higher than in untrained healthy individuals (Schattke et al., 2014; Tanaka et al., 1986), whereas heart failure is often caused by impaired contractility (Braunwald et al., 2001). In patients with heart failure, cardiac output and external ventricular performance at rest are generally within normal limits, but an elevation of LV EDV (or preload) is associated with increases in the pulmonary capillary pressure, contributing to the dyspnea, while an elevation of right ventricular preload raises systemic venous pressure and contributes to the development of edema. LV failure becomes fatal when the SV-EDV curve is depressed to the point at which cardiac performance fails to satisfy the requirements of the body even at rest (Figure 2A).

Changes in hemodynamic parameters that occur during exercise are complex. Hyperventilation, the pumping action of the muscles, and the venoconstriction normally augment venous return. Simultaneously, the increased traffic of adrenergic nerve impulses to the myocardium, the increased concentration of circulating catecholamines, and the tachycardia augment the contractile state of the myocardium, elevating the SV without changing (or even with a reduction) of EDV (Braunwald et al., 2001). At low to moderate exercise intensity, the Frank-Starling mechanism is thought to be mainly responsible for the observed increase in SV, while submaximal and vigorous exercise involves the combination of myocardial contractility, tachycardia and the Frank-Starling mechanism (Warburton et al., 2002; Vieira et al., 2016). In heart failure, the improvement of contractility during exercise is attenuated or even prevented by norepinephrine depletion and downregulation of myocardial β-receptors, so the SV-EDV curve practically does not shift (Braunwald et al., 2001).

In our model, in accordance with the work by Proshin and Solodyannikov (2006), the maintenance of the Frank-Starling law is provided by the formula:

$$SV=K∙{SV}_{\max}∙\delta \left(EDV\right)$$

where $SV_{\max}$ denotes the theoretical maximum SV depending on the body weight, e.g., 200 ml for a 70 kg person, which is close to the SV values in top endurance athletes (Cumming, 1975; Saltin, 1969); $\delta \left(EDV\right)$ is the bell-shaped function of EDV; and *K* corresponds to the ventricular inotropic coefficient calculated from the ventricular inotropic state $K_{0}$ and the function σ , which characterizes sympathetic inotropic sensitivity and adaptive capacity of the myocardium depending on the neurohumoral factor *H*:

$$K=K_{0}+0.25∙\sigma \left(H,K_{0}\right).$$

$K_{0}$ is a constant parameter that takes values from 0.2 to 0.8. The exact form of the functions δ and σ is determined in the supplementary file of our base article (Kutumova et al., 2021), so for simplicity we don’t detail them here. Figure 2B shows an example of the relationship curves between LV SV and EDV simulated by the model. In this example, we considered the equilibrium model parametrization reproducing the physiological characteristics of a normal adult with $K_{L0}=0.55$ . To bring the model out of equilibrium and obtain Frank-Starling curves, we changed the $RO_{2}$ parameter, which is responsible for the body’s oxygen demand, from the normal value of 4.2 ml/s (≈250 ml/min) (Nathan and Singer, 1999) to 16.7 ml/s (≈1,000 ml/min), i.e., the level of moderate exercise (Thomson, 1971; Julius et al., 1967). Thus, we obtained the SV-EDV curve before the model enters a new equilibrium state at $K_{L0}=0.55$ . As this parameter was increased, the curve shifted upward and to the left, while decrease in $K_{L0}$ resulted in a shift downward and to the right. Critically low contractility values lead in the model to a persistent increase in pulmonary venous pressure, which after some model time results in a sharp drop in systolic pressure and SV (Figure 2C). We interpret this state as cardiogenic shock (Hollenberg et al., 1999; Handler, 1985), since hemodynamic criteria of this clinical condition are sustained hypotension (systolic blood pressure < 90 mmHg for at least 30 min) and a reduced cardiac index (<2.2 L/min per m^2^) in the presence of elevated pulmonary capillary occlusion pressure (>15 mmHg) (Hollenberg et al., 1999), which serves as an estimate of pulmonary venous pressure (Grignola, 2011; Chaliki et al., 2002). An interactive implementation of this example in BioUML is available online (see the Availability section).

### 2.6 Modeling antihypertensive effects

We performed drug activity modeling as follows. First, we defined target variables in the model for all antihypertensive agents, and then we multiplied each target variable by the specific function *F* that provides the pharmacodynamic effect of therapy:

$$F=1\pm \underset{D}{\sum }E\left(D\right)∙d_{F}$$ (1)

In this equation, $E\left(D\right)$ denotes the magnitude of target stimulation (the plus sign) or target inhibition (the minus sign) as a function of drug dose *D*. The summation is made for all drugs belonging to the same antihypertensive class and for all simulated dosages. The drug indicator $d_{F}$ is equal to 1 or 0 depending on whether a patient is being treated with this drug or not. All pharmacodynamic effects are initialized in a special module “Pharmacodynamics”. If only one indicator is equal to 1, then the patient receives monotherapy, if not, then combination therapy. The targets for the different antihypertensive agents incorporated in the model are shown in Figure 1 and listed in Table 1. The values of all sums $\underset{D}{\sum }E\left(D\right)∙d_{F}$ , denoted in the pharmacodynamics module as ACEi (angiotensin-converting enzyme inhibition), ARB (angiotensin receptor blocking effect), DRI (direct renin inhibition), $CCB_{ea}$ , $CCB_{aa}$ (calcium channel blocking effects), etc. (Figure 1), are passed from this module to the top level of the model and further to the modules where they are required. Below we provide a description of the pharmacodynamic functions for all targets.

**TABLE 1:** Target variables of antihypertensive drugs included in the model.^a^

Medication	Module	Target variable	Sign^b^	E(D)-value	Source of the value
Aliskiren, 150/300 mg/day	Angiotensin	Plasma renin activity	−	$\frac{0.99∙D}{D+20}$	Taken from Hallow et al. (2014).
Amlodipine, 5 mg/day	Glomerular filtration	Afferent arteriole resistance	−	0.413	Fitted to the SBP, DBP, and HR response in Porthan et al. (2009).
		Efferent arteriole resistance	−	0.107	
		Resistance of interlobar/arcuate/interlobular arteries	−	0.413	
	Systemic arteries	Resistance of the systemic microvessels	−	0.107	
Bisoprolol, 5 mg/day	Neurohumoral control	Activity of stress receptors	−	0.371	Fitted to the SBP, DBP, and HR response in Porthan et al. (2009).
	Renin	Renin secretion rate	−	0.933	
Enalapril, 20 mg/day	Angiotensin	Rate of conversion of angiotensin I to angiotensin II by angiotensin-converting enzyme	−	0.996	Fitted to the SBP, DBP, and HR response in Nedogoda et al. (2013).
HCTZ, 12.5 mg/day	Sodium	Fractional distal sodium reabsorption rate	−	0.304	Fitted to the SBP, and DBP response in MacKay et al. (1996) taking into account the following long-term dynamics: PRA increases by 45% (Villamil et al. 2007); HR, ECFV, GFR, and CO do not significantly change (van Brummelen et al. 1980, Shah et al. 1978, Scaglione et al. 1992, Scaglione et al. 1995, Duarte and Cooper- DeHoff 2010, Rapoport and Soleimani 2019, Leth 1970, Digne-Malcolm et al. 2016).
	Renin	Renin secretion rate	+	1.113	
	Glomerular filtration	Afferent arteriole resistance	−	0.469	
		Efferent arteriole resistance	−	0.302	
		Resistance of interlobar/arcuate/interlobular arteries	−	0.581	
	Systemic arteries	Resistance of the systemic microvessels	−	$\min\left(\frac{time}{1950000}∙0.074,0.074\right)$	
	Neurohumoral control	Activity of stress receptors	−	0.389	
	Aldosterone	Potassium level in the blood	−	0.030	Fitted to the serum potassium response in MacKay et al. (1996).
	Hormonal system	Urea level in the blood	+	0.100	Assumed from the blood urea response to HCTZ 25 mg in Scaglione et al. (1992).
Losartan, 50/100 mg/day	Angiotensin	Rate of angiotensin II binding to the AT1 receptors	−	0.886 (for 50 mg)	Fitted to the SBP, DBP, and HR response in Porthan et al. (2009), Nedogoda et al. (2013).
				0.954 (for 100 mg)	

^a^ SBP, systolic blood pressure; DBP, diastolic blood pressure; HR, heart rate; PRA, plasma renin activity; ECFV, extracellular fluid volume; GFR, glomerular filtration rate; CO, cardiac output; D, drug dose.

^b^ Positive or negative sign in the Eq. 1.

Aliskiren is a low molecular weight renin inhibitor of non-peptide structure (Gradman et al., 2005). The antihypertensive effect, 1–DRI , is implemented in the module “Angiotensin” by reducing plasma renin activity. The target inhibition is determined using the *E*_*max*_-model (Derendorf and Meibohm, 1999):

$$E\left(D\right)=\frac{E_{\max}∙D}{D+{ED}_{50}}$$

where $E_{\max}=0.99$ is the maximum drug effect, and $ED_{50}=20$ mg is the concentration of the medicine that gives half of the maximal effect (Hallow et al., 2014). In the model, we consider two dosages of aliskiren, 150 and 300 mg. If we denote the corresponding indicators of the drug as $Aliskiren_{150}$ and $Aliskiren_{300}$ , then the value of DRI is calculated by the formula:

$$DRI=E\left(150\right)∙{Aliskiren}_{150}+E\left(300\right)∙{Aliskiren}_{300}$$

Amlodipine causes dilation (and, as a result, a decrease in resistance) of afferent and efferent arterioles (Hayashi et al., 2007), as well as interlobar, arcuate, and interlobular arteries (Hallow et al., 2014). The corresponding influences, $1–CCB_{aa}$ , $1–CCB_{ea}$ , and $1–CCB_{preglom}$ , appear in the module “Glomerular filtration”. In addition, amlodipine acts on systemic vascular resistance (Hallow et al., 2014), which primarily depends on the conductivity of systemic microvessels (arterioles, capillaries and venules). This effect, $1–CCB_{sys}$ , is used in the module “Systemic arteries'', where it is applied to the blood flow from the arterial to the venous bed of the systemic circulation.

Bisoprolol is a β1-adrenoceptor antagonist with no partial agonist (intrinsic sympathomimetic) activity or membrane stabilizing (local anaesthetic) activity (Lancaster and Sorkin, 1988). Since β-blockers suppress renin secretion in the kidneys and reduce its level and activity (Laragh and Sealey, 2011), we added the corresponding effect, $1–B_{blocker\_rs}$ , into the module “Renin''. These drugs also block the access of catecholamines (norepinephrine, epinephrine) to their receptors so that the heart rate and blood pressure are reduced (Frishman, 2003). Therefore, we introduced the influence of $1–B_{blocker}$ on the activity of stress receptors in the module “Neurohumoral control”.

Enalapril is an angiotensin-converting enzyme inhibitor (Ferguson et al., 1982) which acts in the module “Angiotensin''. The action of the drug is modeled by multiplying the rate of conversion of angiotensin I to angiotensin II by the function 1–ACEi (Hallow et al., 2014).

Hydrochlorothiazide (HCTZ) belongs to the benzothiadiazine class, referred to simply as thiazide diuretics (Carter et al., 2004). Thiazide-induced reduction of arterial pressure includes differentiation into acute and chronic phases (Rapoport and Soleimani, 2019). The acute reduction correlates with diuresis and a decrease in plasma volume associated with inhibition of sodium reabsorption in the distal tubules (Duarte and Cooper-DeHoff, 2010). This phenomenon is described by the drug effect of $1–Diuretic_{Inhibition}$ on the normal value of fractional distal tubule sodium reabsorption (Hallow et al., 2014) in the module “Sodium”.

A possible mechanism of the chronic reduction includes vascular dilation (Rapoport and Soleimani, 2019; Duarte and Cooper-DeHoff, 2010). Thus, by analogy with the model by Hallow et al. (2014), we introduced into the module “Glomerular filtration'' the effects of $1–Diuretic_{aa}$ , $1–Diuretic_{ea}$ , and $1–Diuretic_{preglom}$ on the resistances of afferent arterioles, efferent arterioles, and interlobar/arcuate/interlobular arteries, respectively. Considering the similar effect on systemic microvessels, we took into account the following facts:

• extracellular fluid (as well as plasma volume) is initially reduced by thiazides and then almost fully recovers within 4–6 weeks of continuous treatment (Duarte and Cooper-DeHoff, 2010; Rapoport and Soleimani, 2019; Leth, 1970);
• cardiac output decreases with plasma volume loss and then returns to the baseline levels with long-term thiazide treatment (Duarte and Cooper-DeHoff, 2010; van Brummelen et al., 1980).

We found that the recovery of these values can be reproduced in the module “Systemic arteries” with a growing influence of $1/\left(1–Diuretic_{sys}\right)$ on the conductivity of systemic microvessels (the inverse of their resistance):

$${Diuretic}_{sys}\left(time\right)=\min\left(E_{\max}∙\frac{time}{duration},E_{\max}\right)$$

where $E_{\max}$ denotes the maximum influence and *duration* is the time to reach $E_{\max}$ .

As other targets of HCTZ in the model, we considered constant levels of urea and potassium in the blood. Since HCTZ increases blood urea and decreases blood potassium (Scaglione et al., 1992; Scaglione et al., 1995; Devineni et al., 2014), we multiplied these parameters by the factors $1+Diuretic_{urea}$ and $1–Diuretic_{potassium}$ (the modules “Hormonal system” and “Aldosterone”, respectively).

In addition, a clinical trial by Villamil et al. (2007) shows that HCTZ monotherapy increases plasma renin activity. Therefore, following the model by Hallow et al. (2014), we took into account the direct effect of $1+Diuretic_{Stimulation}$ on renin secretion (the module “Renin”).

Another target of the drug is based on the fact that heart rate does not change significantly with long-term treatment with HCTZ (van Brummelen et al., 1980; Shah et al., 1978; Scaglione et al., 1992; Scaglione et al., 1995). Different studies show that norepinephrine pressor response decreases or remains unchanged with thiazide diuretics (Rapoport and Soleimani, 2019). Thus, we assumed the effect of $1–Diuretic_{stress}$ on the activity of stress receptors in the module “Neurohumoral control”.

Losartan is an angiotensin-receptor antagonist without agonist properties (Sica et al., 2005). Its action is modeled in the module “Angiotensin” by the function of the form 1–ARB multiplied by the rate of angiotensin II binding to AT1 receptors (Hallow et al., 2014).

### 2.7 Virtual patient

Similar to the study by Cheng et al. (2017), we define a virtual patient as a single equilibrium parameterization of the model. This parameterization corresponds to a specific state of the patient, for example, “sick” or “healthy”. Response of the patient to external stimuli (e.g., taking antihypertensive drugs) is carried out by introducing a perturbation into the model values and subsequently solving the modified system to search for a new equilibrium. We divide the model parameters (constant values of the model) into two groups:

• the personal parameters vary within normative or pathological ranges or take a fixed value in accordance with clinical measurements in a real patient;
• the general parameters take the same values for all virtual patients.

Among the model variables, we single out observable (i.e., clinically measurable) characteristics. Such variables can be used to verify how well a virtual patient matches a real person.

### 2.8 Parameter estimation

The use of optimization methods in the current work was necessary in two problems:

• to calibrate the kinetic parameters of drugs based on experimental studies;
• to generate virtual patients with given values of physiological quantities.

In both cases, we fitted the model parameters by minimizing the distance function, defined as the normalized sum of squared differences (Hoops et al., 2006) between several observed ( $X_{i}^{\exp}$ ) and simulated equilibrium ( $X_{i}$ ) targets (blood pressure, heart rate, etc.):

$$f_{dist}\left(X_{1},\ldots ,X_{n}\right)=\overset{n}{\underset{i=1}{\sum }}\frac{\omega_{\min}}{\omega_{i}}{\left(X_{i}-X_{i}^{\exp}\right)}^{2},\;\omega_{i}=X_{i}^{\exp},\;\omega_{\min}=\min\omega_{i},\;i=1,\ldots ,n$$ (2)

Additionally, to take into account the known physiological constraints $Y_{j}^{\min}\leq Y_{j}\left(t\right)\leq Y_{j}^{\max}$ , j=1,…,m , we considered the penalty function (Runarsson and Yao, 2000):

$$f_{penalty}\left(Y_{1},\ldots ,Y_{m}\right)=\underset{t}{\sum }(\overset{m}{\underset{j=1}{\sum }}\max{\left(0,Y_{j}^{\min}-Y_{j}\left(t\right)\right)}^{2}+\overset{m}{\underset{j=1}{\sum }}\max{\left(0,Y_{j}\left(t\right)-Y_{j}^{\max}\right)}^{2})$$ (3)

To solve such optimization problems, we used a stochastic ranking evolution strategy (Runarsson and Yao, 2000) suitable for constrained global optimization.

### 2.9 Generation of one virtual patient

The task of generating a virtual patient is to find an equilibrium state of the model within the given physiological ranges. Suppose we have a medical history that includes the results of laboratory diagnostics for some person (blood pressure monitoring, electrocardiography, echocardiography, blood tests, etc.). We can directly substitute those of the measured values which are constant in the model (these are body weight and blood counts). The measured values calculated in the model (such as blood pressure, heart rate, glomerular filtration rate, etc.) can be achieved by minimizing the objective function (2). If the patient’s history confirms the presence (or absence) of any cardiovascular or renal diseases, we can also consider the constraints imposed on a number of model parameters and variables that were not explicitly measured. For example, the normal range of systemic vascular resistance is 700–1,600 dyn×s/cm^5^ (Klingensmith et al., 2016), while in patients with congestive heart failure, the values are generally higher: 944–2,209 dyn×s/cm^5^ (Laskey et al., 1990). In the case of the model parameters, such constraints determine the boundaries of the search space (the lower and upper limits of the fitting parameters). In the case of variables, they define the feasible region used to calculate the penalty function (3). A complete list of the ranges of parameters and variables that we consider in the current work is provided in Supplementary Tables S1, S2 and justified in our basic study (Kutumova et al., 2021).

**FIGURE 3:**
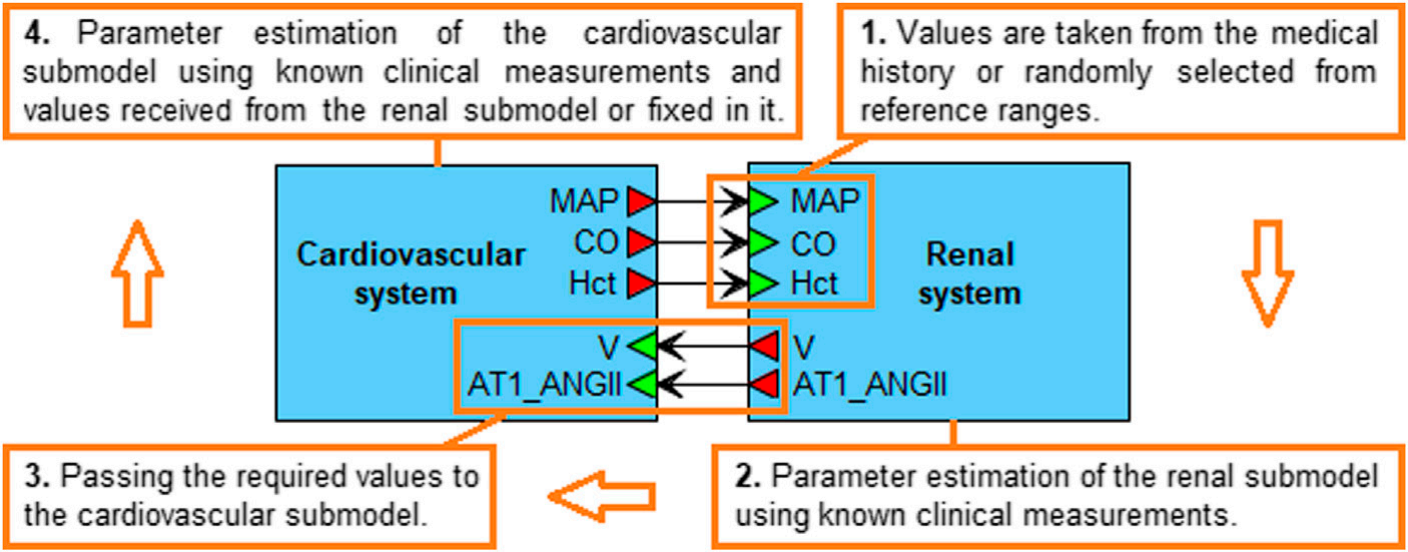
Algorithm for generating a virtual patient. The mean arterial pressure (MAP), cardiac output (CO) and hematocrit (Hct) values are taken from the real patient’s history or randomly selected from physiological ranges in accordance with the patient’s diseases. When the estimation of the renal submodel parameters is completed, the equilibrium values of the total blood volume (V) and the concentration of angiotensin II bound to the AT1 receptors (AT1_ANGII) are passed to the cardiovascular submodel. The latter is then calibrated so that the simulated MAP and CO equilibrium values, and the constant Hct value, coincide with the values set in the renal submodel. When the calibration of both submodels is finished, the equilibrium values are combined into a virtual patient.

Note that the agent-based model converges to the equilibrium state much more slowly than each of the agents independently. Therefore, the optimization of the entire model takes more time. However, the mean arterial pressure, cardiac output and hematocrit values sent by the cardiovascular submodel to the renal agent (Figure 3) are clinically measurable and can be found in the patient’s history (if not, we can randomly select them from physiological ranges). By fixing these values, we can estimate the parameters of only the renal system, pass the necessary equilibrium values to the cardiovascular agent, and then optimize its parameters by minimizing the distances between the simulated and fixed equilibriums of mean arterial pressure and cardiac output at a constant value of hematocrit. Figure 3 demonstrates the described algorithm.

### 2.10 Checking the virtual patients

In the current work, we generate virtual patients with uncomplicated hypertension. Analyzing the model, we found that some equilibrium parameterizations under perturbations of physiological parameters result in persistent increase in pulmonary venous pressure, which occurs due to the accumulation of blood in the pulmonary veins. This condition leads to pulmonary edema (Burkhoff and Tyberg, 1993; Alwi, 2010), which may be the result of a hypertensive crisis (Varounis et al., 2017). Because of the complexity of the model, the regions in the parameter space that induce such a state are not obvious and represent a separate issue for study, which is beyond the scope of this work. Here, we restrict ourselves to excluding invalid patients from the analysis. An increase in pulmonary venous blood volume can be detected with an increase in total blood volume that follows an increase in sodium intake (denoted as $\Phi_{sodin}$ in the model). Therefore, we checked each generated virtual patient by simulating the following sodium loading test. We examined the instantaneous change in $\Phi_{sodin}$ from a patient value in the range of 0.0280–0.2088 mEq/min to the elevated value of 0.243 mEq/min (He et al., 2001), and then observed the subsequent behavior of the model variables. Acceptable dynamics was characterized by convergence to a new equilibrium state with an increased systolic blood pressure (SBP) and a slightly increased pulmonary venous pressure. We considered 25 mmHg to be the maximum possible elevation of SBP based on the experimental data (He et al., 2001) reporting a mean change in this parameter of approximately 17 mmHg between low and high sodium intake in hypertensive patients.

### 2.11 Generation of the virtual population

A virtual population is a collection of unique virtual patients. To generate a population with varying values of systolic blood pressure, diastolic blood pressure (DBP), heart rate (HR), body weight, and body mass index (BMI), we used a function producing the random numbers with the required mean and standard deviation (SD).

We analyzed the baseline characteristics of hypertensive patients enrolled in different clinical trials of aliskiren, amlodipine, bisoprolol, enalapril, hydrochlorothiazide, and losartan (Abate et al., 1998; Agabiti Rosei et al., 2005; Brown et al., 2011; Corea et al., 1996; Cushman et al., 2012; Derosa et al., 2014; Fagher et al., 1990; Fermé et al., 1990; Gradman et al., 2005; Koh et al., 2004; Kwakernaak et al., 2017; Lee et al., 2012; Lithell et al., 1987; Mallion, 2007; Martina et al., 1999; Narkiewicz, 2007; Nedogoda et al., 2013; Oparil et al., 1996; Oparil et al., 2008; Pareek et al., 2016; Paterna et al., 2007; Pool et al., 2007; Porthan et al., 2009; Puig et al., 2007; Sun et al., 2016; Tham et al., 1993; Weber et al., 1995; Wing et al., 2003). Based on these characteristics, we selected suitable mean and SD values for the necessary quantities. A summary table describing the clinical trial populations is given in the Supplementary Material (Supplementary Table S3). Table 2 shows the settings of SBP, DBP, HR, BMI, weight, height, and gender that we used in our work. Note that for other parameters and variables of the model, we also took into account physiological ranges typical for a patient with uncomplicated arterial hypertension (Supplementary Tables S1, S2). Thus, as the inclusion criteria, we considered essential hypertension (SBP ≥ 140 mmHg and/or DBP ≥ 90 mmHg; SBP > 130 mmHg; DBP > 80 mmHg), BMI > 22 kg/m^2^, and average height (160–180 cm). The exclusion criteria were severe hypertension (SBP > 179.5 mmHg or DBP > 109.5 mmHg), cardiovascular or renal complications, abnormal heart rate (HR < 60 or HR > 90 beats/min), and severe obesity (BMI > 36 kg/m^2^).

**TABLE 2:** Basic settings for generating a virtual population with arterial hypertension.

Physiological characteristics	Use in the model	Basic settings (mean ± SD)	Exclusion criteria (logical operator)
SBP, mmHg	Variable	160 ± 10	(SBP < 140 and DBP < 90) or DBP > 109.5 or SBP > 179.5 or SBP < 130 or DBP < 80
DBP, mmHg	Variable	100 ± 10	
HR, beats/min	Variable	75 ± 10	HR < 60 or HR > 90
BMI, kg/m^2^	—	29 ± 5	BMI < 22 or BMI > 36
Weight, kg	Parameter	80 ± 20	—
Height, cm	Estimation of the total blood volume using the formula by Nadler et al. (1962)	—	Height < 160 or Height > 180
Sex, M/F		50%/50%	—

The algorithm for obtaining virtual patients was as follows:

1. Randomly generate SBP, DBP, HR, BMI, and weight values with the basic settings from Table 2 so that these values do not fall into the exclusion criteria.
2. Set the selected weight value into the model. Use height (calculated as $100∙\sqrt{weight/BMI}$ ), weight, and sex to estimate the search boundaries for initial total body water (line 50 in Supplementary Table S1) and the constraints for total blood volume (line 44 in Supplementary Table S2) using the formula by Nadler et al. (1962). Start generating a virtual patient by solving an optimization problem with the fitting parameters from Supplementary Table S1, the objective function (2) derived for the selected values of SBP, DBP, and HR, and the penalty function (3) constructed from the constraints in Supplementary Table S2.
3. If a solution to the optimization problem is found, check it using a sodium loading test. If the simulated dynamics of the model is appropriate, add the generated patient to the resulting population.
4. Repeat steps 1–3 until the required number of virtual patients is reached.

### 2.12 Calibration of the therapy parameters

We performed the model calibration in the following way. For the pharmacodynamics of aliskiren, we used the kinetic parameters from the study by Hallow et al. (2014). Since aliskiren does not affect HR (Stanton et al. 2003) and glomerular filtration rate (GFR) (Kwakernaak et al., 2017; Bokuda et al., 2018; Siddiqi et al., 2011), we reproduced the similar behavior of these variables in the model. For this purpose, we generated a test population of 100 virtual hypertensive patients with varying values of SBP, DBP, HR, body weight, and BMI (as described in the current section above). Then, for each patient in the population, we estimated the dimensionless parameters responsible for the effect of angiotensin II on the cardiac muscle through baroreceptors ( $sl_{baro}$ ) and stress receptors ( $sl_{stress}$ ), as well as the effect of angiotensin II on the glomerular filtration coefficient ( $sl_{KFG}$ ) (Kutumova et al., 2021). We performed all estimations so that the equilibrium values of HR and GFR before treatment with aliskiren coincided with the simulated equilibrium values after treatment. Next, we found the mean values of the estimated parameters $sl_{baro}$ , $sl_{stress}$ and $sl_{KFG}$ in the population:

$${sl}_{baro}^{mean}=0.0499,\;{sl}_{stress}^{mean}=0.2133,\;{sl}_{KFG}^{mean}=0.2015,$$

and fixed them in the model. This change led to a violation of the equilibrium states of the virtual patients. Therefore, at the next step, we found new equilibrium states. After that, we fitted the kinetic parameters of all other drugs for each patient in the adjusted population so that the simulated changes in the target physiological variables (given in the last column of Table 1) were equal to the mean changes in the corresponding experimental characteristics. As the final values of the therapy parameters [column “E(D)-value” in Table 1], we took the mean values for the population.

### 2.13 Modeling platform

BioUML (https://ict.biouml.org/; https://sirius-web.org/bioumlweb/) is a Java-based integrated environment for systems biology (Kolpakov et al., 2022). It supports a wide range of biological formats and such tools as visual modeling, simulation, parameter estimation and a number of other numerical methods. The software features used in this work are the following:

• a web-version (for collaboration and public presentation of data) and a standalone version (for independent work) of the program;
• an editor for modular and agent-based modeling;
• support of the systems biology markup language (SBML) (Hucka et al., 2019) for model exchange;
• a variety of ordinary differential equation solvers, in particular, the CVODE solver (Hindmarsh et al., 2005), ported to Java and extended to fully support SBML L3.V2;
• a variety of global optimization methods, in particular, the evolution strategy using stochastic ranking (Runarsson and Yao, 2000), which is preferably used in this article;
• integration with the Jupyter hub (https://jupyter.org/) for interactive data analysis.

## 3 Results and discussion

### 3.1 Generation of the virtual population

One of the techniques for generating virtual patients is described by Allen et al. (2016). The authors propose to create a large number (hundreds of thousands) of “plausible patients”, defined as model parameterizations within biologically plausible ranges, and then select from them a virtual population corresponding to the desired empirical distribution. To find a plausible patient, they take model inputs within the predefined plausible bounds and optimize this choice until the required outputs also fall within plausible ranges. However, the authors note that their approach is not applicable to models that are slowly simulated (for example, with dynamics across multiple time-scales, as is the case of our model) due to high computational cost to get a large plausible population. An easier way to create a virtual population is discussed in works by Hallow et al. (2014), Hallow et al. (2020), where inputs are sampled from predefined ranges and considered as valid virtual patients only if they produce outputs with physiologically reasonable values. This approach may be relevant with a relatively small number of inputs and outputs [the authors consider 11 inputs/13 outputs in one study (Hallow et al., 2014) and 14 inputs/3 outputs in another study (Hallow et al., 2020)]. However, with a large number of inputs/outputs (in our case, 57 inputs given in Supplementary Table S1 and 54 outputs given in Supplementary Table S2), it leads to enumeration of a huge number of samples with a high rejection rate. Therefore, we apply our own method for generating a virtual population. Like Allen et al., we use optimization methods with a penalty function to create virtual patients, which ensures that outputs are within physiologically acceptable ranges. In addition, for each patient, we minimize the objective function of the distances between the simulated and normally distributed random values of SBP, DBP, and HR, which directly gives us a population with predefined empirical distributions of these quantities (for more details see the *Materials and methods* section). To test the model, we generated a population of 250 virtual hypertensive patients. The baseline characteristics of this population are shown in Table 3.

**TABLE 3:** Baseline characteristics of the virtual population produced for testing the model.

Variables	All patients (*n* = 250), mean ± SD	Patients included in analysis (*n* = 186), mean ± SD
SBP, mmHg	153.36 ± 7.02	154.26 ± 7.00
DBP, mmHg	99.71 ± 6.67	101.34 ± 5.64
HR, beats/min	75.11 ± 7.37	75.81 ± 7.38
BMI, kg/m^2^	28.51 ± 3.10	28.48 ± 3.04
Weight, kg	81.62 ± 9.70	81.54 ± 9.57
Height, cm	169.20 ± 5.80	169.20 ± 5.72
Sex, M/F	139/111	100/86

### 3.2 Selection of patients with valid response to antihypertensive treatment

**FIGURE 4:**
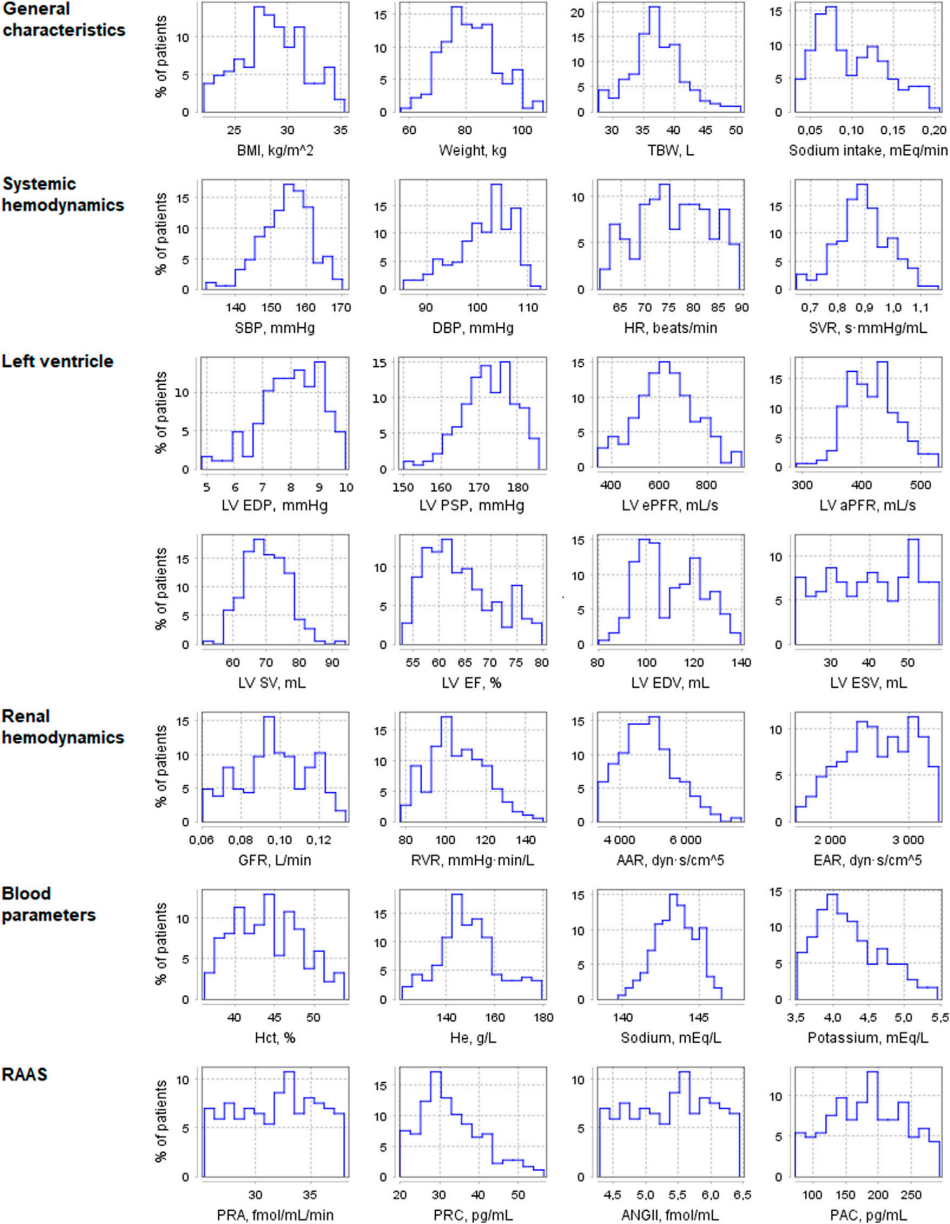
Distribution of physiological parameters in the virtual population (*n* = 186). Designations in the figure: AAR, afferent arteriolar resistance; ANGII, angiotensin II concentration; BMI, body mass index; DBP, diastolic blood pressure; EAR, efferent arteriolar resistance; GFR, glomerular filtration rate; Hct, hematocrit; He, hemoglobin; HR, heart rate; LV, left ventricular; LV aPFR, LV active peak filling rate; LV EF, LV ejection fraction; LV ePFR, LV early peak filling rate; LV EDP, LV end-diastolic pressure; LV EDV, LV end-diastolic volume; LV ESV, LV end-systolic volume; LV PSP, LV peak systolic pressure; LV SV, LV stroke volume; PAC, plasma aldosterone concentration; PRA, plasma renin activity; PRC, plasma renin concentration; RVR, renal vascular resistance; SBP, systolic blood pressure; SVR, systemic vascular resistance; TBW, total body water.

Using the pharmacodynamic models determined above, we simulated 4-week antihypertensive treatment of the generated virtual patients. To check the resulting changes in the model variables, we compiled a table describing the physiological response to therapy with aliskiren, amlodipine, bisoprolol, enalapril, HCTZ, and losartan (Supplementary Table S4). This table combines information from previously published clinical studies and includes post-treatment changes (increase, decrease, or no change) in plasma renin activity, plasma concentrations of renin, angiotensin I, angiotensin II, aldosterone, serum sodium concentration, heart rate, cardiac output, glomerular filtration rate, filtration fraction, renal blood flow, renal vascular resistance, extracellular fluid volume, and afferent arteriolar resistance. An increase (or decrease) in the long-term post-treatment dynamics of a variable was considered achieved if the corresponding change in value exceeded 5%. At the same time, if the change was less than 10%, we regarded it as insignificant. Thus, the range of changes in dynamics from 5 to 10% was taken as a transitional range due to the variability of possible changes in population variables. Virtual patients with the appropriate response (186 subjects out of 250, or 74%, “Patients included in analysis” in Table 3) were selected for further analysis. Figure 4 shows the distribution of the main parameters in this population. Distribution plots for more parameters are available in the Jupyter document on the web-version of BioUML (see the Availability section).

### 3.3 Testing the simulation of antihypertensive monotherapy

**FIGURE 5:**
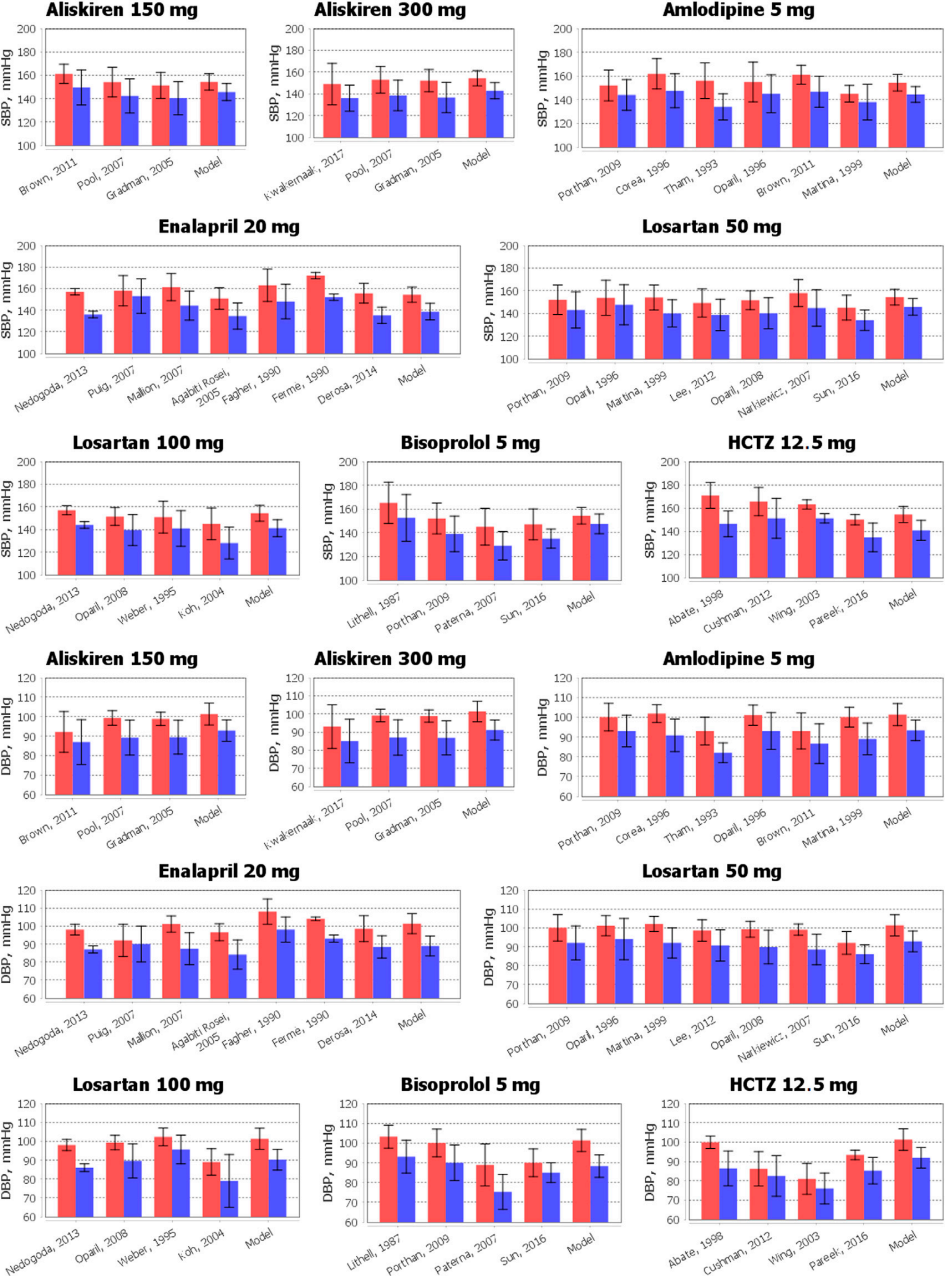
Comparison of simulated reduction in systolic blood pressure (SBP) and diastolic blood pressure (DBP) with clinical measurements obtained for aliskiren 150 or 300 mg, amlodipine 5 mg, bisoprolol 5 mg, enalapril 20 mg, HCTZ 12.5 mg, and losartan 50 or 100 mg. The data are presented as mean ± SD. The red bars denote the baseline data. The blue bars correspond to the values after therapy. To simplify the figure, we left in the references only the first author and the year of the relevant research.

The results of the comparison of the simulated changes in systolic and diastolic blood pressure with experimental measurements are shown in Figure 5. As can be seen from these data, the model accurately reproduces the reduction in blood pressure for all drugs. Numerical data related to Figure 5 and the Jupyter histogram visualization can be found in the web-version of BioUML (see the Availability section).

### 3.4 Applications of the model

In our basic study (Kutumova et al., 2021), we showed the model’s ability to simulate both healthy subjects and patients with cardiovascular pathologies, including systemic arterial hypertension, heart failure, pulmonary hypertension, etc. In the current work, we introduce the algorithm for generating virtual populations. The integration of these capabilities allows the creation of virtual populations with various combinations of cardiovascular diseases and the design of *in silico* experiments depending on the purpose of the investigation. A common problem in drug research is comparing the effects of different medicines and doses in different categories of patients. Below we provide two examples of such a comparison for mono- and combination therapies performed in the virtual population described in the previous section. All graphs given in the examples were implemented as Jupyter documents in BioUML (see the Availability section for details).

#### 3.4.1 Analysis of simulation results for antihypertensive monotherapy

**FIGURE 6:**
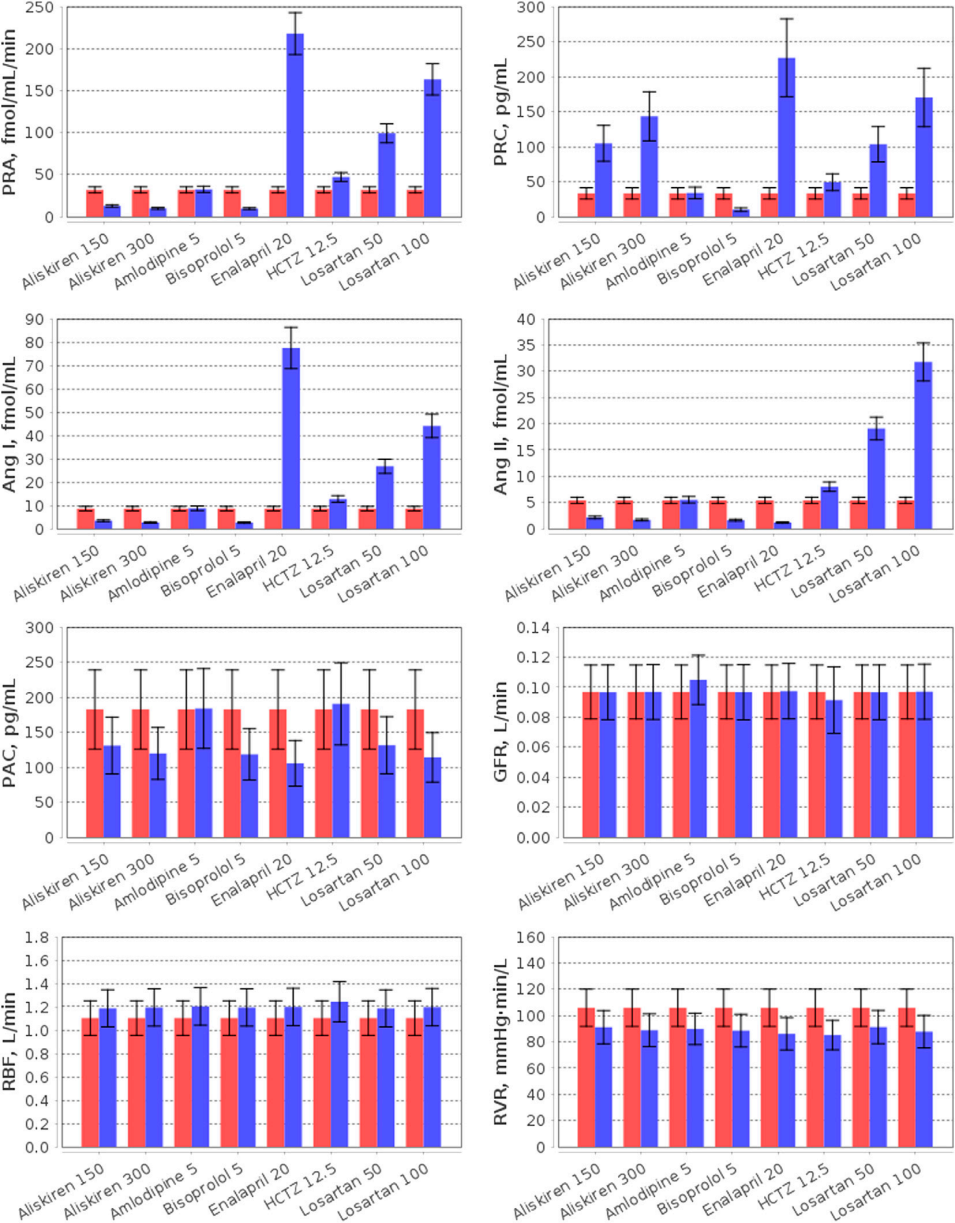
Simulation of plasma RAAS parameters (PRA, plasma renin activity; PRC, plasma renin concentration; Ang I, angiotensin I; Ang II, angiotensin II; PAC, plasma aldosterone concentration) and renal hemodynamic parameters (GFR, glomerular filtration rate; RBF, renal blood flow; RVR, renal vascular resistance) in the virtual hypertensive population at baseline (red) and after 4 weeks of treatment (blue) with aliskiren (150 or 300 mg), amlodipine (5 mg), bisoprolol (5 mg), enalapril (20 mg), HCTZ (12.5 mg), and losartan (50 or 100 mg). The data are given as mean ± SD.

**FIGURE 7:**
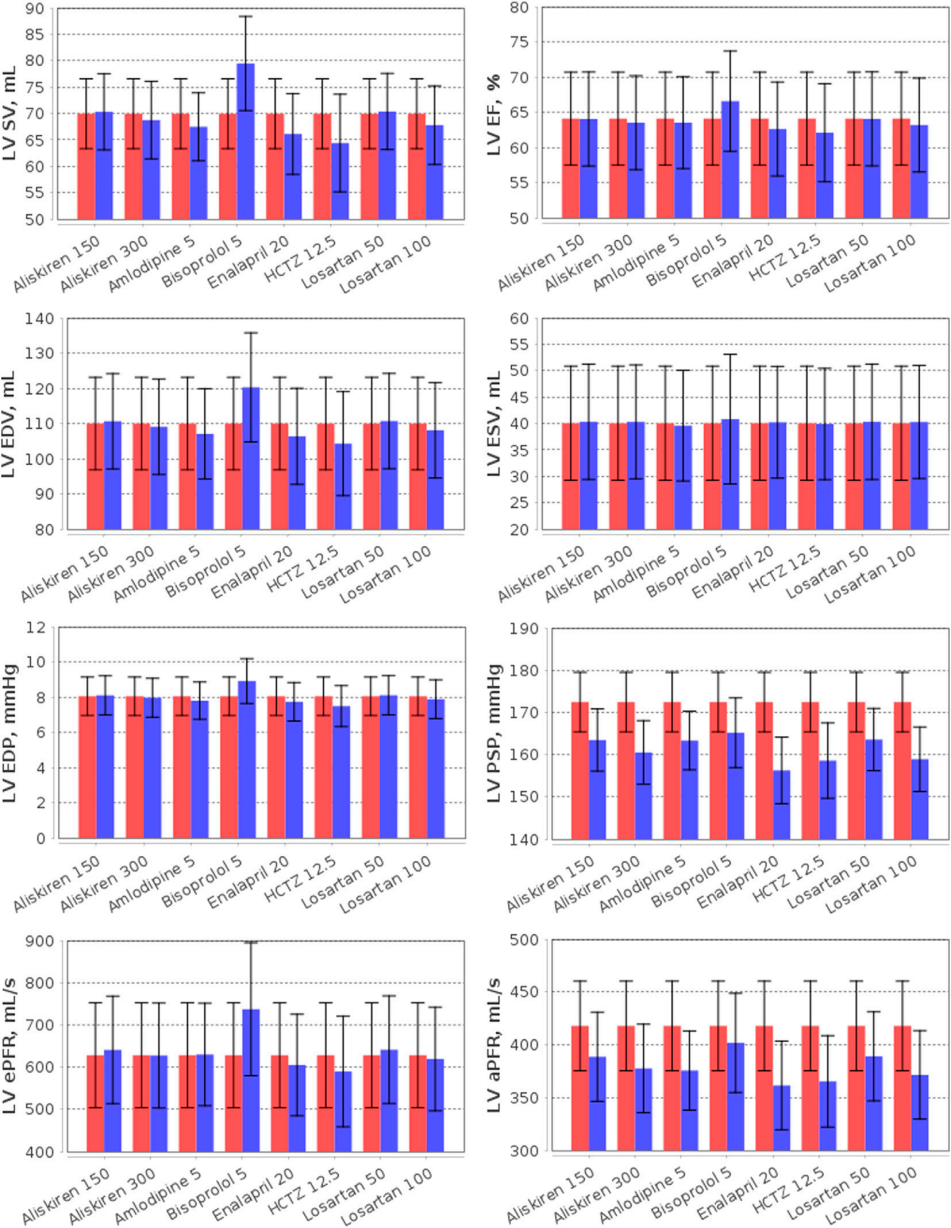
Simulation of the left ventricular (LV) parameters (LV SV, LV stroke volume; LV EF, LV ejection fraction; LV EDP, LV end-diastolic pressure; LV PSP, LV peak systolic pressure; LV EDV, LV end-diastolic volume; LV ESV, LV end-systolic volume; LV ePFR, LV early peak filling rate; LV aPFR, LV active peak filling rate) in the virtual hypertensive population at baseline (red) and after 4 weeks of treatment (blue) with aliskiren (150 or 300 mg), amlodipine (5 mg), bisoprolol (5 mg), enalapril (20 mg), HCTZ (12.5 mg), and losartan (50 or 100 mg). The data are given as mean ± SD.

Different mechanisms of action and pharmacological properties of drugs lead to different responses of physiological quantities to treatment. Generally, clinical studies evaluate the effect of antihypertensive medications on renal hemodynamics and RAAS activity. Therefore, we analysed the model-predicted change in the corresponding parameters in the population of virtual patients (*n* = 186). Figures 6, 7, as well as Supplementary Table S5, summarize the data simulated for the virtual population at baseline and after 4 weeks of treatment with aliskiren, amlodipine, bisoprolol, enalapril, HCTZ, and losartan. Below we provide a brief description of the experimental facts, which are consistent with the obtained simulation results.

The effect of RAAS inhibitors on the particular components of the system is determined by the target points of each specific drug. Aliskiren is a highly potent and selective inhibitor of human renin (Wood et al., 2003). Unlike angiotensin-converting enzyme inhibitors (such as enalapril) and angiotensin receptor blockers (such as losartan), it provides suppression of the renin system without inducing a reactive rise in plasma renin activity (PRA) (Villamil et al., 2007). Instead, aliskiren monotherapy leads to a dose-dependent decrease in PRA and an increase in plasma renin concentration (PRC) (Stanton et al., 2003; Villamil et al., 2007; Kwakernaak et al., 2017; Persson et al., 2009; Bokuda et al., 2018) with a significant reduction in the levels of angiotensin I and II (Persson et al., 2009; Cherney et al., 2012). In contrast to aliskiren, enalapril increases PRA and angiotensin I (Wiggins and Kelly, 2009), while angiotensin receptor blockers simultaneously raise PRA, PRC, angiotensin I and II (McInnes, 2007; Campbell, 2009). In our example, all RAAS inhibitors reduced plasma aldosterone concentration, as in the range of clinical studies (Bokuda et al., 2018; Nussberger et al., 2002; Fagher et al., 1990; González-Abraldes et al., 2001).

Consider the influence of other antihypertensive agents on the RAAS. The calcium antagonist amlodipine has no effect on the RAAS parameters (Licata et al., 1993; Higashi et al., 1998; Bokuda et al., 2018), whereas β-blockers (such as bisoprolol) suppress renal renin secretion (Laragh and Sealey, 2011) and reduce PRA, PRC, angiotensin I/II, and aldosterone concentrations (Savvatis, et al., 2010; Campbell, 2009; Chao et al., 2013). Thiazide diuretics (such as HCTZ) induce RAAS activation (Tamargo et al., 2014) and, as a result, increase PRA, PRC, and angiotensin I (Savvatis, et al., 2010). In addition, angiotensin II and aldosterone levels can rise (Lijnen et al., 1981; Savvatis, et al., 2010) or do not change significantly (Roman et al., 1998; Morales et al., 2015) with HCTZ.

In this example, all drugs reduced renal vascular resistance and slightly increased renal blood flow from baseline. The similar results were found in clinical trials of these medicines, where renal vascular resistance decreased, and renal blood flow raised or had no statistically significant changes (Kwakernaak et al., 2017; Licata et al., 1993; Higashi et al., 1998; Reams et al., 1987; Leeman et al., 1993; Bauer, 1984; Simon et al., 1983; Scaglione et al., 1992; Scaglione et al., 1995; van Brummelen et al., 1979; Paterna et al., 2000; Buter et al., 2001).

As shown in Figure 6, the normal value of the glomerular filtration rate did not change with aliskiren, bisoprolol, enalapril, HCTZ, and losartan, but slightly increased (within the normal range) with amlodipine, which is also consistent with the experimental data (Kwakernaak et al., 2017; Bokuda et al., 2018; Siddiqi et al., 2011; Cherney et al., 2012; Delles et al., 2003; Paterna et al., 2007; Leeman et al., 1993; Bauer, 1984; Scaglione et al., 1992; Scaglione et al., 1995; van Brummelen et al., 1979; Houlihan et al., 2002; Paterna et al., 2000; Buter et al., 2001).

Note that modelling the effect of antihypertensive drugs on renal hemodynamic parameters has already been considered in the works by (Hallow et al., 2014; Hallow and Gebremichael, 2017; Gebremichael et al., 2019; Hallow et al., 2020; Hallow et al., 2021). Therefore, our efforts in this area are not new. However, our model is an extension of the renal model by Hallow et al. (2014) and includes detailed modelling of cardiovascular hemodynamics (Proshin and Solodyannikov, 2006). In addition, we take a thorough approach to the process of generating virtual patients, imposing a large number of physiological constraints and checks on the model parameterization. All this allows us to accurately analyse the effect of antihypertensive drugs on cardiovascular parameters. For example, we consider here the effect of monotherapy on the LV parameters (Figure 7).

As can be seen from Figure 7, aliskiren did not affect LV SV, EDV and ESV, which is consistent with clinical trials (Solomon et al., 2011; Okada et al., 2017). In our case, EF also did not change in treatment with aliskiren, in accordance with a number of experimental studies (McMurray et al., 2008; Solomon et al., 2011; Pitt et al., 2011). However, Okada et al. (2017) reported that EF increased after aliskiren 150–300 mg (from 73.4 ± 5.1 to 74.7 ± 5.2 percent) in elderly hypertensive patients. Similarly, no significant changes in LV SV, EF, EDV and ESV were observed for losartan (Parrinello et al., 2009; Berezin, 2001; Yamamoto et al., 2011; Little et al., 2006), amlodipine (Picca et al., 1997; Yamamoto et al., 2011; Tham et al., 1993), and enalapril (Picca et al., 1997). Nevertheless, it should be noted that in hypertensive patients with LV hypertrophy, LV EDV and ESV may decrease significantly during long-term treatment with aliskiren 300 mg or losartan 100 mg (Solomon et al., 2009). The model predicts an increase in SV with bisoprolol, which is associated with an increase in LV EDV in accordance with the Frank-Starling law (see the *Materials and methods* section), while LV ESV does not change. Such dynamics is typical for normotensive (Palmieri et al., 2004) or hypertensive (the simulated case) (Serg et al., 2014; Suojanen et al., 2017) patients without heart disease. However, in patients with heart failure treated with bisoprolol, a decrease in LV EDV and ESV may be observed with an almost unchanged SV (which is also consistent with the Frank-Starling law, Figure 2A) (Dubach et al., 2002; Lee et al., 2016). Bisoprolol improves LV EF in patients with reduced values of the parameter (Dubach et al., 2002; Lee et al., 2016), and slightly increases (the simulated case) (van Campen et al., 2016) or does not change LV EF (Parrinello et al., 2009; Paterna et al., 2007) in patients with preserved LV EF. Figure 7 shows that HCTZ, on the contrary, reduced LV SV and EDV. The reason for this dynamic is that diuretics, by virtue of fluid loss, reduce preload and intravascular pressure, which decreases ventricular SV by the Frank-Starling mechanism (Oyama, 2015). Thus, van Brummelen et al. (1980) revealed a decrease in SV after 1 week of treatment with HCTZ 100 mg daily. According to clinical studies, HCTZ does not cause clinically significant changes in LV EF (Dey et al., 1996; Little et al., 2006).

In hypertensive patients, LV peak systolic pressure may differ significantly from SBP (e.g., Burak et al., (2019) reported 162 ± 30 vs. 135 ± 14 mmHg in patients receiving antihypertensive therapy). In the generated population of untreated hypertensives, we observed 172.4 ± 7.0 vs. 154.3 ± 7.0 mmHg for these parameters, respectively. All drugs in our test case (Figure 7) led to a decrease in LV peak systolic pressure, which is directly associated with SBP reduction during antihypertensive treatment (Stefanadis et al., 2001). At the same time, LV end-diastolic pressure had no significant changes, remaining in the normal range 3–12 mmHg (Pagani et al., 1988). Another important parameter that can be used to assess LV diastolic function is the filling rate, which is characterized by two peaks corresponding to E and A mitral inflow waves in Doppler echocardiography (Caudron et al., 2011; Zhang et al., 2019). In normal subjects, the LV inflow is greatest immediately after opening of the mitral valve (early peak velocity, E), while the left atrial contraction is responsible for smaller inflow (active peak velocity, A) (Caudron et al., 2011). In patients with arterial hypertension, the E/A ratio is lower than in healthy individuals due to a larger A peak or, in addition, a smaller E peak (Mureddu et al., 1997; de Simone et al., 2000; Tsai et al., 2012; Wu et al., 2022). The model predicts a decrease in the active peak filling rate for all drugs, whereas the early peak filling rate remains almost the same for all drugs, with the exception of bisoprolol, which increases this rate. This behaviour is consistent with the dynamics found for peak filling velocities in a number of experimental studies for amlodipine (Ogunyankin and Day, 2009), bisoprolol (de Teresa et al., 1994), HCTZ (Ogunyankin and Day, 2009; Little et al., 2006), and losartan (Little et al., 2006). However, it should be noted that other dynamics may be observed in patients with comorbidities. Thus, in patients with symptomatic heart failure, treatment with aliskiren led to a decrease in the E peak, while the A peak had no statistically significant changes (McMurray et al., 2008). In hypertensive patients with overweight and obesity, aliskiren, on the contrary, increased the E peak without changing the A peak (De Rosa et al., 2014). In elderly hypertensive patients, aliskiren did not change either the E peak or the A peak, while HCTZ decreased the E peak and did not change the A peak (Okada et al., 2017).

#### 3.4.2 Analysis of simulation results for combination antihypertensive therapy

**FIGURE 8:**
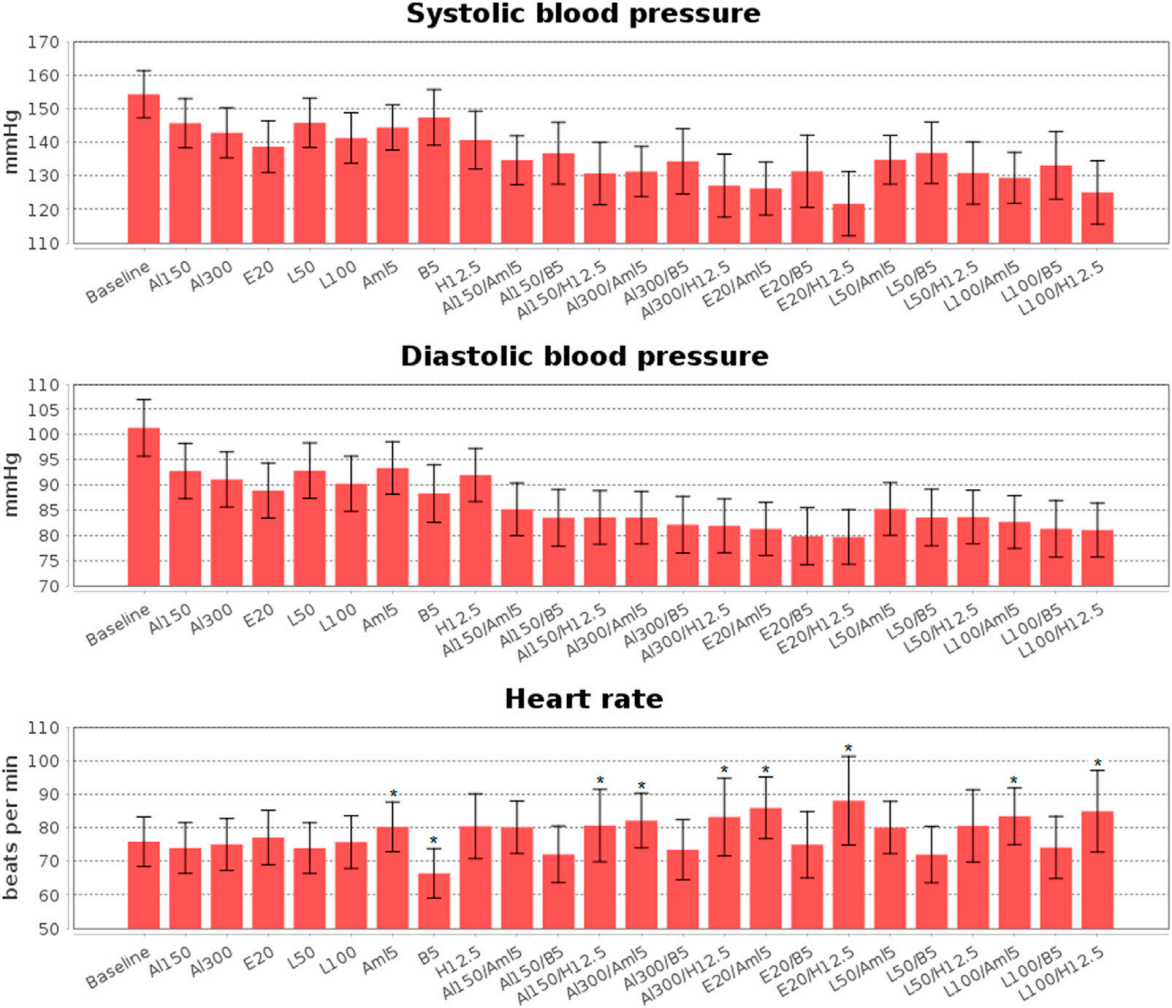
Simulated changes in systolic and diastolic blood pressure and heart rate obtained for aliskiren 150 mg (Al150), aliskiren 300 mg (Al300), enalapril 20 mg (E20), losartan 100 mg (L100), losartan 50 mg (L50), amlodipine 5 mg (Aml5), bisoprolol 5 mg (B5), hydrochlorothiazide 12.5 mg (H12.5), and combinations of one RAAS inhibitor with one drug from other antihypertensive classes. All drugs give a statistically significant (*p* < 0.0001, Kolmogorov-Smirnov test) decrease in blood pressure, while statistically significant changes in heart rate are marked with “*”. The data are presented as mean ± SD.

Calibration of the model using data for individual drugs provides the capability to estimate the response to combinations of these drugs (Hallow et al., 2014; Gebremichael et al., 2019). As an example, we considered dual combinations of RAAS blockers with non-RAAS agents. The reasonable changes in blood pressure and heart rate predicted by the model in such cases are given in Figure 8 and Table 4, the Jupyter implementation of which is provided in the web-version of BioUML (see the Availability section). As can be seen from these data, all drugs caused a statistically significant (*p* < 0.0001, Kolmogorov-Smirnov test) decrease in SBP and DBP and had different effects on HR. For example, a statistically significant decrease in HR was observed only with bisoprolol monotherapy, while the combination of this drug with RAAS blockers prevented it. In addition, there was a small but significant increase in HR (average of 4 beats per minute) with amlodipine monotherapy, which was maintained in combination with aliskiren 300 mg, enalapril 20 mg, or losartan 100 mg. HCTZ-based regimens also demonstrated a statistically significant increase in HR when co-administered with aliskiren 150–300 mg, enalapril 20 mg, or losartan 100 mg.

**TABLE 4:** Simulated changes (mean ± SD) in systolic blood pressure (SBP), diastolic blood pressure (DBP) and heart rate (HR) with estimated *p*-values (Kolmogorov-Smirnov test) for treatment effects vs*.* baseline (*P*_*base*_) and combined treatment effects vs*.* the first (*P*_*f*_) and second (*P*_*s*_) drugs in combinations.

Regimens	SBP (mmHg)	*P*_*base*_	*P*_*f*_	*P*_*s*_	DBP (mmHg)	*P*_*base*_	*P*_*f*_	*P*_*s*_	HR (beats/min)	*P*_*base*_	*P*_*f*_	*P*_*s*_
Baseline	154 ± 7	—	—	—	101 ± 6	—	—	—	76 ± 7	—	—	—
Al150	146 ± 7	SS	—	—	93 ± 6	SS	—	—	74 ± 8	0.1164	—	—
Al300	143 ± 7	SS	—	—	91 ± 5	SS	—	—	75 ± 8	0.4969	—	—
E20	139 ± 8	SS	—	—	89 ± 5	SS	—	—	77 ± 8	0.2325	—	—
L50	146 ± 7	SS	—	—	93 ± 6	SS	—	—	74 ± 8	0.1164	—	—
L100	141 ± 8	SS	—	—	90 ± 5	SS	—	—	76 ± 8	0.6679	—	—
Aml5	144 ± 7	SS	—	—	93 ± 5	SS	—	—	80 ± 7	SS	—	—
B5	147 ± 8	SS	—	—	88 ± 6	SS	—	—	66 ± 7	SS	—	—
H12.5	141 ± 9	SS	—	—	92 ± 5	SS	—	—	80 ± 10	0.0002	—	—
Al150/Aml5	135 ± 7	SS	SS	SS	85 ± 5	SS	SS	SS	80 ± 8	0.0002	SS	0.9010
Al150/B5	137 ± 9	SS	SS	SS	83 ± 6	SS	SS	SS	72 ± 8	0.0006	0.1164	SS
Al150/H12.5	131 ± 9	SS	SS	SS	84 ± 5	SS	SS	SS	81 ± 11	SS	SS	0.9010
Al300/Aml5	131 ± 7	SS	SS	SS	83 ± 5	SS	SS	SS	82 ± 8	SS	SS	0.0904
Al300/B5	134 ± 10	SS	SS	SS	82 ± 6	SS	SS	SS	73 ± 9	0.0081	0.0904	SS
Al300/H12.5	127 ± 9	SS	SS	SS	82 ± 5	SS	SS	SS	83 ± 12	SS	SS	0.0295
E20/Aml5	126 ± 8	SS	SS	SS	81 ± 5	SS	SS	SS	86 ± 9	SS	SS	SS
E20/B5	131 ± 11	SS	SS	SS	80 ± 6	SS	SS	SS	75 ± 10	0.0695	0.0397	SS
E20/H12.5	122 ± 10	SS	SS	SS	80 ± 5	SS	SS	SS	88 ± 13	SS	SS	SS
L50/Aml5	135 ± 7	SS	SS	SS	85 ± 5	SS	SS	SS	80 ± 8	0.0002	SS	0.9010
L50/B5	137 ± 9	SS	SS	SS	84 ± 6	SS	SS	SS	72 ± 8	0.0004	0.1164	SS
L50/H12.5	131 ± 9	SS	SS	SS	84 ± 5	SS	SS	SS	81 ± 11	0.0002	SS	0.9010
L100/Aml5	129 ± 8	SS	SS	SS	83 ± 5	SS	SS	SS	83 ± 8	SS	SS	0.0057
L100/B5	133 ± 10	SS	SS	SS	81 ± 6	SS	SS	SS	74 ± 9	0.0114	0.1164	SS
L100/H12.5	125 ± 9	SS	SS	SS	81 ± 5	SS	SS	SS	85 ± 12	SS	SS	0.0006

Al150, aliskiren 150 mg; Al300, aliskiren 300 mg; E20, enalapril 20 mg; L100, losartan 100 mg; L50, losartan 50 mg; Aml5, amlodipine 5 mg; B5, bisoprolol 5 mg; H12.5, hydrochlorothiazide 12.5 mg; SS, statistically significant (*p* < 0.0001).

Simulation results of 4-week treatment with aliskiren (150 and 300 mg), losartan (50 and 100 mg), enalapril (20 mg), and their dual combinations with amlodipine (5 mg), bisoprolol (5 mg), and HCTZ (12.5 mg) are given in Supplementary Tables S6–S8, respectively. In addition, Supplementary Tables S9, S10 include Pearson correlation coefficients between physiological parameters of the population and simulated reduction in SBP and DBP. We list several conclusions that can be drawn from these correlation tables.

Body mass index: RAAS agents, as well as their combinations with bisoprolol and amlodipine, demonstrate a weak negative correlation between a decrease in SBP/DBP and BMI. The correlation coefficient for DBP is in most cases lower in absolute value. In addition, with mono- or combination therapy with HCTZ, such a correlation disappears. Therefore, the model confirms that diuretic-based regimens seem to be a reasonable choice in obese patients (Weber et al., 2013). Note that the meta-analysis by Zhang et al. (2014) revealed a similar dependency for SBP: among three groups of patients taking antihypertensive therapy with the same baseline SBP, an overweight group showed a greater reduction in SBP than an obesity group, but a smaller reduction than a normal-weight group. However, both the overweight group and the obesity group had a larger DBP reduction than the normal group, while no significant difference was found between the overweight and obesity groups.

Sodium intake: HCTZ-based regimens give a weak positive correlation of SBP/DBP reduction with sodium intake. This result finds experimental confirmation. Thus, van Brummelen et al. (1978) found that sodium restriction led to a smaller decrease in blood pressure during HCTZ than a normal sodium diet. Similar results were obtained for the combination of losartan and HCTZ at low and high sodium intake (Vogt et al., 2008). These findings, in line with van Brummelen’s study (1978), call into question the therapeutic value of a low-sodium diet in hypertensive patients receiving thiazide diuretics.

Baseline SBP and DBP: The model predicts correlation between initial blood pressure and response to antihypertensive treatment. For example, the reduction in SBP and DBP after simulated treatment with amlodipine was positively correlated with baseline SBP and DBP, respectively. The same relationship was clinically observed for both amlodipine (Kario and Shimada, 1997) and another calcium antagonist, nifedipine (Hu et al., 2017). A number of experimental studies showed a similar trend towards a greater decrease in SBP and DBP among patients with higher baseline SBP and DBP levels when using other antihypertensive regimens, including therapy with losartan (Naritomi et al., 2008), enalapril or sacubitril/valsartan (Böhm et al., 2017), amlodipine/losartan (Unniachan et al., 2014), and aliskiren or aliskiren/HCTZ (Black et al., 2010). On the other hand, Zheng et al. (2011) found a negative correlation of DBP reduction with baseline DBP (while there was no correlation for SBP) in both candesartan and losartan treatment groups. In our case, we observed positive correlation coefficients in the range of 0.14–0.60 for DBP and all drug regimens, excluding bisoprolol monotherapy (with an insignificant coefficient of 0.05). At the same time, the corresponding coefficients for SBP were insignificant (less than 0.1 in absolute value) for almost all treatment schemes, whereas a negative correlation was found for bisoprolol.

Systemic arterial tone: Sustained increases in arterial tone are an essential component in the development of hypertension (Amberg et al., 2003). All drugs in our example demonstrated positive correlations of this parameter with the fall in SBP.

Systemic arterial elasticity: Strictly speaking, the term “Elasticity” is analogous to stiffness. The measures of arterial elasticity used regularly (i.e., incremental Young’s modulus, Peterson’s modulus, pulse wave velocity, and characteristic impedance) all increase with rise in stiffness and decrease with its fall. Arterial stiffening is the principal cause of increasing systolic pressure with advancing years and in patients with arterial hypertension (O’Rourke, 1990). The model showed positive correlations of arterial elasticity with SBP reduction for amlodipine- and enalapril-based regimens. This result is directly related to the fact that vasodilator agents (such as calcium antagonists and angiotensin converting enzyme inhibitors) can dilate medium-sized (brachial and carotid) arteries and at the same time reduce their stiffness (O’Rourke, 1990; Dudenbostel and Glasser, 2012; Nedogoda et al., 2017).

LV filling and ejection: As can be seen from Supplementary Table S9, RAAS agents and their combinations with bisoprolol demonstrated a negative correlation between LV active peak filling rate and SBP response. The addition of amlodipine or HCTZ to therapy reduced the absolute values of the correlation coefficients in this case. In addition, amlodipine-based regimens had a positive correlation between LV peak ejection rate (aortic valve peak flow) and decrease in SBP. We could not find confirmation of these facts in the literature. Thus, this prediction needs to be verified experimentally.

Renal hemodynamics: The response to treatment for all antihypertensive drugs showed a significant dependence on the parameters of the renal system. In particular, baseline GFR is positively correlated with reductions in SBP and DBP (with Pearson coefficients in the range of 0.19–0.47) for all regimens without HCTZ, for which correlation coefficients are negligible. The similar results were observed by Black et al. (2010) in the clinical trial of aliskiren and aliskiren/HCTZ: aliskiren produced a smaller reduction in SBP in the group of patients with reduced estimated GFR (<60 ml/min/1.73 m^2^) than in the group of patients with normal estimated GFR, and the difference between these reductions was significantly higher than provided by aliskiren/HCTZ (7.9 vs. 4.2 mmHg). All treatment regimens (excluding monotherapy with amlodipine and HCTZ) showed a high correlation (with coefficients of 0.42–0.66) between the afferent arteriole diameter and SBP response. For HCTZ alone, the correlation coefficient was lower (0.29), while for amlodipine alone it was insignificant (0.07). In addition, HCTZ-based regimens had a high negative correlation between fractional proximal sodium reabsorption and SBP/DBP reduction (with coefficients from −0.79 to −0.65 for SBP and coefficients from −0.67 to −0.45 for DBP). This result is due to the fact that less sodium is delivered to the distal tubules and, therefore, the thiazide diuretic effect is less pronounced.

RAAS parameters: The model predicted a high positive correlation between baseline PRA and DBP reduction for RAAS-acting medicines. At the same time, the use of amlodipine and HCTZ in combination with these drugs decreased the absolute values of the correlation coefficients. Interestingly, the correlation coefficients between PRA and SBP reduction with RAAS agents alone turned out to be insignificant. These results are consistent with the study by Ikeda et al. (1997), which showed a statistically significant correlation between PRA and reduction in peak and trough DBP in patients with essential hypertension treated with losartan, and the study by Nussberger et al. (2007), who found no significant correlations between PRA and SBP response in patients with mild-to-moderate hypertension receiving aliskiren or irbesartan. In addition, we found a number of studies demonstrating positive correlation between pre-treatment PRA and the fall in mean arterial pressure for different antihypertensive drugs, including enalapril (Fouad et al., 1984), captopril (Nakano et al., 1997), and olmesartan (Ono et al., 2012). Our model satisfies these data, since mean arterial pressure reduction shows similar correlations with PRA as for DBP (Supplementary Table S11).

### 3.5 Limitations of the study

The main limitation of the work is related to the generation of a virtual population. We consider a large number of constraints imposed on the parameters and variables of the model (Supplementary Tables S1, S2). But this is not enough to cut off all virtual patients with unrealistic dynamics. As a solution to the constraint optimization problem, we can obtain a parameterization of the model that demonstrates an excessive increase in systolic blood pressure (more than 25 mmHg) with an increase in sodium intake to a value of 0.243 mEq/min, or parameterization that leads to incorrect model dynamics when simulating antihypertensive therapy (for example, an increase in heart rate during treatment with bisoprolol). In the current work, we removed such parameterizations with additional checks. However, their isolation at the optimization stage could significantly speed up the process of generating virtual patients. One of the ways to solve this problem is to look for correlations between the domain (and/or range) of the model and the resulting dynamics of control variables in response to external stimuli (a sharp increase in sodium intake, activation of one of the antihypertensive treatment regimens, etc.). Adding appropriate constraints (determined from such correlations) to the optimization problem will result in valid virtual patients. However, the values of the correlation parameters must be close to unity, otherwise we risk losing some of the valid solutions. The second possible way to solve this problem is to introduce simulation experiments (test cases) into the optimization problem. In this case, each potential solution at all iterations of the optimization algorithm will be checked not only for the fulfillment of the established parametric constraints, but also for completion of test cases. This approach avoids the loss of solutions, as in the case of insufficiently high values of correlation parameters, but requires a change in the system implementation of optimization algorithms in BioUML.

Another limitation of the study is the complexity of the considered model, which is directly related to the cost of resources and time and is reflected in the speed of generation of virtual patients. However, the constrained optimization algorithm used to obtain a single patient is easily parallelized, as is the process of creating a large population. Therefore, with the development of computing technologies and the emergence of more powerful servers with multi-core processors, this problem will become less acute. Low-dimensional models certainly have a number of benefits besides saving time and enhancing productivity. In particular, they are easier to understand and interpret. And in some cases, they reproduce experimental data and make reasonable predictions just as well as more complex models (Kutumova et al., 2013). Therefore, simple mechanistic models are still useful and relevant for solving applied problems related to the study of pathological conditions of human physiology. However, the main disadvantage of simple models is the loss of biological information. Thence, we believe that over time, physiological models will continue to evolve towards greater complexity and closer to reality.

## 4 Conclusion

People differ in their genomes, environments, behaviours, and disease histories—all of these differences lead to variations in response to a particular medical treatment. Thus, true personalization of drug therapies should rely on “virtual patient” models implemented at the level of abstraction required for a specific pathology (Lehrach, 2016). Here we present a technology for constructing a virtual patient and an algorithm for generating a virtual population with varying values of the necessary parameters. This technology is based on the previously developed agent-based modular model of the cardiovascular and renal systems (Kutumova et al., 2021) and can be used to optimize the choice of drug therapy for cardiovascular diseases. As an example, we considered approaches to the treatment of arterial hypertension using different pharmacological effects of medicines, including angiotensin II receptor blockade (losartan), calcium channel blockade (amlodipine), angiotensin-converting enzyme inhibition (enalapril), direct renin inhibition (aliskiren), the action of thiazide diuretics (hydrochlorothiazide), and β-blockade (bisoprolol). For all of these drugs, we determined target points in the model and developed pharmacodynamic functions of their effects. To calibrate therapy parameters, we used data from clinical trials found in the scientific literature. We then tested the resulting model on a population of virtual patients with uncomplicated arterial hypertension and made sure that it reasonably reproduces the dynamics after treatment in accordance with clinical observations.

### 4.1 What is next?

We consider each parameterization of the model in an equilibrium state and within physiological ranges as a virtual patient. To relate this concept to a real patient, we can adjust the model parameters so that the equilibrium dynamics reproduces laboratory measurements of desired physiological characteristics (obtained from blood pressure monitoring, electrocardiography, echocardiography, blood tests, etc.). However, these data give only a small part of the model quantities. To control the unknown parameters and variables, we can move from a single virtual patient to a virtual population and take into account significant variation in model values. Treatment simulation of such a population allows it to be divided into groups with a similar response to drugs. Thus, we can find out which patient characteristics contribute to the effectiveness (or failure) of antihypertensive therapy. Linking the model to real patients is the main direction of our future work.

## Data Availability

The datasets presented in this study can be found in online repositories. The names of the repository/repositories and accession number(s) can be found below: https://gitlab.sirius-web.org/virtual-patient/antihypertensive-treatment-modeling.

## Footnotes

1
https://www.ebi.ac.uk/biomodels/MODEL2202160001.
