# Supplementary material for: Mathematical modeling of antihypertensive therapy
Source: Front Physiol. 2022 Dec 14;13:1070115. doi: 10.3389/fphys.2022.1070115 (PMC9795234; doi:10.3389/fphys.2022.1070115)
Supplement: Supplementary file 1 [file DataSheet1.PDF]

Table S1[1]. List of fitting parameters and boundaries of the search space used to generate virtual patients with arterial hypertension.

№	Parameters	Notations in the model	Ranges[2]	Units
01	Total metabolic intensity	A_1	0.00032 – 0.00128	mL^{-1}
02	Sympathetic sensitivity of the systemic microvessels	A_3	0.1 – 0.6	$mL \cdot mmHg^{-1}$
03	Systemic arterial tone	A_9	0.07 – 0.09	$mmHg \cdot s \cdot mL^{-1}$
04	Normal level of vasopressin	C_{adh_norm}	1.0 – 13.3	$pg \cdot mL^{-1}$
05	Normal level of aldosterone	C_{al_norm}	70 – 300	$pg \cdot mL^{-1}$
06	Normal level of atriopeptin	C_{anp_norm}	7.4 – 152.0	$ng \cdot L^{-1}$
07	Oxygen capacity of hemoglobin	C_H	1.32 – 1.39	$mL \cdot g^{-1}$
08	Serum potassium	C_K	3.5 – 5.5	$mEq \cdot L^{-1}$
09	Cardiac output (in the renal submodel)	CO	2.51 – 9.00	$L \cdot min^{-1}$
10	Afferent arteriole diameter	d_{aa}	8.7 – 23.9	µm
11	Efferent arteriole diameter	d_{ea}	12.2 – 20.1	µm
12	Action threshold of the Frank-Starling law	$FS_{threshold0}$	0.0 – 40.0	mL
13	Basic systemic arterial elastance	G_{AL0}	0.33 – 1.67	$mmHg \cdot mL^{-1}$
14	Basic pulmonary arterial elastance	G_{AR0}	0.08 – 0.26	$mmHg \cdot mL^{-1}$
15	Left ventricular wall elasticity	G_{HL}	0.02 – 0.72	$mmHg \cdot mL^{-1}$
16	Right ventricular wall elasticity	G_{HR}	0.02 – 0.72	$mmHg \cdot mL^{-1}$
17	Basic elasticity of the systemic veins	G_{VL0}	0.01 – 0.05	$mmHg \cdot mL^{-1}$
18	Basic elasticity of the pulmonary veins	G_{VR0}	0.01 – 0.05	$mmHg \cdot mL^{-1}$
19	Plasma glucose	$glucose$	3.9 – 6.1	$mmol \cdot L^{-1}$
20	Hematocrit	Hct	M: 40 – 54; W: 36 – 48	%
21	Hemoglobin	He	M: 140 – 180; W: 120 – 160	$g \cdot L^{-1}$
22	Basic activity of the cardiac center	$Heart_{Base}$	0.01 – 1.00	–
23	The ratio of unstressed to stressed volume in the systemic arteries	k_{AL}	0.7 – 1.0	–
24	The ratio of unstressed to stressed volume in the pulmonary arteries	k_{AR}	0.7 – 1.0	–
25	The ratio of unstressed to stressed volume in the systemic veins	k_{VL}	0.7 – 1.0	–
26	The ratio of unstressed to stressed volume in the pulmonary veins	k_{VR}	0.7 – 1.0	–
27	The ratio of unstressed to stressed volume in the left ventricle	k_{HL}	0.0 – 0.3	–
28	The ratio of unstressed to stressed volume in the right ventricle	k_{HR}	0.0 – 0.3	–
29	Normal filtration coefficient	K_{FG_0}	0.0039 – 0.0162	$L \cdot min^{-1} \cdot mmHg^{-1}$
30	Inotropic status of the left ventricle	K_{L0}	0.5 – 0.8	–
31	Inotropic status of the right ventricle	K_{R0}	0.5 – 0.8	–
32	Afferent arteriole length	L_{aa}	101 – 123	µm
33	Efferent arteriole length	L_{ea}	124 – 152	µm
34	Initial value of the total exchangeable sodium	M_{sod}	2040 – 3950	mEq
35	Number of nephrons in the kidneys	$N_{nephrons}$	0.80*E*6 – 2.75*E*6	–

[1] The substantiation of the parameter ranges can be found in the supplementary file to our basic study [Kutumova et al., 2021].
[2] **M** = men; **W** = women.

36	Normal fractional distal tubule sodium reabsorption	n_{ε_dt}	0.3 – 0.7	–
37	Normal fractional collecting duct sodium reabsorption	n_{η_cd}	0.6 – 1.0	–
38	Normal fractional proximal sodium reabsorption	n_{η_pt}	0.67 – 0.97	–
39	Hydrostatic pressure in the Bowman's space	P_B	10.0 – 15.0	mmHg
40	Normal value of the glomerular hydrostatic pressure	P_{gh_norm}	48.0 – 63.0	mmHg
41	Initial value of the glomerular capillary oncotic pressure	P_{go}	23.6 – 34.0	mmHg
42	Renal venous pressure	P_v	2.0 – 6.0	mmHg
43	Normal plasma renin concentration	PRC_{nom}	3.42 – 69.4	$pg \cdot mL^{-1}$
44	Nominal resistance of the interlobar, arcuate and interlobular arteries	$R_{preglom\_0}$	7.0 – 28.0	$mmHg \cdot min \cdot l^{-1}$
45	Renal venous resistance	R_v	11.3 – 20.1	$mmHg \cdot min \cdot l^{-1}$
46	Nominal body oxygen demand	RO_{20}	2.52 – 5.88	$mL \cdot s^{-1}$
47	Arterial oxygen saturation	SpO_2	0.92 – 0.99	–
48	Total protein	TP	60.0 – 86.0	$g \cdot L^{-1}$
49	Plasma urea concentration	$urea$	1.8 – 7.1	$mmol \cdot L^{-1}$
50	Initial value of the total body water	TBW	$f(0.9 \cdot N_{BV}) - f(1.1 \cdot N_{BV})$[3]	L
51	Initial value of the venous oxygen content	VO_2	0.0855 – 0.1848	–
52	The equilibrium ratio of plasma renin activity to plasma renin concentration	X_{PRC_PRA}	0.61 – 1.42	$fmol \cdot min^{-1} \cdot pg^{-1}$
53	Basic conductivity of the systemic microvessels	Y_{ALVL0}	0.5 – 2.0	$mL \cdot s^{-1} \cdot mmHg^{-1}$
54	Basic conductivity of the pulmonary microvessels	Y_{ARVR0}	9.0 – 21.0	$mL \cdot s^{-1} \cdot mmHg^{-1}$
55	Nominal value of the macula densa sodium flow rate	$\Phi_{md\_sod\_0}$	1.0 – 4.0	$mEq \cdot min^{-1}$
56	Sodium intake	Φ_{sodin}	0.0280 – 0.2088	$mEq \cdot min^{-1}$
57	Normal value of the water intake	Φ_{win_norm}	0.00096 – 0.00312	$L \cdot min^{-1}$

[3] To calculate TBW, we used the formula $f(N_{BV}) = (N_{BV} - 650)/111.5$ [Moore, 1967], where N_{BV} is the estimate defined by the Nadler equation [Nadler et al., 1962]. We considered $N_{BV} \pm 10\%$ as the range of total blood volume in normal humans.

Table S2[4]**.** List of constraints imposed on model variables in virtual hypertensive patients.

№	Variables	Notations/formulas in the model	Ranges[5]	Units
01	Arterial oxygen content	AO_2	0.145 – 0.244	–
02	Plasma angiotensin (1-7)	$ANG17$	12.7 – 34.9	$fmol \cdot mL^{-1}$
03	Plasma angiotensin I	$ANGI$	2.5 – 15.0	$fmol \cdot mL^{-1}$
04	Plasma angiotensin II	$ANGII$	3.3 – 7.1	$fmol \cdot mL^{-1}$
05	Plasma vasopressin	C_{adh}	1.0 – 13.3	$pg \cdot mL^{-1}$
06	Plasma aldosterone	C_{al}	70 – 300	$pg \cdot mL^{-1}$
07	Plasma atriopeptin	C_{anp}	7.4 – 152.0	$ng \cdot L^{-1}$
08	Plasma sodium	C_{sod}	137 – 147	$mEq \cdot L^{-1}$
09	Cardiac output (in the heart submodel)	CO	2.51 – 9.00	$L \cdot min^{-1}$
10	Ejection fraction	EF	50 – 80	%
11	Blood flow in the systemic microvessels	F_{ALVL}	> 0.0	$mL \cdot s^{-1}$
12	Blood flow in the pulmonary microvessels	F_{ARVR}	> 0.0	$mL \cdot s^{-1}$
13	Transaortic flow peak rate	F_{HLAL_p}	347.0 – 677.0	$mL \cdot s^{-1}$
14	Transpulmonary flow peak rate	F_{HRAR_p}	264.5 – 793.0	$mL \cdot s^{-1}$
15	Active peak filling rate (right ventricle)	F_{VLHR_ap}	M: 23 – 947; W: 54 – 680	$mL \cdot s^{-1}$
16	Early peak filling rate (right ventricle)	F_{VLHR_ep}	M: 8 – 814; W: -17 – 701	$mL \cdot s^{-1}$
17	The ratio of early to active peak filling rates (right ventricle)	F_{VLHR_ep}/F_{VLHR_ap}	M: -0.5 – 2.5; W: -0.4 – 2.5	–
18	Active peak filling rate (left ventricle)	F_{VRHL_ap}	M: 99 – 647; W: 58 – 508	$mL \cdot s^{-1}$
19	Early peak filling rate (left ventricle)	F_{VRHL_ep}	M: 21 – 1034; W: -13 – 967	$mL \cdot s^{-1}$
20	The ratio of early to active peak filling rates (left ventricle)	F_{VRHL_ep}/F_{VRHL_ap}	M: 0.3 – 5.9; W: 0.3 – 6.6	–
21	Systemic arterial elastance	G_{AL}	0.33 – 1.67	$mmHg \cdot mL^{-1}$
22	Pulmonary arterial elastance	G_{AR}	0.08 – 0.26	$mmHg \cdot mL^{-1}$
23	Glomerular filtration rate	GFR	0.060 – 0.135	$L \cdot min^{-1}$
24	Total exchangeable sodium	M_{sod}	2040 – 3950	mEq
25	Normal fractional sodium reabsorption in the distal tubule and subsequent parts of the nephron	$n_{\varepsilon\_dt} + n_{\eta\_cd} - n_{\eta\_cd} \cdot n_{\varepsilon\_dt}$	0.78 – 0.98	–
26	Plasma osmolality	$osmolality$	275 – 295	$mOsmol \cdot kg^{-1}$
27	Diastolic pulmonary arterial pressure	P_{AR_D}	4.0 – 12.0	mmHg
28	Systolic pulmonary arterial pressure	P_{AR_S}	15.0 – 30.0	mmHg
29	Glomerular hydrostatic pressure	P_{gh}	48.0 – 63.0	mmHg
30	Glomerular capillary oncotic pressure	P_{go}	23.6 – 34.0	mmHg
31	Left ventricular diastolic pressure	P_{HL_D}	1.0 – 18.0	mmHg
32	Left ventricular systolic pressure	P_{HL_S}	100 – 186	mmHg
33	Right ventricular diastolic pressure	P_{HR_D}	0.0 – 10.0	mmHg
34	Right ventricular systolic pressure	P_{HR_S}	15.0 – 30.0	mmHg

[4] The substantiation of the ranges can be found in the supplementary file to our basic study [Kutumova et al., 2021].

[5] **M** = men; **W** = women.

35	Systemic venous pressure	P_{VL}	1.0 – 10.0	mmHg
36	Pulmonary venous pressure	P_{VR}	3.0 – 20.0	mmHg
37	Plasma renin activity	PRA	12.0 – 38.0	$fmol \cdot mL^{-1} \cdot min^{-1}$
38	Plasma renin concentration	PRC	3.42 – 69.4	$pg \cdot mL^{-1}$
39	Resistance of the afferent vessels (arteries and arterioles)	R_{a_dyne}	3000 – 25000	$dyn \cdot s \cdot cm^{-5}$
40	Resistance of the efferent arterioles	R_{e_dyne}	1350 – 3400	$dyn \cdot s \cdot cm^{-5}$
41	Resistance of the interlobar, arcuate and interlobular arteries	$R_{preglom}$	7.0 – 28.0	$mmHg \cdot min \cdot L^{-1}$
42	Renal blood flow	RBF	0.623 – 1.730	$L \cdot min^{-1}$
43	Renal vascular resistance	RVR	55.0 – 190.0	$mmHg \cdot min \cdot L^{-1}$
44	Total blood volume	V	$0.9 \cdot N_{BV}$ – $1.1 \cdot N_{BV}$[6]	mL
45	Left ventricular end-diastolic volume	V_{HL_KD}	M: 67 – 155; W: 56 – 104	mL
46	Left ventricular end-systolic volume	V_{HL_KS}	M: 22 – 58; W: 19 – 49	mL
47	Right ventricular end-diastolic volume	V_{HR_KD}	M: 124 – 256; W: 78 – 218	mL
48	Right ventricular end-systolic volume	V_{HR_KS}	M: 38 – 118; W: 20 – 92	mL
49	Venous oxygen content	VO_2	0.0855 – 0.1848	–
50	Systemic vascular resistance	$1/Y_{HLAL}+1/Y_{ALVL}+1/Y_{VLHR}$	0.5271 – 1.9608	$s \cdot mmHg \cdot mL^{-1}$
51	Pulmonary vascular resistance	$1/Y_{HRAR}+1/Y_{ARVR}+1/Y_{VRHL}$	0.0151 – 0.1353	$s \cdot mmHg \cdot mL^{-1}$
52	Fractional proximal sodium reabsorption	$\eta_{pt\_sodreab}$	0.67 – 0.97	–
53	Fractional sodium reabsorption in the distal tubule and subsequent parts of the nephron	$\eta_{dt\_sodreab} + \eta_{cd\_sodreab} - \eta_{dt\_sodreab} \cdot \eta_{cd\_sodreab}$	0.78 – 0.98	–
54	Water intake	Φ_{win}	0.00096 – 0.00312	$L \cdot min^{-1}$

[6] N_{BV} is an estimate defined by the Nadler equation [Nadler et al., 1962]. We considered $N_{BV} \pm 10\%$ as the range of total blood volume in normal humans.

Table S3. Baseline characteristics of patient populations in clinical trials of antihypertensive drugs[7]

References	Treatment scheme	Number of persons (M/F)	Age (years)	SBP/DBP (mmHg)	HR (beats/ min)	BMI (kg/m^2)	Population
Abate et al., 1998	HCTZ 12.5 mg 8 weeks	28 (13/15)	77.0 ± 5.9	170.7 ± 11.1/ 99.9 ± 3.2	79.4 ± 7.9	–	Mild or moderate hypertension (DBP: 96 – 115 mmHg). Exclusion criteria: hypersensitivity to the test drugs, renal or hepatic failure, severe hypertension, leukopenia, heart failure, angina pectoris, idiopathic hypercalciuria, recent stroke or myocardial infarction (within the past 3 months), uncontrolled diabetes, atrial fibrillation, or orthostatic hypotension.
Agabiti Rosei et al., 2005	Enalapril 20 mg 12 weeks	133 (73/60)	53 (22 – 78)	151 ± 11/ 96 ± 6	–	26 (19 – 30)	Mild to moderate essential hypertension (SBP: 140 – 179 mmHg, DBP: 90 – 109 mmHg). Exclusion criteria: grade 3 hypertension or orthostatic hypotension; history of cardiovascular or cerebrovascular ischaemic events in the last 6 months; history of intracerebral haemorrhage or subarachnoid haemorrhage in the last 6 months; clinically significant cardiac valvular disease; known hypersensitivity to enalapril or nifedipine or to ACE inhibitors or dihydropyridine calcium antagonist; uncorrected hypokalaemia or hyperkalaemia; second or third degree heart block in the absence of a cardiac pacemaker; evidence of clinically significant cardiac arrhythmia; BMI > 31 kg/m^2; unstable type 1 diabetes mellitus; concomitant treatment with potassium sparing diuretics; gastrointestinal disease resulting in malabsorption; liver disease; transaminase levels > 2.5 times the upper limit of normal; renal failure; serum creatinine > 2.0 mg/dl; any malignant disease that has required treatment within the last 5 years; dementia or psychosis; history of non-compliance, alcoholism or drug abuse; treatment with any other investigational drug in the 30 days prior to entering the study; any condition which in the opinion of the investigator could interfere with the study results or be considered detrimental to the patient's welfare; women of childbearing potential with no adequate contraception.
Brown et al., 2011	Aliskiren 150 mg 8 weeks	318 (164/154)	58.4 ± 10.8	161.2 ± 8.5/ 92.0 ± 10.6	–	29.5 ± 5.2	Essential hypertension (SBP: 150 – 180 mmHg, DBP < 110 mmHg). Possible concomitant diseases: diabetes. Exclusion Criteria: severe hypertension, pregnant or nursing (lactating) women, pre-menopausal women not taking accepted form of birth control, serum potassium ≥ 5.5 mEq/L, history of cardiovascular conditions, uncontrolled Type 1 or Type 2 diabetes mellitus, hypersensitivity to renin inhibitors, calcium channel blockers, or to drugs with similar chemical structures.
	Amlodipine 5 mg 8 weeks	316 (156/160)	58.1 ± 10.9	161.1 ± 8.2/ 93.0 ± 9.1	–	29.8 ± 5.7	
Corea et al., 1996	Amlodipine 5 mg 8 weeks	84 (48/36)	52.9 ± 8.9	161.8 ± 12.8/ 101.8 ± 4.5	73.3 ± 9.6	–	Mild to moderate essential hypertension (DBP: 95 – 120 mmHg). Exclusion criteria: overt heart failure; a history of heart failure or myocardial infarction in the preceding 3 months; second- or third-degree heart block; malignant hypertension; evidence of significant hepatic, renal, or gastrointestinal disease; pregnancy; and use of estrogen-progestogen preparations; intolerable adverse events, lack of therapeutic response (DBP > 120 mm Hg).
Cushman et al., 2012	HCTZ 12.5 mg 4 weeks	128 (66/62)	77.7 ± 4.8	164.6 ± 12.0/ 85.6 ± 9.1	71.2 ± 11.3	28.6 ± 4.9	Primary systolic hypertension (SBP: 150 – 200 mmHg). Possible concomitant diseases: diabetes. Exclusion criteria: recent use of investigational drugs, history of hypersensitivity to drugs in similar chemical classes, inability to discontinue prior antihypertensive medications, SBP ≥ 160 mmHg despite ≥ 3 antihypertensive drugs at screening, DBP ≥ 120 mmHg at any time during the screening or washout phases, known secondary hypertension, clinically significant cardiac arrhythmias or cardiac valvular disease, history or symptoms of chronic heart failure, orthostatic hypotension, uncontrolled diabetes, malignancies, significant autoimmune disorders, acute gout within the previous year, or renal, pancreatic, or hepatic impairment, recent history (< 6 months of screening) of stroke, transient ischemic attack, myocardial infarction, significant coronary artery disease, or atherosclerotic vascular disease.

[7] **SBP** = systolic blood pressure; **DBP** = diastolic blood pressure; **HR** = heart rate; **BMI** = body mass index; **M** = male; **F** = female; **ACE** = angiotensin-converting enzyme; **ARB** = angiotensin-receptor blocker; **A150** = aliskiren 150 mg; **A300** = aliskiren 300 mg; **B5** = bisoprolol 5 mg, **L50** = losartan 50 mg; **n.s.** = not specified. The data are mean ± standard deviation or mean (range).

Derosa et al., 2014	Enalapril 20 mg 24 months	120 (58/62)	57.3 ± 6.1	155.6 ± 9.1/ 98.5 ± 7.2	–	26.1 ± 3.3	First diagnosis of essential hypertension (DBP: 90 – 110 mmHg, SBP: 140 – 180 mmHg), patients naive to antihypertensive treatment. <u>Exclusion criteria</u>: secondary hypertension, severe hypertension (SBP > 180 mmHg or DBP > 110 mmHg), hypertrophic cardiomyopathies due to aetiologies other than hypertension, history of heart failure or a left ventricular ejection fraction ≤ 50%, history of angina, stroke, transient ischaemic cerebral attack, coronary artery bypass surgery or myocardial infarction any time prior to visit 1, concurrent symptomatic arrhythmia, liver dysfunction (AST or ALT values exceeding 2-fold the upper limit), creatinine > 1.5 mg/dL and known hypersensitivity to the study drugs, pregnant women, women of childbearing potential, endocrine, infective or inflammatory disorders, patients taking anti-inflammatory medications.
Fagher et al., 1990	Enalapril 20 mg 4 weeks	22 (17/5)	37-59	163 ± 15/ 108 ± 7	75 ± 11	–	Newly discovered essential hypertension
Fermé et al., 1990	Enalapril 20 mg 4 weeks	31 (n.s.)	55 ± 2	172 ± 3/ 104 ± 1	77 ± 2	–	Mild to moderate arterial hypertension (DBP: 95 – 115 mmHg)
Gradman et al., 2005	Aliskiren 150 mg 8 weeks	127 (73/54)	55.0 ± 12.5	151.3 ± 11.1/ 98.8 ± 3.4	72.9 ± 9.4	30.7 ± 6.4	Mild-to-moderate essential hypertension (DBP: 95 – 110 mmHg). <u>Exclusion criteria</u>: severe hypertension (DBP ≥ 110 mmHg or SBP ≥ 180 mmHg), secondary hypertension, type 1 diabetes mellitus, type 2 diabetes mellitus with poor glucose control (HbA1c > 8% at screening), a history of cardiovascular disease (heart failure, myocardial infarction, unstable angina, transient ischemic cerebral attack), a history of malignancy or other life-threatening disease, any medical or surgical condition that might significantly alter the absorption, distribution, metabolism, or excretion of study drugs.
	Aliskiren 300 mg 8 weeks	130 (55/75)	56.0 ± 10.2	152.1 ± 10.2/ 98.8 ± 3.4	72.2 ± 8.2	30.5 ± 5.8	
Koh et al., 2004	Losartan 100 mg 2 months	47 (20/27)	57 ± 14	145 ± 14/ 89 ± 7	75 ± 14	25.2 ± 0.5	Hypercholesterolemic, hypertensive patients (LDL cholesterol levels ≥ 100 mg/dL, SBP ≥ 140 mmHg, DBP ≥ 90 mmHg). <u>Exclusion criteria</u>: severe hypertension, unstable angina, or acute myocardial infarction.
Kwakernaak et al., 2017	Aliskiren 300 mg 6 weeks	15 (15/0)	58 ± 12	149 ± 19/ 93 ± 12	–	30 ± 4	Essential hypertension (WHO criteria; either treated with antihypertensive medication or untreated ambulant SBP ≥ 140 mmHg and/or DBP ≥ 90 mmHg), weight excess (BMI: 27 – 35 kg/m^2), normal renal function (endogenous creatinine clearance ≥ 90 mL/min/1.73m^2), and normo- or microalbuminuria (urinary albuminuria excretion < 300 mg/day). <u>Exclusion criteria</u>: off-treatment SBP ≥ 180 mmHg and DBP ≥ 110 mmHg, history of cardiovascular disease (myocardial infarction, angina pectoris, percutanous transluminal coronary angioplasty, coronary artery bypass grafting, stroke, heart failure of stage I-IV of the New York Heart Association classification), diabetes mellitus, active malignancy, any medication and/or surgical or medical condition that might alter absorption, distribution, metabolism, or excretion of medication, history of hypersensitivity or contraindication to trial medication or radio-labeled tracers, history of angioedema, autonomic dysfunction, participation in any other clinical investigation within three months prior to start of the trial, blood or plasma donation within 3 months prior to initial dosing, and history of either drugs or alcohol abuse.
Lee et al., 2012	Losartan 50 mg 4 weeks	238 (167/71)	53.58 ± 9.61	149.17 ± 12.40/ 98.60 ± 5.68	–	–	Mild-to-moderate hypertension (DBP: 90 – 109 mmHg). <u>Exclusion criteria</u>: DBP < 90 mmHg or ≥ 110 mmHg; severe hypertension (SBP ≥ 200 mmHg); secondary hypertension; suspected severe systemic disease such as renal disease (serum creatinine ≥ 1.5 x upper limit of normal), gastrointestinal disorder, hematology disorder, or liver disease (aspartate transaminase and alanine transaminase ≥ 2 x upper limit of normal) that might affect the absorption, distribution, metabolism, and excretion of the study drugs; severe insulin-dependent diabetes or uncontrolled diabetes (glycated hemoglobin > 9%), patients whose usage or dose of an oral hypoglycemic agent was modified within the previous 12 weeks or patients who have experienced insulin administration; history of myocardial infarction or severe coronary arterial disease within the previous 6 months; clinically significant

							congestive heart failure or valvular heart disease; wasting disorder, autoimmune disease, or connective tissue disease; hepatitis B or C (including carrier); positive status for HIV; clinically significant abnormal laboratory test findings; patients under treatment with any medicine affecting blood pressure; patients with an allergic response or contraindication to angiotensin II antagonists; pregnant or breastfeeding women or women of child-bearing age not sterilized or who had no intention of using a contraceptive measure; alcoholic patients or patients with history/suspicion of drug abuse; participation in another clinical study within the previous 12 weeks from the screening visit.
Lithell et al., 1987	Bisoprolol 5 mg 4 weeks	97 (51/46)	51.8 ± 9.6	165.1 ± 17.4/ 103.2 ± 5.8	75.1 ± 9.6	26.3 ± 4.5	Mild to moderate hypertension (DBP: 95 – 120 mmHg). Possible concomitant diseases: history of coronary heart disease (3 patients), cerebrovascular disease (2), peripheral vascular disease (5), history of migraine (3), Raynaud's syndrome (5). Exclusion criteria: heart failure or sino-atrial block, atrio-ventricular block of any degree, left bundle branch block or bradycardia at rest (< 55 beats/min), patients who had experienced a myocardial infarction or a cerebrovascular accident during the last six months, patients with bronchial asthma, impaired renal function (serum creatinine > 150 μmol/L) or insulin-dependent diabetes mellitus, presence of other serious diseases.
Mallion, 2007	Enalapril 20 mg 4 weeks	156 (76/80)	53.3 ± 10.3	160.3 ± 9.7/ 100.8 ± 4.0	73.6 ± 9.4	27.7 ± 4.5	Mild to moderate hypertension (DBP: 95 – 115 mmHg). Exclusion criteria: severe or secondary forms of hypertension, two antihypertensive agents or more at the initial screening visit, cardiovascular or renal complications, insulin-dependent diabetes, concomitant medications judged to interfere with the study drugs.
Martina et al., 1999	Amlodipine 5 mg 6 weeks	14 (11/3)	51 ± 9	145 ± 10/ 100 ± 5	76 ± 9	30 ± 3	Untreated mild-to-moderate hypertension (DBP: 95 – 115 mmHg). Exclusion criteria: previous antihypertensive treatment.
	Losartan 50 mg 6 weeks	11 (5/6)	47 ± 6	154 ± 11/ 102 ± 4	79 ± 7	31 ±5	
Narkiewicz, 2007	Losartan 50 mg 4 weeks	162 (68/94)	57.9 ± 9.4	157.9 ± 12.0/ 99 ± 3	–	28.6 ± 3.1	Mild to moderate hypertension (DBP: 95 – 100 mmHg). Exclusion criteria: severe or secondary forms of hypertension, two antihypertensive agents or more at the initial screening visit, cardiovascular renal complication, insulin-dependent diabetes, concomitant medications known to interfere with the study drugs.
Nedogoda et al., 2013	Enalapril 20 mg 24 weeks	30 (15/15)	47.8 ± 8.1	157 ± 3/ 98 ± 3	79 ± 5	30.9 ± 2.2	Overweight or obese patients (BMI ≥ 27 kg/m^2) with hypertension (SBP: 140 –160 mmHg, DBP: 90 – 100 mmHg). Possible concomitant diseases: hypercholesterolaemia, microalbuminuria, left ventricular hypertrophy, history of coronary artery disease. Exclusion criteria: age < 18 years; hypersensitivity, intolerance or contraindications to ACE inhibitors or ARBs; unstable angina; heart failure; renal or hepatic insufficiency; grade 2 or 3 arterial hypertension (≥ 160/100 mmHg); a history of stroke; diagnosed or suspected secondary hypertension; or a serious illness affecting their prognosis.
	Losartan 100 mg 24 weeks	30 (15/15)	46.7 ± 8.2	157 ± 4/ 98 ± 3	76 ± 5	29.4 ± 3.6	
Oparil et al., 1996	Amlodipine 5 mg 4 weeks	93 (49/44)	54 ± 12	154.9 ± 16.7/ 101.0 ± 5.1	–	–	Mild-to-moderate hypertension (DBP: 95 – 114 mmHg), no known drug hypersensitivity to the study medications. Exclusion criteria: significant cardiovascular, cerebrovascular, renal, or hepatic disease; recent myocardial infarction; secondary hypertension; clinically significant laboratory abnormalities; pregnant women, and women of childbearing age who were not using an approved method of contraception.
	Losartan 50 mg 4 weeks	97 (53/44)	55 ± 13	153.7 ± 15.5/ 101.1 ± 5.3	–	–	
Oparil et al., 2008	Losartan 50 mg 4 weeks; Losartan 100 mg 4 weeks	127 (48/79)	50.1 ± 10.4	151.6 ± 8.2/ 99.1 ± 4.2	–	37.0 ± 6.1	Hypertension (SBP: 140 – 180 mmHg, DBP: 95 – 115 mmHg), without diabetes (fasting glucose < 126 mg/dL), taking no more than two antihypertensive agents, BMI > 30 kg/m^2, waist circumference > 40 (males)/> 35 (females) inches. Exclusion criteria: secondary hypertension, clinically significant renal disease (serum creatinine > 1.5 mg/dL or proteinuria > 2+ by urine dipstick) were excluded from the study.

Pareek et al., 2016	HCTZ<br>12.5 mg<br>12 weeks	18<br>(8/10)	47.72 ± 10.37	149.87 ± 4.50/<br>93.39 ± 2.45	–	27.81 ± 5.08	Stage 1 essential hypertension (SBP: 140 – 159 mmHg, DBP: 90 – 99 mmHg).<br>Exclusion criteria: (among others) secondary hypertension; diabetes; hyperuricemia; gout; chronic kidney disease; parathyroid diseases; recent cardiovascular disease or cardiovascular accident; abnormal renal function (serum creatinine: > 1.5 mg/dl; blood urea nitrogen > 20 mg/dl), abnormal liver function (aspartate aminotransferase, alanine aminotransferase, total bilirubin, or alkaline phosphatase > 2.5 x the upper limit of normal values); electrolyte imbalance; hypercalcemia; hypophosphatemia; women who were pregnant, lactating, or of childbearing potential and not practicing contraception; alcoholics; participation in a clinical trial within 30 days prior to enrollment.
Paterna et al., 2007	Bisoprolol<br>5 mg<br>6 months	87<br>(46/41)	49 ± 11	145.0 ± 15.5/<br>88.9 ± 10.6	82.4 ± 14.2	–	Mild to moderate hypertension (DBP: 89 – 110 mmHg, SBP: 139 – 180 mmHg), absence of clinical findings suggestive of a secondary form of hypertension.<br>Exclusion criteria: other cardiovascular disease (defined as myocardial infarction or angina pectoris, heart block, valvular disease, or heart failure and claudication), concomitant left ventricular hypertrophy defined according to echocardiography criteria or other target organ damage (including Keith-Wegener hypertensive retinopathy), proteinuria or renal disease, insulin-dependent or insulin-independent diabetes mellitus, electrolyte imbalance, alcoholism, psychiatric problems, or contraindications for β-blockers.
Pool et al., 2007	Aliskiren<br>150 mg<br>8 weeks	178<br>(101/77)	56.2 ± 12.4	154.1 ± 12.7/<br>99.3 ± 3.7	72.3 ± 9.5	28.8 ± 4.2	Mild-to-moderate essential hypertension (DBP: ≥ 95 mmHg).<br>Possible concomitant diseases (number of patients in A150/A300 groups): diabetes (15/13); history of coronary artery disease (2/1), myocardial infarction (0/1), transient ischemic attack (2/0).<br>Exclusion criteria: severe hypertension (DBP ≥ 110 mmHg or SBP ≥ 180 mmHg), secondary hypertension, type 1 or uncontrolled (HbA1c > 8%) type 2 diabetes mellitus, history of severe cardiac or cerebrovascular disease, history of severe or life-threatening disease, and any medical or surgical condition that might alter the pharmacokinetics of study drugs, pregnant or lactating women.
	Aliskiren<br>300 mg<br>8 weeks	175<br>(100/75)	56.7 ± 11.9	152.9 ± 12.2/<br>99.1 ± 3.4	72.7 ± 9.2	29.5 ± 5.1	
Porthan et al., 2009	Amlodipine<br>5 mg<br>4 weeks	183<br>(183/0)	51 ± 6	152 ± 13/<br>100 ± 7	62 ± 7	26.5 ± 2.7	Prior antihypertensive drug treatment or DBP ≥ 95 mmHg, sinus rhythm.<br>Exclusion criteria: treatment with three or more antihypertensive drugs, secondary hypertension, drug-treated diabetes mellitus, congestive heart failure, coronary heart disease, cerebrovascular disease, kidney disease (serum creatinine > 115 μmol/L), obstructive pulmonary disease, a disease treated with corticosteroids, clinically significant liver disease, abuse of drugs or alcohol, BMI ≥ 32 kg/m^2; the subject was withdrawn during the study, if the blood pressure level increased to 200/120 mm Hg; complete bundle branch block.
	Bisoprolol<br>5 mg<br>4 weeks						
	Losartan<br>50 mg<br>4 weeks						
Puig et al., 2007	Enalapril<br>20 mg<br>4 weeks	62<br>32/30	65.4 ± 4.6	168 ± 6/<br>93 ± 9	–	28.3 ± 2.9	Hypertension (SBP: 160 – 179 mmHg, DBP < 110 mmHg, and mean daytime SBP ≥ 135 mmHg).<br>Exclusion criteria: severe hypertension, a history of cardio- or cerebrovascular complications or diabetes mellitus requiring drug treatment.
Sun et al., 2016	Bisoprolol<br>5 mg<br>12 months	32<br>(15/17)	59.2 ± 9.9	147 ± 13/<br>90 ± 7	74 ± 9	24.4 ± 3.4	Hypertension (SBP: 140 – 180 mmHg), renal failure (patients on hemodialysis for more than 6 months, who received erythropoietin dose adjusted to maintain hemoglobin between 110 and 120 g/L, and who achieved their dry weight assessed by bioimpedance method for at least 3 months).<br>Possible additional antihypertensive medication: used on top of baseline (number of patients in B5/L50 groups): felodipine (11/9), terazosin (2/2).<br>Exclusion criteria: clinical cardiovascular events within 6 months preceding entry into the study; diabetes mellitus (on the basis of past history and/or presence of overt fasting hyperglycemia of 7.0 mmol/L or greater); atrioventricular block and sinoatrial block, HR < 70 beats/min; chronic obstructive pulmonary disease; treatment with ACE inhibitors or angiotensin II receptor blockers during the past 6 months; age ≥ 75 years.
	Losartan<br>50 mg<br>12 months	33<br>(16/17)	57.1 ± 10.1	145 ± 11/<br>92 ± 6	76 ± 10	23.1 ± 3.7	

Tham et al., 1993	Amlodipine<br>5 mg<br>4 weeks	14<br>(n.s.)	53 ± 10	156 ± 15/<br>93 ± 7	77 ± 10	–	Essential hypertension (diastolic phase V blood pressure 95 – 110 mmHg).
Weber et al., 1995	Losartan<br>50 mg<br>4 weeks	21<br>(n.s.)	–	159.3 ± 16.6/<br>101.0 ± 4.9	–	–	Hypertension (DBP: 95 – 115 mmHg, 24-hour average DBP ≥ 85 mmHg), patients within 30% of their ideal body weights.<br>Exclusion criteria: secondary forms of hypertension; meaningful cardiopulmonary, renal, metabolic, or neurologic abnormalities; patients who required any form of concomitant cardiovascular medication or the long term use of any drugs that could affect blood pressure or other cardiovascular measurements.
	Losartan<br>100 mg<br>4 weeks	16<br>(n.s.)	–	150.9 ± 14.0/<br>102.3 ± 4.7	–	–	
Wing et al., 2003	HCTZ<br>12.5 mg<br>6 weeks	17<br>(9/8)	61–81<br>(median 68)	163 ± 4/<br>81 ± 8	69 ± 8	–	Isolated systolic hypertension (SBP: 160 – 210 mmHg, DBP < 95 mmHg).<br>Exclusion criteria: secondary hypertension of any aetiology; a past history of malignant hypertension or blood pressure outside the inclusion limits; myocardial infarction, coronary angioplasty or coronary artery bypass grafting within the previous 6 months; a stroke or transient ischaemic attack within the past year; evidence of drug allergy or other contraindication to the administration of angiotensin receptor antagonists or diuretics; any evidence of cardiac failure, clinically significant cardiac valvular disease, cardiomyopathy or unstable angina; previously known moderate to severe left ventricular dysfunction; significant gastrointestinal disorders that could interfere with drug absorption; any clinically significant abnormalities in haematological or biochemical profiles; a history of gout or plasma urate concentration > 0.45 mmol/L; any other significant illness likely to interfere with survival (e.g. malignancy), or if it was anticipated that they would have poor compliance with study procedures.

Table S4. Response of human physiology to antihypertensive therapy[8]

Drugs	Patients	Treatment period	References	PRA	PRC	ANGI	ANGII	PAC	SSC	HR	CO	GFR	FF	RPF	RBF	RVR	ECFV	RA
Aliskiren, 300 mg	Weight excess, hypertension	6 weeks	Kwakernaak et al., 2017	↓	↑			NS	NS			NS	↓	↑		↓	NS	
Aliskiren, 150 to 300 mg	Hypertension, chronic kidney disease	24 weeks	Bokuda et al., 2018	↓	↑			↓				NS						
Aliskiren, 37.5, 75, 150, 300 mg	Essential hypertension	4 weeks	Stanton et al., 2003	↓						NS								
Aliskiren, 300 mg	Type 2 diabetes, hypertension, albuminuria	2 months	Persson et al., 2009	↓	↑	↓	↓	NS				↓						
Aliskiren, 300 mg	Hypertension, chronic kidney disease	6 weeks	Siddiqi et al., 2011						NS			NS						
Aliskiren, 75, 150, 300 mg	Mild-to-moderate essential hypertension	8 weeks	Villamil et al., 2007	↓	↑													
Aliskiren, 75, 150, 300, 600 mg	Healthy individuals	24 hours	Fisher et al., 2008	↓	↑	↓	↓					NS		↑				
Aliskiren, 150 to 300 mg	Elderly hypertensive individuals	6 months	Okada et al., 2017								NS							
Aliskiren, 150 mg	Advanced diabetic nephropathy	2 months	Ogawa et al., 2011									↑						
Aliskiren, 150 to 300 mg	Heart failure with reduced ejection fraction and decreased glomerular filtration rate	26 weeks	Schroten et al., 2015									↓	↓		NS			
Aliskiren, 300 mg	Uncomplicated type 1 diabetes mellitus	4 weeks	Cherney et al., 2012				↓	NS		NS		NS	NS	NS/↑	NS	NS		
Aliskiren, 150 mg (with 80 mg of valsartan *vs*. valsartan alone)	Hypertension with ischemic heart disease	24 weeks	Ozeki et al., 2014	↓				NS		↓								
Amlodipine, 10 mg	Mild to moderate hypertension	4 weeks	Licata et al., 1993	NS				NS				NS	NS	↑	↑	↓		
Amlodipine, 5 to 10 mg	Hypertensive renal transplant recipients	4 weeks	Toupance et al., 1994						NS									
Amlodipine, 5 mg	Renal transplant patients with normal renal function treated with cyclosporin	6 weeks	Iñigo et al., 2001	NS			↑					↑	↑	NS				↓
Amlodipine, 5 to 15 mg	Mild to moderate essential hypertension	12 weeks	Higashi et al., 1998	NS			NS	NS		NS		NS	↓	↑		↓		

[8] **PRA** = plasma renin activity; **PRC** = plasma renin concentration; **ANGI** = plasma angiotensin I concentration; **ANGII** = plasma angiotensin II concentration; **PAC** = plasma aldosterone concentration; **SSC** = serum sodium concentration; **HR** = heart rate; **CO** = cardiac output; **GFR** = glomerular filtration rate; **FF** = filtration fraction; **RPF** = renal plasma flow; **RBF** = renal blood flow; **RVR** = renal vascular resistance; **ECFV** = extracellular fluid volume; **RA** = afferent arteriolar resistance; ↓ = decrease; ↑ = increase; **NS** = not significant.

Drugs	Patients	Treatment period	References	PRA	PRC	ANGI	ANGII	PAC	SSC	HR	CO	GFR	FF	RPF	RBF	RVR	ECFV	RA
Amlodipine, 5 to 10 mg	Essential hypertension	12 weeks	Tham et al., 1993							NS	NS							
Amlodipine, 5 to 10 mg	Mild to moderate essential hypertension	8 weeks	Delles et al., 2003									↑	↑	NS				↓
Amlodipine, 10 mg	Chronic kidney disease	8.5 hours	Morrone et al., 2003									↑	↓	↑		↓		
Amlodipine, 2.5 to 10 mg	Essential hypertension	6 weeks	Reams et al., 1987										NS		↑	↓	NS	
Amlodipine, 5 to 10 mg	Hypertensive patients with renal insufficiency following renal transplantation, who were maintained on a stable dose of cyclosporin A	8 weeks	Venkat-Raman et al., 1998									↑		↑				
Amlodipine, 5 mg	Cyclosporin A treated normotensive renal transplant patients with mild renal insufficiency	8 weeks	Venkat Raman et al., 1999									NS		↑				
Amlodipine, 10 mg	Mild to moderate essential hypertension	4 weeks	Ott et al., 2012									NS		NS				
Amlodipine, 5 mg	Non-diabetic patients with chronic renal failure	12 months	Satomura et al., 2009									NS	NS	NS	NS			NS
Bisoprolol, 2.5 to 5 mg	Hypertension	12 weeks	Eguchi et al., 2015						NS									
Bisoprolol, 10 mg	Normotensive and hypertensive subjects	1 week	Blumenfeld et al., 1999	↓			↓											
Bisoprolol, 10 mg	Essential hypertension	2 hours	Glück and Reubi, 1986	↓						↓		↓						
Bisoprolol, 5–10 mg	Drug-resistant hypertension	12 weeks	Williams et al., 2015						NS			↓						
Bisoprolol, 5 mg	Uncomplicated hypertension	1 year	Parrinello et al., 2009							↓	NS	NS	↓	NS	NS	NS		
Bisoprolol, 5 mg	Mild to moderate hypertension	6 months	Paterna et al., 2007							↓	NS	NS	NS	NS	NS	NS		
Bisoprolol, 10 mg	Essential hypertension	30 days	Honore, 1987							↓								
Bisoprolol, 10 mg	Sustained essential hypertension	4 weeks	Asmar et al., 1991							↓								
Bisoprolol, 10 mg	Uncomplicated essential hypertension	4 weeks	Leeman et al., 1993							↓	NS	NS			NS	↓		
Bisoprolol, 5 mg	Grade I to grade II essential hypertension	3 weeks	Suojanen et al., 2017							↓	NS							
Bisoprolol, 1.25 to 10 mg	Idiopathic pulmonary arterial hypertension	6 months	van Campen et al., 2016							↓	↓							

Drugs	Patients	Treatment period	References	PRA	PRC	ANGI	ANGII	PAC	SSC	HR	CO	GFR	FF	RPF	RBF	RVR	ECFV	RA
β-Blockers[9]	–	–	Mountokalakis, 1997; Bakris et al., 2006; Digne-Malcolm et al., 2016									↑/↓/NS	NS/↓	↑/↓/NS	↑/↓/NS	↑/↓/NS		
Enalapril, 20 mg	Normal subjects	4 hours	Nussberger et al., 1986	↑		↑	↓	↓										
Enalapril, 20 mg	Normal subjects	24 hours	Nussberger et al., 1992			↑	↓											
Enalapril, 20 mg	Healthy volunteers on a constant 100 mmol/d sodium diet	8 days	Nussberger et al., 2002[10]	↑	↑	↑	↓	↓										
Enalapril, 20 mg	Healthy normotensive subjects on a normal sodium diet	135 minutes	Schmitt et al., 1996	↑				NS				NS	NS	↑		↓		
Enalapril, 20 to 40 mg	Essential hypertension	8 weeks	Fagher et al., 1990	↑			↓	↓	NS									
Enalapril, 10 mg	Hypertension, chronic renal failure	6 weeks	Klein et al., 2003	↑													NS	
Enalapril, 20 mg (and concomitant medical therapy)	Moderate or severe chronic heart failure	8 weeks	Dickstein et al., 1995						NS									
Enalapril, 5 mg	Salt-resistant normotensive individuals	24 hours	Natarajan et al., 2016							NS			NS					
Enalapril, 2.5 to 20 mg	Long-term progression of left ventricular dilatation, asymptomatic systolic dysfunction	1 year, 13 – 45 months	Konstam et al., 1993							NS	NS							
Enalapril, 5 mg	Chronic heart failure	4 weeks	Kamishirado et al., 1997							NS								
Enalapril, 20 mg	Essential hypertension	4 weeks	Ajayi et al., 1989							↓								
Enalapril, 20 mg	Chronic kidney disease	4 hours	Morrone et al., 2003									↓	↓	↑		↓		
Enalapril, 10 to 40 mg	Essential hypertension	1, 6 weeks	Semplicini et al., 1986									NS	NS	NS			↑/NS	
Enalapril, 10 to 40 mg	Mild, uncomplicated hypertension	12 weeks	Simon et al., 1983[11]	↑								↑		↑				

[9] Some members of the β-blocker class increase GFR and RBF [Mountokalakis, 1997; Bakris et al., 2006; Digne-Malcolm et al., 2016]. Therefore, we considered an increase in these variables as a possible long-term effect of bisoprolol.

[10] This example is also described in [Wiggins and Kelly, 2009].

[11] This example is also described in [Digne-Malcolm et al., 2016].

Drugs	Patients	Treatment period	References	PRA	PRC	ANGI	ANGII	PAC	SSC	HR	CO	GFR	FF	RPF	RBF	RVR	ECFV	RA
Enalapril, 20 to 40 mg	Essential hypertension	8 weeks	Bauer, 1984 Bauer and Jones, 1984[11]									NS/↑	NS/↓	NS/↑	NS/↑	↓		
HCTZ, 6.25, 12.5, 25 mg	Mild-to-moderate essential hypertension	8 weeks	Villamil et al., 2007	↑	↑ (25 mg)													
HCTZ, 25 to 100 mg	Essential hypertension	3 months	Lijnen et al., 1981	↑		↑	↑	↑										
HCTZ, 25 mg	Mild to moderate hypertension without cardiac or renal disease	8 weeks	Scaglione et al., 1992						NS	NS		NS	NS	NS	NS	↓		
HCTZ, 25 mg	Mild to moderate hypertension without cardiac or renal disease	4 weeks	Scaglione et al., 1995						NS	NS		NS	NS	NS	NS	↓		
HCTZ, 25 mg	Healthy individuals	28 days	Devineni et al., 2014						NS									
HCTZ, 12.5 to 50 mg	Essential hypertension with or without left ventricular hypertrophy	6 months	Roman et al., 1998	↑			NS		NS									
HCTZ, 100 mg	Essential hypertension	9 months	van Brummelen et al., 1979		↑							NS	NS/↓		↑/ NS	↓		
HCTZ, 100 mg	Essential hypertension	1, 4, 12, 24, 36 weeks	van Brummelen et al., 1980		↑					↑/NS vs ↓[12]	NS/ ↓/↑							
HCTZ	Stage 1 or 2 hypertension	up to 2 year	Materson et al., 1998							NS/ ↓/↑								
HCTZ, 50 mg	Essential hypertension	4 weeks	Ajayi et al., 1989							↑								
HCTZ, 50 mg	Chronic kidney disease stages 1–3 and a urinary albumin-to-creatinine ratio over 300 mg/g	4 weeks	Morales et al., 2015		↑			NS	NS			↓						
HCTZ, 50 to 100 mg (mean, 86 mg)	Essential hypertension	6 months	Leth, 1970														↓/NS	
HCTZ, 25 mg	Severe renal failure, hypertension	1 month	Dussol et al., 2005									↓	NS	↓				
HCTZ, 25 mg	Hypertension and stage 4 or 5 chronic kidney disease	3 months	Dussol et al., 2012									NS	↓	NS		NS		
Thiazide diuretics	–	–	Mountokalakis, 1997									↑/ NS	NS		↑/ NS	↓		
Losartan, 100 mg	Healthy volunteers	7 days	Goldberg et al., 1993	↑			↑	NS										

[12] Responders *vs* non-responders

Drugs	Patients	Treatment period	References	PRA	PRC	ANGI	ANGII	PAC	SSC	HR	CO	GFR	FF	RPF	RBF	RVR	ECFV	RA
Losartan, 50 mg	Healthy normotensive subjects on a normal sodium diet	135 minutes	Schmitt et al., 1996	NS				↓				NS	NS	↑		↓		
Losartan, 100 mg	Essential hypertension	4 weeks	Stanton et al., 2003	↑						NS								
Losartan, 50 mg	Hypertensive subjects with type 2 diabetes and albumin excretion rates of 10–200 μg/min	4 weeks	Houlihan et al., 2002	↑			↑	↑/NS				NS	NS	NS				
Losartan, 100 mg	Hypertension, chronic renal failure	6 weeks	Klein et al., 2003	↑													NS	
Losartan, 5, 10, 25, 75, 150 mg	Congestive heart failure	24 hours	Gottlieb et al., 1993	↑			↑	↓										
Losartan, 25, 50 mg (and concomitant medical therapy)	Moderate or severe chronic heart failure	8 weeks	Dickstein et al., 1995						NS									
Losartan, 50 mg	Hypertension, IgA nephropathy	12 months	Ohashi et al., 2002	↑					NS			NS	NS	NS				
Losartan, 50 mg	Insulin-dependent diabetes mellitus, microalbuminuria	3, 7, 28 days	Buter et al., 2001	↑/ NS			↑/ NS	↓/ NS	NS			NS	↓	↑				
Losartan, 6.25 to 50 mg	Portal hypertensive patients with cirrhosis treated endoscopically after a variceal bleeding episode	6 weeks	González-Abraldes et al., 2001				↑	↓		NS	NS	NS/↓						
Losartan, 25 to 50 mg	Renal transplant patients with normal renal function treated with cyclosporin	6 weeks	Iñigo et al., 2001	↑			↑					NS	↓	NS				↓
Losartan, 50 to 100 mg (with possible HCTZ 12.5 mg once daily for the last 3 weeks)	Mild to moderate essential hypertension	9 weeks	Dobovisek et al., 2005							↓								
Losartan, 50 mg	Uncomplicated hypertension	1 year	Parrinello et al., 2009							NS	NS	NS	↓	NS	NS	NS		
Losartan, 50 mg	Hypertension without renal or cardiovascular disease	6 months, 1 year	Paterna et al., 2000									NS	↓	NS	NS	↓/NS		
Losartan, 12.5 to 50 mg	Essential hypertension	3 years	De Rosa et al., 2002									↑						

Table S5. Simulated effect of aliskiren 150 mg (A150), aliskiren 300 mg (A300), amlodipine 5 mg (A5), bisoprolol 5 mg (B5), enalapril 20 mg (E20), HCTZ 12.5 mg (H12.5), losartan 100 mg (L100), and losartan 50 mg (L50) on model variables in the virtual population of hypertensive patients (n = 186) during 4 weeks of treatment, mean ± SD.

Parameters	Baseline	A150	A300	E20	L50	L100	A5	B5	H12.5
Total body water	37.3 ± 4.3	37.3 ± 4.3	37.2 ± 4.3	37.1 ± 4.3	37.3 ± 4.3	37.2 ± 4.3	37.1 ± 4.3	37.9 ± 4.3	36.9 ± 4.3
Systolic blood pressure	154 ± 7	146 ± 7	143 ± 7	139 ± 8	146 ± 7	141 ± 8	144 ± 7	147 ± 8	141 ± 9
Diastolic blood pressure	101 ± 6	93 ± 6	91 ± 5	89 ± 5	93 ± 6	90 ± 5	93 ± 5	88 ± 6	92 ± 5
Heart rate	76 ± 7	74 ± 8	75 ± 8	77 ± 8	74 ± 8	76 ± 8	80 ± 7	66 ± 7	80 ± 10
Systemic vascular resistance	0.89 ± 0.09	0.88 ± 0.09	0.88 ± 0.09	0.87 ± 0.09	0.88 ± 0.09	0.87 ± 0.09	0.91 ± 0.10	0.86 ± 0.09	0.89 ± 0.09
Systolic pulmonary arterial pressure	17.4 ± 1.3	17.3 ± 1.4	17.1 ± 1.4	16.7 ± 1.4	17.3 ± 1.4	16.9 ± 1.4	17.4 ± 1.4	18.4 ± 1.6	16.6 ± 1.6
Diastolic pulmonary arterial pressure	11.2 ± 0.7	11.1 ± 0.7	11.0 ± 0.7	10.8 ± 0.7	11.1 ± 0.7	10.9 ± 0.7	11.3 ± 0.7	11.6 ± 0.9	10.8 ± 0.8
Pulmonary vascular resistance	0.100 ± 0.013	0.100 ± 0.013	0.100 ± 0.013	0.100 ± 0.013	0.100 ± 0.013	0.100 ± 0.013	0.101 ± 0.013	0.099 ± 0.013	0.100 ± 0.013
Stroke volume	70 ± 7	70 ± 7	69 ± 7	66 ± 8	70 ± 7	68 ± 7	67 ± 6	79 ± 9	64 ± 9
Ejection fraction	64 ± 7	64 ± 7	64 ± 7	63 ± 7	64 ± 7	63 ± 7	64 ± 7	67 ± 7	62 ± 7
Cardiac output	5.3 ± 0.5	5.2 ± 0.5	5.1 ± 0.5	5.1 ± 0.5	5.2 ± 0.5	5.1 ± 0.5	5.4 ± 0.5	5.2 ± 0.5	5.1 ± 0.5
LV peak systolic pressure	172.4 ± 7.0	163.4 ± 7.4	160.5 ± 7.5	156.3 ± 7.9	163.6 ± 7.4	158.9 ± 7.6	163.3 ± 6.9	165.2 ± 8.3	158.6 ± 8.9
LV end-diastolic pressure	8.0 ± 1.1	8.1 ± 1.1	8.0 ± 1.1	7.7 ± 1.1	8.1 ± 1.1	7.9 ± 1.1	7.8 ± 1.1	8.9 ± 1.3	7.5 ± 1.2
LV end-systolic volume	40 ± 11	40 ± 11	40 ± 11	40 ± 11	40 ± 11	40 ± 11	40 ± 10	41 ± 12	40 ± 11
LV end-diastolic volume	110 ± 13	111 ± 14	109 ± 14	106 ± 14	111 ± 14	108 ± 14	107 ± 13	120 ± 15	104 ± 15
RV peak systolic pressure	19.6 ± 1.4	19.5 ± 1.4	19.2 ± 1.4	18.9 ± 1.4	19.5 ± 1.4	19.1 ± 1.4	19.7 ± 1.4	20.6 ± 1.6	18.8 ± 1.7
RV end-diastolic pressure	5.9 ± 1.1	6.0 ± 1.1	5.9 ± 1.1	5.7 ± 1.1	6.0 ± 1.1	5.8 ± 1.1	5.7 ± 1.1	6.6 ± 1.2	5.5 ± 1.1
RV end-systolic volume	47 ± 12	48 ± 12	48 ± 12	47 ± 12	48 ± 12	47 ± 12	46 ± 11	51 ± 15	46 ± 11
RV end-diastolic volume	117 ± 14	118 ± 14	116 ± 14	113 ± 14	118 ± 14	115 ± 14	114 ± 14	130 ± 18	111 ± 16
Aortic valve peak flow	564 ± 65	561 ± 67	551 ± 67	535 ± 66	561 ± 67	545 ± 66	555 ± 63	610 ± 81	529 ± 74
Pulmonary valve peak flow	542 ± 75	539 ± 77	530 ± 76	516 ± 76	540 ± 77	525 ± 76	538 ± 74	582 ± 87	512 ± 83
LV early peak filling rate	628 ± 124	641 ± 127	627 ± 124	605 ± 120	641 ± 127	619 ± 123	630 ± 121	737 ± 157	590 ± 131
LV active peak filling rate	418 ± 42	389 ± 42	378 ± 42	361 ± 42	389 ± 42	371 ± 42	376 ± 37	402 ± 47	365 ± 43
RV early peak filling rate	571 ± 100	586 ± 103	577 ± 101	562 ± 98	587 ± 103	572 ± 100	585 ± 99	661 ± 123	549 ± 107
RV active peak filling rate	529 ± 55	493 ± 54	480 ± 53	461 ± 53	493 ± 54	473 ± 53	475 ± 49	505 ± 58	466 ± 55
Glomerular filtration rate	0.097 ± 0.018	0.097 ± 0.018	0.097 ± 0.018	0.097 ± 0.018	0.097 ± 0.018	0.097 ± 0.018	0.105 ± 0.016	0.097 ± 0.018	0.091 ± 0.022
Renal blood flow	1.10 ± 0.15	1.19 ± 0.16	1.19 ± 0.16	1.20 ± 0.16	1.19 ± 0.16	1.20 ± 0.16	1.20 ± 0.16	1.19 ± 0.16	1.24 ± 0.17
Filtration fraction	0.16 ± 0.04	0.15 ± 0.04	0.15 ± 0.04	0.15 ± 0.04	0.15 ± 0.04	0.15 ± 0.04	0.16 ± 0.04	0.15 ± 0.04	0.13 ± 0.04
Filtration coefficient	0.010 ± 0.003	0.013 ± 0.004	0.014 ± 0.004	0.017 ± 0.005	0.013 ± 0.004	0.015 ± 0.005	0.010 ± 0.003	0.014 ± 0.004	0.009 ± 0.003
Renal vascular resistance	105.8 ± 14.2	91.0 ± 12.7	88.7 ± 12.5	86.0 ± 12.3	91.1 ± 12.7	87.6 ± 12.4	89.7 ± 11.9	88.4 ± 12.5	85.0 ± 11.2
Afferent arteriolar resistance	4842 ± 825	4093 ± 747	3980 ± 739	3852 ± 734	4098 ± 747	3928 ± 737	3795 ± 679	3966 ± 738	3628 ± 635
Efferent arteriolar resistance	2606 ± 458	2117 ± 377	2037 ± 364	1942 ± 348	2121 ± 378	1999 ± 357	2342 ± 411	2029 ± 362	2084 ± 365
Macula densa sodium flow rate	2.0 ± 0.8	1.9 ± 0.8	1.9 ± 0.8	1.9 ± 0.8	1.9 ± 0.8	1.9 ± 0.8	2.0 ± 0.8	1.9 ± 0.8	1.3 ± 0.5
Fractional proximal sodium reabsorption	0.86 ± 0.06	0.86 ± 0.06	0.86 ± 0.06	0.86 ± 0.06	0.86 ± 0.06	0.86 ± 0.06	0.87 ± 0.06	0.86 ± 0.06	0.89 ± 0.06
Fractional distal sodium reabsorption	0.94 ± 0.03	0.94 ± 0.03	0.94 ± 0.03	0.94 ± 0.03	0.94 ± 0.03	0.94 ± 0.03	0.94 ± 0.03	0.94 ± 0.03	0.92 ± 0.04
Sodium	143.5 ± 1.3	143.5 ± 1.3	143.4 ± 1.3	143.4 ± 1.3	143.5 ± 1.3	143.4 ± 1.3	143.5 ± 1.3	143.4 ± 1.3	142.6 ± 1.3
Plasma renin activity	31.8 ± 3.6	12.7 ± 1.4	9.9 ± 1.1	217.7 ± 24.9	99.2 ± 11.3	163.3 ± 18.6	32.5 ± 3.7	9.6 ± 1.1	47.2 ± 5.3
Plasma renin concentration	33.1 ± 8.1	104.6 ± 25.6	143.3 ± 35.1	226.7 ± 55.5	103.3 ± 25.3	170.1 ± 41.7	33.8 ± 8.3	10.0 ± 2.4	49.1 ± 11.9
Plasma angiotensin I concentration	8.6 ± 1.0	3.4 ± 0.4	2.7 ± 0.3	77.4 ± 8.8	26.7 ± 3.0	44.0 ± 5.0	8.8 ± 1.0	2.6 ± 0.3	12.7 ± 1.4
Plasma angiotensin II concentration	5.4 ± 0.6	2.2 ± 0.2	1.7 ± 0.2	1.2 ± 0.1	19.1 ± 2.2	31.7 ± 3.6	5.5 ± 0.6	1.6 ± 0.2	8.0 ± 0.9
Plasma aldosterone concentration	182.4 ± 56.5	130.8 ± 40.5	119.5 ± 37.0	105.4 ± 32.7	131.3 ± 40.7	113.9 ± 35.3	183.7 ± 57.0	118.2 ± 36.7	190.4 ± 58.6

Table S6. Simulated effect of aliskiren 150 mg (A150), aliskiren 300 mg (A300), and their dual combinations with amlodipine 5 mg (A5), bisoprolol 5 mg (B5), and HCTZ 12.5 mg (H12.5) on model variables in the virtual population of hypertensive patients (n = 186) during 4 weeks of treatment, mean ± SD.

Parameters	Baseline	A150	A150/A5	A150/B5	A150/H12.5	A300	A300/A5	A300/B5	A300/H12.5
Total body water	37.3 ± 4.3	37.3 ± 4.3	37.0 ± 4.3	37.4 ± 4.3	36.8 ± 4.3	37.2 ± 4.3	36.8 ± 4.3	37.3 ± 4.3	36.6 ± 4.3
Systolic blood pressure	154 ± 7	146 ± 7	135 ± 7	137 ± 9	131 ± 9	143 ± 7	131 ± 7	134 ± 10	127 ± 9
Diastolic blood pressure	101 ± 6	93 ± 6	85 ± 5	83 ± 6	84 ± 5	91 ± 5	83 ± 5	82 ± 6	82 ± 5
Heart rate	76 ± 7	74 ± 8	80 ± 8	72 ± 8	81 ± 11	75 ± 8	82 ± 8	73 ± 9	83 ± 12
Systemic vascular resistance	0.89 ± 0.09	0.88 ± 0.09	0.89 ± 0.09	0.86 ± 0.09	0.87 ± 0.09	0.88 ± 0.09	0.89 ± 0.09	0.87 ± 0.09	0.87 ± 0.09
Systolic pulmonary arterial pressure	17.4 ± 1.3	17.3 ± 1.4	17.0 ± 1.4	17.3 ± 1.6	16.3 ± 1.8	17.1 ± 1.4	16.7 ± 1.4	17.2 ± 1.7	15.9 ± 1.8
Diastolic pulmonary arterial pressure	11.2 ± 0.7	11.1 ± 0.7	11.1 ± 0.7	11.1 ± 0.9	10.6 ± 0.9	11.0 ± 0.7	10.9 ± 0.7	11.0 ± 0.9	10.4 ± 0.9
Pulmonary vascular resistance	0.100 ± 0.013	0.100 ± 0.013	0.100 ± 0.013	0.100 ± 0.013	0.100 ± 0.013	0.100 ± 0.013	0.100 ± 0.013	0.100 ± 0.013	0.100 ± 0.013
Stroke volume	70 ± 7	70 ± 7	66 ± 7	71 ± 10	63 ± 10	69 ± 7	63 ± 7	70 ± 10	60 ± 10
Ejection fraction	64 ± 7	64 ± 7	63 ± 7	64 ± 7	61 ± 7	64 ± 7	62 ± 7	64 ± 7	60 ± 7
Cardiac output	5.3 ± 0.5	5.2 ± 0.5	5.2 ± 0.5	5.1 ± 0.5	5.0 ± 0.5	5.1 ± 0.5	5.2 ± 0.5	5.1 ± 0.5	4.9 ± 0.5
LV peak systolic pressure	172.4 ± 7.0	163.4 ± 7.4	153.1 ± 7.6	154.3 ± 9.4	148.2 ± 9.7	160.5 ± 7.5	149.6 ± 7.8	151.9 ± 10.1	144.4 ± 9.8
LV end-diastolic pressure	8.0 ± 1.1	8.1 ± 1.1	7.7 ± 1.1	8.2 ± 1.2	7.3 ± 1.2	8.0 ± 1.1	7.5 ± 1.1	8.1 ± 1.3	7.1 ± 1.2
LV end-systolic volume	40 ± 11	40 ± 11	40 ± 10	41 ± 11	40 ± 11	40 ± 11	40 ± 10	40 ± 11	40 ± 10
LV end-diastolic volume	110 ± 13	111 ± 14	105 ± 13	112 ± 15	103 ± 15	109 ± 14	103 ± 13	110 ± 16	100 ± 16
RV peak systolic pressure	19.6 ± 1.4	19.5 ± 1.4	19.2 ± 1.4	19.5 ± 1.7	18.4 ± 1.8	19.2 ± 1.4	18.9 ± 1.5	19.4 ± 1.7	17.9 ± 1.9
RV end-diastolic pressure	5.9 ± 1.1	6.0 ± 1.1	5.6 ± 1.0	6.0 ± 1.2	5.4 ± 1.2	5.9 ± 1.1	5.4 ± 1.0	5.9 ± 1.2	5.2 ± 1.1
RV end-systolic volume	47 ± 12	48 ± 12	46 ± 11	48 ± 12	46 ± 11	48 ± 12	46 ± 11	48 ± 12	46 ± 11
RV end-diastolic volume	117 ± 14	118 ± 14	112 ± 14	120 ± 17	109 ± 16	116 ± 14	109 ± 14	118 ± 17	106 ± 17
Aortic valve peak flow	564 ± 65	561 ± 67	539 ± 64	562 ± 78	513 ± 77	551 ± 67	524 ± 63	555 ± 80	497 ± 76
Pulmonary valve peak flow	542 ± 75	539 ± 77	524 ± 75	541 ± 86	498 ± 86	530 ± 76	511 ± 75	535 ± 88	483 ± 85
LV early peak filling rate	628 ± 124	641 ± 127	619 ± 120	664 ± 143	581 ± 135	627 ± 124	598 ± 116	654 ± 144	556 ± 131
LV active peak filling rate	418 ± 42	389 ± 42	343 ± 37	358 ± 47	332 ± 43	378 ± 42	330 ± 37	349 ± 48	318 ± 43
RV early peak filling rate	571 ± 100	586 ± 103	582 ± 99	611 ± 114	547 ± 111	577 ± 101	566 ± 97	605 ± 116	529 ± 109
RV active peak filling rate	529 ± 55	493 ± 54	435 ± 48	454 ± 58	425 ± 54	480 ± 53	421 ± 48	442 ± 59	409 ± 53
Glomerular filtration rate	0.097 ± 0.018	0.097 ± 0.018	0.106 ± 0.017	0.098 ± 0.019	0.093 ± 0.022	0.097 ± 0.018	0.106 ± 0.017	0.099 ± 0.019	0.093 ± 0.023
Renal blood flow	1.10 ± 0.15	1.19 ± 0.16	1.29 ± 0.17	1.20 ± 0.16	1.39 ± 0.19	1.19 ± 0.16	1.30 ± 0.17	1.20 ± 0.16	1.41 ± 0.19
Filtration fraction	0.16 ± 0.04	0.15 ± 0.04	0.15 ± 0.03	0.15 ± 0.04	0.12 ± 0.03	0.15 ± 0.04	0.15 ± 0.03	0.15 ± 0.04	0.12 ± 0.03
Filtration coefficient	0.010 ± 0.003	0.013 ± 0.004	0.013 ± 0.004	0.024 ± 0.007	0.011 ± 0.003	0.014 ± 0.004	0.014 ± 0.004	0.028 ± 0.009	0.012 ± 0.004
Renal vascular resistance	105.8 ± 14.2	91.0 ± 12.7	76.8 ± 10.7	82.4 ± 12.0	69.5 ± 9.5	88.7 ± 12.5	74.7 ± 10.5	80.8 ± 11.9	67.0 ± 9.3
Afferent arteriolar resistance	4842 ± 825	4093 ± 747	3158 ± 616	3683 ± 728	2833 ± 546	3980 ± 739	3054 ± 611	3605 ± 726	2702 ± 536
Efferent arteriolar resistance	2606 ± 458	2117 ± 377	1898 ± 338	1812 ± 324	1592 ± 282	2037 ± 364	1826 ± 326	1756 ± 314	1515 ± 269
Macula densa sodium flow rate	2.0 ± 0.8	1.9 ± 0.8	1.9 ± 0.8	1.9 ± 0.8	1.3 ± 0.5	1.9 ± 0.8	1.9 ± 0.8	1.9 ± 0.8	1.3 ± 0.5
Fractional proximal sodium reabsorption	0.86 ± 0.06	0.86 ± 0.06	0.87 ± 0.06	0.86 ± 0.06	0.89 ± 0.06	0.86 ± 0.06	0.87 ± 0.06	0.86 ± 0.06	0.89 ± 0.06
Fractional distal sodium reabsorption	0.94 ± 0.03	0.94 ± 0.03	0.94 ± 0.03	0.94 ± 0.03	0.92 ± 0.04	0.94 ± 0.03	0.94 ± 0.03	0.94 ± 0.03	0.92 ± 0.04
Sodium	143.5 ± 1.3	143.5 ± 1.3	143.4 ± 1.3	143.4 ± 1.3	142.6 ± 1.3	143.4 ± 1.3	143.4 ± 1.3	143.4 ± 1.3	142.6 ± 1.3
Plasma renin activity	31.8 ± 3.6	12.7 ± 1.4	13.0 ± 1.5	3.8 ± 0.4	18.9 ± 2.1	9.9 ± 1.1	10.1 ± 1.2	3.0 ± 0.3	14.7 ± 1.7
Plasma renin concentration	33.1 ± 8.1	104.6 ± 25.6	107.2 ± 26.2	31.5 ± 7.7	155.6 ± 37.8	143.3 ± 35.1	146.9 ± 35.9	43.1 ± 10.6	213.1 ± 51.7
Plasma angiotensin I concentration	8.6 ± 1.0	3.4 ± 0.4	3.5 ± 0.4	1.0 ± 0.1	5.1 ± 0.6	2.7 ± 0.3	2.7 ± 0.3	0.8 ± 0.1	4.0 ± 0.4
Plasma angiotensin II concentration	5.4 ± 0.6	2.2 ± 0.2	2.2 ± 0.2	0.6 ± 0.1	3.2 ± 0.4	1.7 ± 0.2	1.7 ± 0.2	0.5 ± 0.1	2.5 ± 0.3
Plasma aldosterone concentration	182.4 ± 56.5	130.8 ± 40.5	131.9 ± 40.9	88.3 ± 27.4	138.4 ± 42.6	119.5 ± 37.0	120.6 ± 37.4	82.6 ± 25.6	126.2 ± 38.9

Table S7. Simulated effect of losartan 50 mg (L50), losartan 100 mg (L100), and their dual combinations with amlodipine 5 mg (A5), bisoprolol 5 mg (B5), and HCTZ 12.5 mg (H12.5) on model variables in the virtual population of hypertensive patients (n = 186) during 4 weeks of treatment, mean ± SD.

Parameters	Baseline	L50	L50/A5	L50/B5	L50/H12.5	L100	L100/A5	L100/B5	L100/H12.5
Total body water	37.3 ± 4.3	37.3 ± 4.3	37.0 ± 4.3	37.4 ± 4.3	36.8 ± 4.3	37.2 ± 4.3	36.7 ± 4.3	37.3 ± 4.3	36.5 ± 4.3
Systolic blood pressure	154 ± 7	146 ± 7	135 ± 7	137 ± 9	131 ± 9	141 ± 8	129 ± 8	133 ± 10	125 ± 9
Diastolic blood pressure	101 ± 6	93 ± 6	85 ± 5	84 ± 6	84 ± 5	90 ± 5	83 ± 5	81 ± 6	81 ± 5
Heart rate	76 ± 7	74 ± 8	80 ± 8	72 ± 8	81 ± 11	76 ± 8	83 ± 8	74 ± 9	85 ± 12
Systemic vascular resistance	0.89 ± 0.09	0.88 ± 0.09	0.89 ± 0.09	0.86 ± 0.09	0.87 ± 0.09	0.87 ± 0.09	0.89 ± 0.09	0.87 ± 0.09	0.87 ± 0.09
Systolic pulmonary arterial pressure	17.4 ± 1.3	17.3 ± 1.4	17.0 ± 1.4	17.4 ± 1.6	16.3 ± 1.8	16.9 ± 1.4	16.5 ± 1.4	17.2 ± 1.7	15.6 ± 1.8
Diastolic pulmonary arterial pressure	11.2 ± 0.7	11.1 ± 0.7	11.1 ± 0.7	11.1 ± 0.9	10.6 ± 0.9	10.9 ± 0.7	10.8 ± 0.8	11.0 ± 0.9	10.3 ± 0.9
Pulmonary vascular resistance	0.100 ± 0.013	0.100 ± 0.013	0.100 ± 0.013	0.100 ± 0.013	0.100 ± 0.013	0.100 ± 0.013	0.100 ± 0.013	0.100 ± 0.013	0.100 ± 0.013
Stroke volume	70 ± 7	70 ± 7	66 ± 7	71 ± 10	63 ± 10	68 ± 7	62 ± 8	70 ± 11	58 ± 11
Ejection fraction	64 ± 7	64 ± 7	63 ± 7	64 ± 7	61 ± 7	63 ± 7	61 ± 7	64 ± 7	60 ± 7
Cardiac output	5.3 ± 0.5	5.2 ± 0.5	5.2 ± 0.5	5.1 ± 0.5	5.0 ± 0.5	5.1 ± 0.5	5.1 ± 0.5	5.1 ± 0.5	4.8 ± 0.5
LV peak systolic pressure	172.4 ± 7.0	163.6 ± 7.4	153.3 ± 7.5	154.4 ± 9.4	148.4 ± 9.7	158.9 ± 7.6	147.6 ± 8.0	150.8 ± 10.5	142.3 ± 9.9
LV end-diastolic pressure	8.0 ± 1.1	8.1 ± 1.1	7.7 ± 1.1	8.2 ± 1.2	7.4 ± 1.2	7.9 ± 1.1	7.3 ± 1.1	8.0 ± 1.3	6.9 ± 1.2
LV end-systolic volume	40 ± 11	40 ± 11	40 ± 10	41 ± 11	40 ± 11	40 ± 11	40 ± 10	40 ± 11	40 ± 10
LV end-diastolic volume	110 ± 13	111 ± 14	106 ± 13	112 ± 15	103 ± 15	108 ± 14	102 ± 13	110 ± 16	98 ± 16
RV peak systolic pressure	19.6 ± 1.4	19.5 ± 1.4	19.2 ± 1.4	19.5 ± 1.7	18.4 ± 1.8	19.1 ± 1.4	18.6 ± 1.5	19.3 ± 1.8	17.7 ± 1.9
RV end-diastolic pressure	5.9 ± 1.1	6.0 ± 1.1	5.6 ± 1.0	6.1 ± 1.2	5.4 ± 1.2	5.8 ± 1.1	5.3 ± 1.0	5.9 ± 1.2	5.1 ± 1.1
RV end-systolic volume	47 ± 12	48 ± 12	46 ± 11	48 ± 12	46 ± 11	47 ± 12	46 ± 11	48 ± 12	45 ± 11
RV end-diastolic volume	117 ± 14	118 ± 14	112 ± 14	120 ± 17	109 ± 16	115 ± 14	108 ± 14	117 ± 18	104 ± 17
Aortic valve peak flow	564 ± 65	561 ± 67	540 ± 64	563 ± 78	514 ± 77	545 ± 66	516 ± 63	552 ± 81	487 ± 76
Pulmonary valve peak flow	542 ± 75	540 ± 77	524 ± 75	541 ± 86	499 ± 86	525 ± 76	503 ± 74	533 ± 89	474 ± 85
LV early peak filling rate	628 ± 124	641 ± 127	620 ± 120	664 ± 143	582 ± 135	619 ± 123	584 ± 114	651 ± 147	541 ± 129
LV active peak filling rate	418 ± 42	389 ± 42	343 ± 37	359 ± 47	332 ± 43	371 ± 42	324 ± 37	344 ± 49	311 ± 43
RV early peak filling rate	571 ± 100	587 ± 103	582 ± 99	611 ± 114	548 ± 111	572 ± 100	556 ± 96	604 ± 118	517 ± 108
RV active peak filling rate	529 ± 55	493 ± 54	436 ± 48	455 ± 58	425 ± 54	473 ± 53	413 ± 48	437 ± 60	400 ± 53
Glomerular filtration rate	0.097 ± 0.018	0.097 ± 0.018	0.106 ± 0.017	0.098 ± 0.019	0.093 ± 0.022	0.097 ± 0.018	0.106 ± 0.017	0.099 ± 0.019	0.094 ± 0.023
Renal blood flow	1.10 ± 0.15	1.19 ± 0.16	1.29 ± 0.17	1.20 ± 0.16	1.39 ± 0.19	1.20 ± 0.16	1.30 ± 0.17	1.20 ± 0.16	1.41 ± 0.19
Filtration fraction	0.16 ± 0.04	0.15 ± 0.04	0.15 ± 0.03	0.15 ± 0.04	0.12 ± 0.03	0.15 ± 0.04	0.15 ± 0.03	0.15 ± 0.04	0.12 ± 0.03
Filtration coefficient	0.010 ± 0.003	0.013 ± 0.004	0.016 ± 0.004	0.023 ± 0.007	0.011 ± 0.003	0.015 ± 0.005	0.015 ± 0.005	0.031 ± 0.010	0.013 ± 0.004
Renal vascular resistance	105.8 ± 14.2	91.1 ± 12.7	76.9 ± 10.7	82.4 ± 12.0	69.6 ± 9.5	87.6 ± 12.4	73.6 ± 10.5	79.8 ± 11.8	65.8 ± 9.2
Afferent arteriolar resistance	4842 ± 825	4098 ± 747	3162 ± 617	3686 ± 728	2839 ± 547	3928 ± 737	3005 ± 609	3557 ± 723	2640 ± 533
Efferent arteriolar resistance	2606 ± 458	2121 ± 378	1901 ± 338	1815 ± 325	1595 ± 282	1999 ± 357	1791 ± 320	1723 ± 307	1480 ± 264
Macula densa sodium flow rate	2.0 ± 0.8	1.9 ± 0.8	1.9 ± 0.8	1.9 ± 0.8	1.3 ± 0.5	1.9 ± 0.8	1.9 ± 0.8	1.9 ± 0.8	1.3 ± 0.5
Fractional proximal sodium reabsorption	0.86 ± 0.06	0.86 ± 0.06	0.87 ± 0.06	0.86 ± 0.06	0.89 ± 0.06	0.86 ± 0.06	0.87 ± 0.06	0.86 ± 0.06	0.89 ± 0.06
Fractional distal sodium reabsorption	0.94 ± 0.03	0.94 ± 0.03	0.94 ± 0.03	0.94 ± 0.03	0.92 ± 0.04	0.94 ± 0.03	0.94 ± 0.03	0.94 ± 0.03	0.92 ± 0.04
Sodium	143.5 ± 1.3	143.5 ± 1.3	143.5 ± 1.3	143.4 ± 1.3	142.6 ± 1.3	143.4 ± 1.3	143.4 ± 1.3	143.4 ± 1.3	142.6 ± 1.3
Plasma renin activity	31.8 ± 3.6	99.2 ± 11.3	101.6 ± 11.5	29.9 ± 3.4	147.5 ± 16.6	163.3 ± 18.6	167.5 ± 19.0	49.1 ± 5.7	243.1 ± 27.5
Plasma renin concentration	33.1 ± 8.1	103.3 ± 25.3	105.8 ± 25.9	31.1 ± 7.6	153.6 ± 37.3	170.1 ± 41.7	174.5 ± 42.7	51.1 ± 12.5	253.1 ± 61.4
Plasma angiotensin I concentration	8.6 ± 1.0	26.7 ± 3.0	27.4 ± 3.1	8.0 ± 0.9	39.8 ± 4.5	44.0 ± 5.0	45.1 ± 5.1	13.2 ± 1.5	65.5 ± 7.4
Plasma angiotensin II concentration	5.4 ± 0.6	19.1 ± 2.2	19.5 ± 2.2	5.7 ± 0.7	28.4 ± 3.2	31.7 ± 3.6	32.5 ± 3.7	9.5 ± 1.1	47.2 ± 5.3
Plasma aldosterone concentration	182.4 ± 56.5	131.3 ± 40.7	132.4 ± 41.1	88.5 ± 27.5	138.9 ± 42.8	113.9 ± 35.3	114.9 ± 35.6	79.9 ± 24.8	120.1 ± 37.0

Table S8. Simulated effect of enalapril 20 mg (L100) and its dual combinations with amlodipine 5 mg (A5), bisoprolol 5 mg (B5), and HCTZ 12.5 mg (H12.5) on model variables in the virtual population of hypertensive patients (n = 186) during 4 weeks of treatment, mean ± SD.

Parameters	Baseline	E20	E20/A5	E20/B5	E20/H12.5
Total body water	37.3 ± 4.3	37.1 ± 4.3	36.6 ± 4.3	37.2 ± 4.3	36.3 ± 4.3
Systolic blood pressure	154 ± 7	139 ± 8	126 ± 8	131 ± 11	122 ± 10
Diastolic blood pressure	101 ± 6	89 ± 5	81 ± 5	80 ± 6	80 ± 5
Heart rate	76 ± 7	77 ± 8	86 ± 9	75 ± 10	88 ± 13
Systemic vascular resistance	0.89 ± 0.09	0.87 ± 0.09	0.89 ± 0.09	0.87 ± 0.09	0.87 ± 0.09
Systolic pulmonary arterial pressure	17.4 ± 1.3	16.7 ± 1.4	16.1 ± 1.5	17.2 ± 1.8	15.2 ± 1.9
Diastolic pulmonary arterial pressure	11.2 ± 0.7	10.8 ± 0.7	10.7 ± 0.8	11.1 ± 1.0	10.1 ± 1.0
Pulmonary vascular resistance	0.100 ± 0.013	0.100 ± 0.013	0.100 ± 0.013	0.100 ± 0.013	0.100 ± 0.013
Stroke volume	70 ± 7	66 ± 8	59 ± 8	69 ± 12	55 ± 11
Ejection fraction	64 ± 7	63 ± 7	60 ± 7	63 ± 7	59 ± 7
Cardiac output	5.3 ± 0.5	5.1 ± 0.5	5.1 ± 0.5	5.1 ± 0.6	4.8 ± 0.5
LV peak systolic pressure	172.4 ± 7.0	156.3 ± 7.9	144.4 ± 8.4	149.1 ± 11.3	138.8 ± 10.1
LV end-diastolic pressure	8.0 ± 1.1	7.7 ± 1.1	7.1 ± 1.1	8.0 ± 1.3	6.7 ± 1.2
LV end-systolic volume	40 ± 11	40 ± 11	39 ± 10	40 ± 11	39 ± 10
LV end-diastolic volume	110 ± 13	106 ± 14	99 ± 14	109 ± 17	95 ± 16
RV peak systolic pressure	19.6 ± 1.4	18.9 ± 1.4	18.3 ± 1.6	19.3 ± 1.9	17.2 ± 2.0
RV end-diastolic pressure	5.9 ± 1.1	5.7 ± 1.1	5.2 ± 1.0	5.9 ± 1.2	4.9 ± 1.1
RV end-systolic volume	47 ± 12	47 ± 12	45 ± 11	48 ± 12	45 ± 11
RV end-diastolic volume	117 ± 14	113 ± 14	105 ± 14	117 ± 18	100 ± 17
Aortic valve peak flow	564 ± 65	535 ± 66	501 ± 64	551 ± 85	469 ± 75
Pulmonary valve peak flow	542 ± 75	516 ± 76	489 ± 75	532 ± 92	459 ± 85
LV early peak filling rate	628 ± 124	605 ± 120	562 ± 113	652 ± 153	516 ± 125
LV active peak filling rate	418 ± 42	361 ± 42	312 ± 38	337 ± 50	299 ± 42
RV early peak filling rate	571 ± 100	562 ± 98	538 ± 96	607 ± 123	498 ± 106
RV active peak filling rate	529 ± 55	461 ± 53	400 ± 48	427 ± 61	386 ± 52
Glomerular filtration rate	0.097 ± 0.018	0.097 ± 0.018	0.106 ± 0.017	0.100 ± 0.019	0.094 ± 0.023
Renal blood flow	1.10 ± 0.15	1.20 ± 0.16	1.30 ± 0.17	1.21 ± 0.16	1.42 ± 0.19
Filtration fraction	0.16 ± 0.04	0.15 ± 0.04	0.15 ± 0.03	0.15 ± 0.04	0.12 ± 0.03
Filtration coefficient	0.010 ± 0.003	0.017 ± 0.005	0.016 ± 0.005	0.037 ± 0.012	0.014 ± 0.004
Renal vascular resistance	105.8 ± 14.2	86.0 ± 12.3	72.1 ± 10.4	77.9 ± 11.7	64.0 ± 9.1
Afferent arteriolar resistance	4842 ± 825	3852 ± 734	2932 ± 608	3464 ± 719	2549 ± 529
Efferent arteriolar resistance	2606 ± 458	1942 ± 348	1739 ± 311	1662 ± 295	1428 ± 255
Macula densa sodium flow rate	2.0 ± 0.8	1.9 ± 0.8	1.9 ± 0.8	1.9 ± 0.8	1.3 ± 0.5
Fractional proximal sodium reabsorption	0.86 ± 0.06	0.86 ± 0.06	0.87 ± 0.06	0.86 ± 0.06	0.89 ± 0.06
Fractional distal sodium reabsorption	0.94 ± 0.03	0.94 ± 0.03	0.94 ± 0.03	0.94 ± 0.03	0.92 ± 0.04
Sodium	143.5 ± 1.3	143.4 ± 1.3	143.4 ± 1.3	143.4 ± 1.3	142.6 ± 1.3
Plasma renin activity	31.8 ± 3.6	217.7 ± 24.9	223.4 ± 25.4	65.4 ± 7.5	324.0 ± 36.7
Plasma renin concentration	33.1 ± 8.1	226.7 ± 55.5	232.6 ± 56.9	68.1 ± 16.7	337.3 ± 81.9
Plasma angiotensin I concentration	8.6 ± 1.0	77.4 ± 8.8	79.4 ± 9.0	23.3 ± 2.7	115.2 ± 13.0
Plasma angiotensin II concentration	5.4 ± 0.6	1.2 ± 0.1	1.2 ± 0.1	0.3 ± 0.04	1.7 ± 0.2
Plasma aldosterone concentration	182.4 ± 56.5	105.4 ± 32.7	106.3 ± 33.0	75.9 ± 23.6	110.5 ± 34.0

Table S9. Correlation coefficients of virtual population parameters with reduction of systolic blood pressure in simulated treatment with aliskiren 150 mg (A150) and 300 mg (A300), amlodipine 5 mg (A5), bisoprolol 5 mg (B5), enalapril 20 mg (E20), HCTZ 12.5 mg (H12.5), losartan 100 mg (L100) and 50 mg (L50), and combinations of these drugs.

Parameters	A150	A150 A5	A150 B5	A150 H12.5	A300	A300 A5	A300 B5	A300 H12.5	A5	B5	E20	E20 A5	E20 B5	E20 H12.5	H12.5	L100	L100 A5	L100 B5	L100 H12.5	L50	L50 A5	L50 B5	L50 H12.5
**General characteristics**																							
Body mass index	-0.11	-0.08	-0.16	-0.02	-0.14	-0.11	-0.16	-0.04	0.01	-0.11	-0.16	-0.14	-0.16	-0.07	0.01	-0.14	-0.13	-0.16	-0.05	-0.11	-0.08	-0.16	-0.02
Total body water	-0.02	-0.05	-0.03	-0.09	-0.02	-0.06	-0.03	-0.09	-0.04	-0.02	-0.03	-0.06	-0.03	-0.10	-0.08	-0.03	-0.06	-0.03	-0.10	-0.02	-0.05	-0.03	-0.09
Sodium intake	-0.13	-0.04	-0.12	0.20	-0.09	-0.02	-0.11	0.20	0.10	-0.23	-0.05	0.00	-0.11	0.20	0.26	-0.07	-0.01	-0.11	0.20	-0.13	-0.04	-0.12	0.20
Water intake	0.02	0.08	0.03	0.00	0.02	0.07	0.03	0.00	0.12	0.01	0.03	0.06	0.03	0.01	0.00	0.02	0.07	0.03	0.01	0.02	0.08	0.02	0.00
Total exchangeable sodium	0.01	0.01	-0.02	0.01	0.00	-0.01	-0.02	0.00	0.03	0.00	-0.01	-0.02	-0.02	-0.01	0.02	-0.01	-0.02	-0.02	0.00	0.01	0.01	-0.02	0.01
Body oxygen demand	-0.02	-0.05	-0.08	0.02	-0.03	-0.05	-0.09	0.02	-0.02	-0.05	-0.05	-0.06	-0.11	0.00	0.02	-0.04	-0.06	-0.10	0.01	-0.02	-0.05	-0.08	0.02
Total metabolic intensity	0.26	0.29	0.24	0.13	0.26	0.30	0.24	0.15	0.09	0.20	0.25	0.28	0.24	0.18	0.06	0.26	0.30	0.24	0.16	0.26	0.29	0.24	0.13
**Systemic hemodynamics**																							
Systolic blood pressure	-0.07	0.07	-0.12	0.10	-0.04	0.06	-0.10	0.10	0.30	-0.28	0.00	0.07	-0.09	0.12	0.16	-0.02	0.06	-0.10	0.11	-0.08	0.07	-0.12	0.10
Diastolic blood pressure	-0.25	-0.38	-0.34	-0.12	-0.31	-0.45	-0.35	-0.18	0.01	-0.13	-0.37	-0.49	-0.37	-0.26	0.02	-0.34	-0.47	-0.36	-0.21	-0.24	-0.38	-0.33	-0.12
Heart rate	0.03	-0.17	0.02	-0.05	0.00	-0.16	0.00	-0.06	-0.23	0.12	-0.03	-0.15	-0.02	-0.07	-0.04	-0.01	-0.16	-0.01	-0.06	0.03	-0.17	0.02	-0.05
Systemic arterial tone	0.20	0.22	0.18	0.13	0.18	0.20	0.17	0.13	0.13	0.21	0.16	0.17	0.16	0.14	0.08	0.18	0.19	0.16	0.14	0.20	0.23	0.18	0.13
Sympathetic sensitivity of microvessels	-0.16	0.08	-0.10	0.11	-0.08	0.12	-0.06	0.14	0.18	-0.38	0.01	0.18	-0.02	0.19	0.14	-0.04	0.15	-0.04	0.16	-0.17	0.08	-0.10	0.11
Basic systemic arterial elasticity	0.07	0.22	0.09	0.10	0.13	0.27	0.11	0.14	0.14	-0.16	0.19	0.30	0.13	0.20	0.06	0.16	0.28	0.12	0.16	0.06	0.22	0.09	0.09
Basic systemic venous elasticity	0.03	0.10	0.05	0.17	0.05	0.10	0.05	0.18	0.12	-0.02	0.07	0.11	0.06	0.19	0.17	0.06	0.10	0.06	0.18	0.03	0.10	0.05	0.17
Systemic vascular resistance	-0.09	0.02	-0.04	-0.02	-0.05	0.04	-0.02	0.00	0.11	-0.19	0.00	0.06	0.00	0.02	0.01	-0.03	0.05	-0.01	0.01	-0.09	0.02	-0.04	-0.02
**Pulmonary hemodynamics**																							
Systolic blood pressure	0.01	-0.02	0.02	-0.09	0.00	-0.03	0.02	-0.10	0.00	0.06	-0.01	-0.04	0.03	-0.10	-0.09	0.00	-0.04	0.03	-0.10	0.01	-0.02	0.02	-0.09
Diastolic blood pressure	0.00	-0.03	0.00	-0.03	-0.01	-0.04	0.00	-0.04	0.00	0.03	-0.02	-0.06	0.00	-0.05	-0.02	-0.01	-0.05	0.00	-0.05	0.00	-0.02	0.00	-0.03
Basic pulmonary arterial elasticity	-0.02	-0.06	0.01	-0.10	-0.02	-0.05	0.01	-0.11	-0.06	0.02	-0.02	-0.05	0.02	-0.11	-0.10	-0.02	-0.05	0.02	-0.11	-0.02	-0.06	0.01	-0.10
Basic pulmonary venous elasticity	0.08	0.00	0.09	0.05	0.06	0.00	0.08	0.04	-0.03	0.12	0.05	0.00	0.08	0.04	0.06	0.06	0.00	0.08	0.04	0.08	0.00	0.09	0.05
Pulmonary vascular resistance	-0.02	-0.01	0.03	-0.09	0.01	0.01	0.04	-0.08	0.00	-0.05	0.03	0.03	0.06	-0.07	-0.09	0.02	0.02	0.05	-0.08	-0.02	-0.01	0.03	-0.09
**Left ventricle (LV)**																							
Stroke volume	-0.08	0.02	-0.11	-0.02	-0.08	-0.01	-0.10	-0.03	0.15	-0.09	-0.09	-0.04	-0.10	-0.04	0.01	-0.09	-0.02	-0.10	-0.04	-0.08	0.02	-0.11	-0.02
Ejection fraction	-0.02	0.00	-0.01	0.00	-0.01	0.00	-0.01	0.00	0.02	-0.04	0.00	0.00	-0.01	-0.01	0.00	-0.01	0.00	-0.01	0.00	-0.02	0.00	-0.01	0.00
Cardiac output	-0.06	-0.18	-0.10	-0.07	-0.10	-0.20	-0.11	-0.09	-0.10	0.04	-0.13	-0.21	-0.12	-0.13	-0.03	-0.11	-0.21	-0.12	-0.11	-0.06	-0.17	-0.10	-0.07
LV peak systolic pressure	-0.06	0.01	-0.11	0.08	-0.04	0.00	-0.10	0.07	0.22	-0.20	-0.03	0.00	-0.10	0.08	0.14	-0.04	-0.01	-0.10	0.07	-0.06	0.01	-0.11	0.08
LV end-diastolic pressure	0.00	0.05	0.02	0.02	0.01	0.04	0.02	0.02	0.04	0.00	0.01	0.03	0.03	0.02	0.02	0.01	0.04	0.03	0.02	0.00	0.05	0.02	0.02
LV end-systolic volume	-0.01	0.00	-0.02	-0.01	-0.02	-0.01	-0.02	-0.01	0.03	0.01	-0.03	-0.02	-0.03	-0.02	-0.01	-0.02	-0.01	-0.03	-0.02	-0.01	0.00	-0.02	-0.01
LV end-diastolic volume	-0.04	0.01	-0.07	-0.02	-0.06	-0.01	-0.07	-0.03	0.10	-0.04	-0.07	-0.03	-0.07	-0.04	0.00	-0.06	-0.02	-0.07	-0.03	-0.04	0.01	-0.07	-0.02
LV wall elasticity	0.02	0.03	0.03	-0.01	0.03	0.03	0.03	-0.01	-0.03	0.00	0.04	0.04	0.03	0.00	-0.03	0.03	0.04	0.03	0.00	0.02	0.03	0.03	-0.01
LV inotropic state	0.03	0.06	0.03	0.07	0.04	0.07	0.04	0.08	0.00	-0.01	0.05	0.08	0.04	0.10	0.06	0.04	0.08	0.04	0.09	0.03	0.06	0.03	0.07
**Right ventricle (RV)**																							
RV peak systolic pressure	0.02	-0.02	0.03	-0.08	0.01	-0.04	0.02	-0.09	-0.01	0.07	-0.01	-0.05	0.03	-0.10	-0.08	0.00	-0.04	0.03	-0.09	0.02	-0.02	0.03	-0.08
RV end-diastolic pressure	0.06	0.08	0.04	0.00	0.06	0.09	0.04	0.01	0.02	0.01	0.07	0.10	0.04	0.03	-0.03	0.06	0.09	0.04	0.02	0.05	0.08	0.04	0.00
RV end-systolic volume	0.01	0.02	0.01	0.06	0.01	0.02	0.01	0.06	0.02	0.01	0.01	0.03	0.02	0.06	0.06	0.01	0.02	0.02	0.06	0.01	0.02	0.01	0.06
RV end-diastolic volume	-0.03	0.03	-0.04	0.04	-0.03	0.01	-0.04	0.04	0.09	-0.03	-0.03	0.00	-0.03	0.03	0.05	-0.03	0.01	-0.03	0.03	-0.03	0.03	-0.04	0.04
RV wall elasticity	0.03	0.02	0.03	-0.06	0.03	0.04	0.03	-0.05	-0.03	0.00	0.04	0.05	0.03	-0.04	-0.07	0.04	0.04	0.03	-0.05	0.03	0.02	0.03	-0.06
RV inotropic state	0.04	0.10	0.01	-0.02	0.04	0.09	0.01	-0.02	0.08	0.02	0.03	0.06	0.00	-0.02	-0.05	0.04	0.08	0.00	-0.02	0.04	0.10	0.01	-0.02

Parameters	A150	A150 A5	A150 B5	A150 H12.5	A300	A300 A5	A300 B5	A300 H12.5	A5	B5	E20	E20 A5	E20 B5	E20 H12.5	H12.5	L100	L100 A5	L100 B5	L100 H12.5	L50	L50 A5	L50 B5	L50 H12.5
**Peak flow rates through heart valves**																							
Aortic valve peak flow	0.02	0.19	0.02	0.08	0.07	0.21	0.04	0.11	0.18	-0.18	0.12	0.23	0.05	0.15	0.06	0.09	0.22	0.05	0.13	0.02	0.19	0.02	0.08
Pulmonary valve peak flow	-0.05	-0.06	-0.06	-0.12	-0.07	-0.08	-0.06	-0.13	-0.01	0.00	-0.08	-0.10	-0.05	-0.15	-0.11	-0.07	-0.09	-0.05	-0.14	-0.05	-0.06	-0.06	-0.12
LV early peak filling rate	0.04	0.03	0.06	0.00	0.04	0.03	0.06	0.00	-0.03	0.05	0.04	0.03	0.06	0.00	-0.02	0.04	0.03	0.06	0.00	0.04	0.03	0.06	0.00
LV active peak filling rate	-0.23	-0.16	-0.28	-0.06	-0.25	-0.22	-0.28	-0.09	0.14	-0.22	-0.28	-0.26	-0.28	-0.14	0.04	-0.26	-0.24	-0.28	-0.11	-0.23	-0.16	-0.28	-0.05
RV early peak filling rate	0.05	0.06	0.05	0.02	0.06	0.07	0.06	0.02	0.00	0.02	0.07	0.08	0.05	0.04	0.00	0.07	0.08	0.05	0.03	0.05	0.06	0.05	0.02
RV active peak filling rate	-0.20	-0.14	-0.28	-0.05	-0.23	-0.19	-0.28	-0.08	0.14	-0.22	-0.25	-0.23	-0.29	-0.12	0.03	-0.24	-0.21	-0.28	-0.10	-0.20	-0.13	-0.28	-0.05
**Renal hemodynamics**																							
Glomerular filtration rate	0.26	0.47	0.24	0.05	0.26	0.43	0.23	0.07	0.45	0.20	0.25	0.37	0.22	0.11	-0.01	0.26	0.41	0.23	0.09	0.26	0.47	0.24	0.05
Renal blood flow	0.32	0.14	0.35	-0.03	0.32	0.17	0.35	-0.01	-0.10	0.29	0.32	0.21	0.36	0.03	-0.09	0.32	0.19	0.35	0.00	0.31	0.13	0.35	-0.03
Filtration fraction	0.06	0.32	-0.01	0.11	0.04	0.25	-0.02	0.11	0.44	0.03	0.03	0.17	-0.04	0.11	0.09	0.04	0.22	-0.03	0.11	0.06	0.32	-0.01	0.11
Filtration coefficient	-0.45	-0.03	-0.59	0.05	-0.50	-0.16	-0.60	0.00	0.43	-0.42	-0.55	-0.29	-0.60	-0.08	0.12	-0.52	-0.21	-0.60	-0.03	-0.45	-0.02	-0.59	0.05
Renal vascular resistance	-0.40	-0.22	-0.45	0.02	-0.41	-0.28	-0.46	-0.01	0.16	-0.38	-0.41	-0.32	-0.47	-0.07	0.12	-0.41	-0.29	-0.46	-0.03	-0.39	-0.22	-0.45	0.02
Number of nephrons	0.03	0.02	0.00	-0.01	0.03	0.03	-0.01	0.00	0.02	-0.01	0.03	0.03	-0.01	0.01	-0.02	0.03	0.03	-0.01	0.00	0.03	0.02	0.00	-0.01
Afferent arteriole diameter	0.55	0.52	0.64	0.42	0.59	0.57	0.65	0.47	0.07	0.49	0.62	0.59	0.66	0.53	0.29	0.61	0.58	0.65	0.49	0.55	0.52	0.64	0.42
Efferent arteriole diameter	0.17	0.16	0.17	0.06	0.16	0.15	0.17	0.07	0.02	0.19	0.14	0.14	0.17	0.08	0.00	0.15	0.15	0.17	0.07	0.17	0.16	0.17	0.06
Afferent arteriole length	0.00	0.00	-0.02	0.05	-0.01	-0.01	-0.02	0.04	0.04	0.03	-0.03	-0.03	-0.02	0.02	0.05	-0.02	-0.02	-0.02	0.03	0.00	0.01	-0.02	0.05
Efferent arteriole length	-0.06	-0.01	-0.07	0.04	-0.05	0.00	-0.07	0.04	0.05	-0.11	-0.03	0.00	-0.07	0.05	0.05	-0.04	0.00	-0.07	0.04	-0.06	-0.01	-0.07	0.04
Afferent arteriolar resistance	-0.46	-0.24	-0.55	0.01	-0.49	-0.31	-0.56	-0.03	0.22	-0.44	-0.51	-0.38	-0.57	-0.10	0.13	-0.50	-0.34	-0.56	-0.06	-0.46	-0.23	-0.55	0.01
Efferent arteriolar resistance	-0.22	-0.18	-0.19	-0.03	-0.20	-0.18	-0.18	-0.04	0.00	-0.22	-0.18	-0.16	-0.19	-0.06	0.05	-0.19	-0.17	-0.18	-0.05	-0.22	-0.18	-0.19	-0.03
Renal venous resistance	0.14	0.19	0.11	0.15	0.14	0.18	0.10	0.16	0.12	0.10	0.14	0.16	0.09	0.17	0.10	0.14	0.17	0.10	0.17	0.14	0.19	0.11	0.15
Renal venous pressure	0.04	0.03	0.00	-0.03	0.01	0.00	-0.01	-0.04	0.04	0.09	-0.02	-0.03	-0.02	-0.06	-0.03	0.00	-0.01	-0.01	-0.05	0.04	0.03	0.00	-0.03
Glomerular hydrostatic pressure	0.35	0.04	0.44	-0.02	0.38	0.11	0.44	0.01	-0.21	0.31	0.41	0.19	0.45	0.05	-0.04	0.40	0.14	0.45	0.02	0.35	0.03	0.44	-0.02
Glomerular capillary oncotic pressure	-0.22	-0.21	-0.28	-0.04	-0.23	-0.25	-0.28	-0.07	0.00	-0.22	-0.25	-0.28	-0.28	-0.11	0.03	-0.24	-0.26	-0.28	-0.08	-0.21	-0.21	-0.28	-0.04
Bowman's space hydrostatic pressure	-0.14	-0.15	-0.13	0.08	-0.14	-0.16	-0.13	0.05	-0.04	-0.09	-0.15	-0.17	-0.13	0.02	0.12	-0.15	-0.17	-0.13	0.04	-0.14	-0.15	-0.13	0.08
Macula densa sodium flow rate	0.08	0.43	0.05	0.76	0.09	0.35	0.04	0.75	0.62	0.01	0.09	0.25	0.03	0.71	0.79	0.09	0.31	0.04	0.73	0.08	0.43	0.05	0.76
Fractional proximal sodium reabsorption	0.04	-0.23	0.06	-0.74	0.03	-0.17	0.06	-0.71	-0.44	0.08	0.03	-0.09	0.07	-0.65	-0.79	0.03	-0.14	0.06	-0.69	0.04	-0.24	0.06	-0.74
Fractional distal sodium reabsorption	0.22	0.53	0.18	0.61	0.20	0.44	0.17	0.59	0.62	0.22	0.17	0.31	0.16	0.56	0.58	0.19	0.39	0.16	0.58	0.22	0.54	0.18	0.61
**Blood parameters**																							
Hematocrit	0.08	0.06	-0.01	0.10	0.05	0.03	-0.03	0.09	0.05	0.08	0.01	0.00	-0.04	0.07	0.08	0.04	0.02	-0.03	0.09	0.08	0.06	-0.01	0.10
Hemoglobin	-0.10	-0.09	-0.07	-0.11	-0.10	-0.09	-0.07	-0.12	-0.01	-0.06	-0.10	-0.09	-0.05	-0.12	-0.09	-0.10	-0.09	-0.06	-0.12	-0.10	-0.08	-0.07	-0.11
Sodium	0.14	0.33	0.07	0.56	0.13	0.27	0.05	0.55	0.40	0.11	0.10	0.18	0.03	0.51	0.56	0.12	0.23	0.05	0.54	0.14	0.33	0.07	0.56
Potassium	-0.08	0.00	-0.11	0.04	-0.09	-0.03	-0.11	0.03	0.08	-0.07	-0.10	-0.06	-0.12	0.01	0.05	-0.10	-0.04	-0.12	0.02	-0.08	0.00	-0.11	0.04
Glucose	0.08	0.12	0.10	0.00	0.10	0.13	0.11	0.02	0.01	0.04	0.11	0.14	0.11	0.05	-0.03	0.11	0.14	0.11	0.03	0.08	0.11	0.10	0.00
Total protein	-0.21	-0.29	-0.25	-0.06	-0.22	-0.30	-0.24	-0.09	-0.13	-0.20	-0.23	-0.30	-0.24	-0.13	0.00	-0.23	-0.30	-0.24	-0.11	-0.21	-0.29	-0.25	-0.06
Urea	-0.19	-0.10	-0.11	-0.05	-0.16	-0.09	-0.09	-0.05	0.05	-0.18	-0.12	-0.08	-0.08	-0.05	0.01	-0.14	-0.09	-0.08	-0.05	-0.19	-0.10	-0.11	-0.04
Blood viscosity	0.08	0.07	-0.02	0.11	0.05	0.04	-0.04	0.10	0.06	0.08	0.01	-0.01	-0.06	0.08	0.09	0.03	0.02	-0.04	0.09	0.08	0.07	-0.02	0.11
Arterial oxygen content	-0.10	-0.07	-0.07	-0.08	-0.10	-0.07	-0.06	-0.08	0.01	-0.08	-0.09	-0.06	-0.04	-0.08	-0.06	-0.09	-0.07	-0.05	-0.08	-0.10	-0.07	-0.07	-0.08
Venous oxygen content	-0.09	-0.07	-0.03	-0.10	-0.09	-0.08	-0.01	-0.11	0.00	-0.02	-0.08	-0.07	0.01	-0.11	-0.08	-0.09	-0.08	-0.01	-0.11	-0.09	-0.07	-0.03	-0.10
Arterial oxygen saturation	-0.06	-0.03	-0.06	0.05	-0.04	-0.01	-0.05	0.05	0.01	-0.14	-0.02	0.01	-0.05	0.06	0.06	-0.03	0.00	-0.05	0.06	-0.06	-0.03	-0.06	0.05
Oxygen capacity of hemoglobin	0.07	0.14	0.11	0.12	0.09	0.15	0.11	0.13	0.09	0.05	0.11	0.16	0.12	0.15	0.09	0.09	0.16	0.11	0.14	0.07	0.14	0.11	0.12

Parameters	A150	A150 A5	A150 B5	A150 H12.5	A300	A300 A5	A300 B5	A300 H12.5	A5	B5	E20	E20 A5	E20 B5	E20 H12.5	H12.5	L100	L100 A5	L100 B5	L100 H12.5	L50	L50 A5	L50 B5	L50 H12.5
**Renin–angiotensin–aldosterone system**																							
Plasma renin activity	-0.11	-0.13	0.15	-0.17	-0.02	-0.04	0.19	-0.14	-0.10	-0.10	0.09	0.06	0.22	-0.08	-0.12	0.03	0.00	0.21	-0.12	-0.12	-0.13	0.15	-0.17
Plasma renin concentration	-0.11	-0.17	-0.03	-0.15	-0.09	-0.14	-0.02	-0.15	-0.17	-0.07	-0.06	-0.09	-0.01	-0.14	-0.13	-0.07	-0.12	-0.02	-0.15	-0.11	-0.18	-0.03	-0.15
Plasma angiotensin I concentration	-0.11	-0.13	0.15	-0.17	-0.02	-0.04	0.19	-0.14	-0.10	-0.10	0.09	0.06	0.22	-0.08	-0.12	0.03	0.00	0.21	-0.12	-0.12	-0.13	0.15	-0.17
Plasma angiotensin II concentration	-0.11	-0.13	0.15	-0.17	-0.02	-0.04	0.19	-0.14	-0.10	-0.10	0.09	0.06	0.22	-0.08	-0.12	0.03	0.00	0.21	-0.12	-0.12	-0.13	0.15	-0.17
Plasma aldosterone concentration	0.02	0.10	0.01	0.12	0.01	0.07	0.01	0.11	0.15	0.04	0.00	0.04	0.01	0.10	0.11	0.00	0.06	0.01	0.11	0.02	0.10	0.01	0.12
**Hormones**																							
Plasma antidiuretic hormone	0.07	0.39	0.04	0.69	0.08	0.31	0.04	0.68	0.56	0.01	0.08	0.22	0.02	0.64	0.72	0.08	0.28	0.03	0.66	0.07	0.39	0.04	0.69
Plasma atrial natriuretic peptide	0.15	0.06	0.12	0.00	0.14	0.07	0.11	0.01	-0.08	0.15	0.12	0.06	0.10	0.02	-0.04	0.13	0.07	0.11	0.01	0.15	0.06	0.12	0.00

Table S10. Correlation coefficients of virtual population parameters with reduction of diastolic blood pressure in simulated treatment with aliskiren 150 mg (A150) and 300 mg (A300), amlodipine 5 mg (A5), bisoprolol 5 mg (B5), enalapril 20 mg (E20), HCTZ 12.5 mg (H12.5), losartan 100 mg (L100) and 50 mg (L50), and combinations of these drugs.

Parameters	A150	A150 A5	A150 B5	A150 H12.5	A300	A300 A5	A300 B5	A300 H12.5	A5	B5	E20	E20 A5	E20 B5	E20 H12.5	H12.5	L100	L100 A5	L100 B5	L100 H12.5	L50	L50 A5	L50 B5	L50 H12.5
**General characteristics**																							
Body mass index	-0.10	-0.10	-0.10	0.00	-0.10	-0.09	-0.10	0.00	-0.01	-0.06	-0.10	-0.08	-0.11	0.00	0.06	-0.10	-0.09	-0.10	0.00	-0.10	-0.10	-0.10	0.00
Total body water	-0.04	-0.03	-0.05	-0.06	-0.05	-0.03	-0.06	-0.05	0.02	-0.03	-0.04	-0.03	-0.05	-0.05	-0.02	-0.05	-0.03	-0.05	-0.05	-0.04	-0.03	-0.05	-0.06
Sodium intake	0.01	0.00	-0.01	0.15	-0.04	-0.06	-0.04	0.10	0.08	0.14	-0.09	-0.14	-0.07	0.03	0.20	-0.06	-0.09	-0.06	0.08	0.01	0.01	-0.01	0.16
Water intake	0.12	0.18	0.14	0.09	0.13	0.18	0.14	0.09	0.12	0.10	0.13	0.16	0.14	0.09	0.05	0.13	0.17	0.14	0.09	0.12	0.18	0.14	0.09
Total exchangeable sodium	-0.06	-0.01	-0.06	0.01	-0.06	-0.02	-0.06	0.01	0.08	-0.04	-0.06	-0.02	-0.05	0.00	0.06	-0.06	-0.02	-0.06	0.01	-0.06	-0.01	-0.06	0.01
Body oxygen demand	-0.11	0.04	-0.14	-0.06	-0.13	-0.01	-0.15	-0.08	0.21	-0.07	-0.14	-0.05	-0.13	-0.10	0.01	-0.13	-0.03	-0.14	-0.09	-0.11	0.04	-0.14	-0.06
Total metabolic intensity	-0.01	-0.07	0.03	-0.03	-0.01	-0.05	0.04	-0.02	-0.11	0.02	0.01	-0.02	0.05	0.00	-0.04	0.00	-0.04	0.04	-0.01	-0.01	-0.07	0.03	-0.03
**Systemic hemodynamics**																							
Systolic blood pressure	0.34	0.38	0.35	0.23	0.28	0.28	0.31	0.17	0.37	0.49	0.21	0.14	0.27	0.09	0.19	0.25	0.23	0.29	0.14	0.34	0.38	0.35	0.23
Diastolic blood pressure	0.23	0.48	0.15	0.33	0.25	0.44	0.15	0.32	0.60	0.05	0.25	0.37	0.14	0.30	0.35	0.25	0.41	0.15	0.31	0.23	0.48	0.15	0.33
Heart rate	-0.06	0.04	-0.06	0.04	-0.03	0.05	-0.03	0.05	0.09	-0.15	0.01	0.08	0.01	0.07	0.07	-0.02	0.06	-0.01	0.06	-0.06	0.04	-0.06	0.04
Systemic arterial tone	0.02	0.11	0.07	0.10	0.05	0.13	0.09	0.12	0.11	-0.02	0.09	0.16	0.12	0.15	0.09	0.06	0.14	0.10	0.13	0.02	0.11	0.07	0.10
Sympathetic sensitivity of microvessels	0.16	-0.21	0.16	-0.03	0.07	-0.28	0.10	-0.10	-0.29	0.44	-0.04	-0.37	0.02	-0.20	-0.03	0.03	-0.32	0.06	-0.14	0.16	-0.20	0.17	-0.03
Basic systemic arterial elasticity	0.10	-0.03	0.16	-0.03	0.06	-0.07	0.14	-0.06	-0.11	0.33	0.00	-0.11	0.12	-0.10	-0.06	0.03	-0.08	0.13	-0.07	0.10	-0.03	0.17	-0.02
Basic systemic venous elasticity	0.04	0.00	0.03	0.11	0.03	-0.01	0.01	0.09	-0.02	0.09	0.01	-0.04	-0.02	0.07	0.11	0.02	-0.02	0.00	0.08	0.04	0.00	0.03	0.11
Systemic vascular resistance	0.28	0.14	0.29	0.12	0.25	0.13	0.26	0.10	-0.04	0.35	0.21	0.09	0.21	0.07	0.02	0.24	0.11	0.24	0.09	0.28	0.14	0.29	0.12
**Pulmonary hemodynamics**																							
Systolic blood pressure	0.08	0.05	0.06	-0.05	0.10	0.07	0.06	-0.03	-0.03	0.01	0.11	0.09	0.04	0.00	-0.10	0.10	0.08	0.05	-0.02	0.08	0.05	0.06	-0.05
Diastolic blood pressure	0.04	0.09	0.03	0.06	0.05	0.10	0.04	0.08	0.08	0.00	0.07	0.11	0.04	0.09	0.05	0.06	0.11	0.04	0.08	0.04	0.09	0.03	0.06
Basic pulmonary arterial elasticity	0.07	-0.03	0.05	-0.08	0.08	0.00	0.05	-0.06	-0.14	0.01	0.09	0.03	0.03	-0.03	-0.13	0.08	0.01	0.04	-0.04	0.07	-0.04	0.05	-0.08
Basic pulmonary venous elasticity	0.07	0.08	0.09	0.11	0.09	0.11	0.10	0.13	0.01	0.01	0.12	0.13	0.11	0.15	0.10	0.10	0.12	0.11	0.14	0.07	0.08	0.09	0.11
Pulmonary vascular resistance	0.22	0.08	0.20	-0.01	0.21	0.09	0.18	0.00	-0.13	0.21	0.19	0.09	0.15	0.01	-0.12	0.21	0.09	0.17	0.00	0.22	0.08	0.20	-0.01
**Left ventricle (LV)**																							
Stroke volume	-0.06	-0.04	-0.06	-0.05	-0.07	-0.06	-0.08	-0.06	0.06	-0.01	-0.08	-0.08	-0.09	-0.08	-0.01	-0.07	-0.07	-0.08	-0.07	-0.06	-0.04	-0.06	-0.05
Ejection fraction	0.01	-0.06	0.01	-0.03	0.01	-0.05	0.01	-0.03	-0.09	0.04	0.00	-0.05	-0.01	-0.03	-0.05	0.00	-0.05	0.00	-0.03	0.01	-0.06	0.01	-0.03
Cardiac output	-0.14	0.00	-0.14	0.00	-0.12	0.00	-0.13	0.00	0.16	-0.19	-0.09	0.00	-0.10	0.00	0.08	-0.11	0.00	-0.12	0.00	-0.14	0.00	-0.14	0.00
LV peak systolic pressure	0.29	0.38	0.29	0.21	0.25	0.29	0.26	0.17	0.40	0.37	0.19	0.16	0.24	0.09	0.19	0.23	0.24	0.25	0.14	0.29	0.38	0.29	0.22
LV end-diastolic pressure	-0.01	-0.03	-0.01	0.01	-0.01	-0.02	-0.01	0.02	-0.05	0.00	0.00	-0.01	-0.01	0.03	0.01	-0.01	-0.01	-0.01	0.02	-0.01	-0.03	0.00	0.01
LV end-systolic volume	-0.03	0.05	-0.04	0.01	-0.03	0.04	-0.04	0.01	0.12	-0.05	-0.03	0.02	-0.03	0.00	0.04	-0.03	0.03	-0.03	0.01	-0.03	0.05	-0.04	0.01
LV end-diastolic volume	-0.06	0.02	-0.07	-0.02	-0.06	0.00	-0.07	-0.02	0.13	-0.05	-0.07	-0.02	-0.07	-0.04	0.03	-0.06	-0.01	-0.07	-0.03	-0.06	0.02	-0.07	-0.02
LV wall elasticity	-0.02	-0.07	-0.01	-0.03	-0.02	-0.06	-0.01	-0.03	-0.09	0.01	-0.02	-0.04	-0.01	-0.02	-0.03	-0.02	-0.05	-0.01	-0.03	-0.02	-0.07	-0.01	-0.03
LV inotropic state	-0.07	-0.16	-0.07	-0.04	-0.08	-0.15	-0.07	-0.04	-0.17	-0.02	-0.09	-0.15	-0.08	-0.05	-0.03	-0.08	-0.15	-0.08	-0.05	-0.07	-0.16	-0.07	-0.04
**Right ventricle (RV)**																							
RV peak systolic pressure	0.06	0.06	0.04	-0.05	0.08	0.08	0.04	-0.03	0.01	-0.02	0.09	0.09	0.03	0.00	-0.09	0.08	0.08	0.04	-0.02	0.06	0.06	0.04	-0.05
RV end-diastolic pressure	0.00	-0.02	-0.02	-0.10	-0.02	-0.03	-0.02	-0.11	0.00	0.02	-0.04	-0.05	-0.02	-0.11	-0.10	-0.03	-0.04	-0.02	-0.11	0.00	-0.01	-0.02	-0.10
RV end-systolic volume	-0.04	-0.03	-0.04	0.01	-0.04	-0.04	-0.04	0.00	0.00	-0.03	-0.04	-0.04	-0.03	-0.01	0.03	-0.04	-0.04	-0.04	0.00	-0.04	-0.03	-0.04	0.01
RV end-diastolic volume	-0.06	-0.04	-0.06	-0.02	-0.07	-0.06	-0.07	-0.03	0.03	-0.04	-0.08	-0.07	-0.07	-0.04	0.02	-0.07	-0.06	-0.07	-0.03	-0.06	-0.04	-0.06	-0.02
RV wall elasticity	-0.01	-0.04	-0.02	-0.08	-0.01	-0.04	-0.02	-0.08	-0.06	0.01	-0.02	-0.04	-0.03	-0.07	-0.09	-0.02	-0.04	-0.02	-0.08	-0.01	-0.04	-0.02	-0.08
RV inotropic state	-0.06	-0.01	-0.06	-0.06	-0.06	-0.02	-0.06	-0.06	0.04	-0.04	-0.06	-0.02	-0.07	-0.05	-0.04	-0.06	-0.02	-0.06	-0.05	-0.06	-0.01	-0.06	-0.06

Parameters	A150	A150 A5	A150 B5	A150 H12.5	A300	A300 A5	A300 B5	A300 H12.5	A5	B5	E20	E20 A5	E20 B5	E20 H12.5	H12.5	L100	L100 A5	L100 B5	L100 H12.5	L50	L50 A5	L50 B5	L50 H12.5
**Peak flow rates through heart valves**																							
Aortic valve peak flow	0.02	-0.08	0.08	-0.05	-0.03	-0.12	0.05	-0.09	-0.08	0.25	-0.08	-0.18	0.03	-0.14	-0.05	-0.05	-0.14	0.04	-0.11	0.02	-0.08	0.08	-0.05
Pulmonary valve peak flow	-0.01	-0.05	-0.03	-0.10	0.00	-0.03	-0.03	-0.08	-0.05	-0.05	0.01	-0.01	-0.05	-0.07	-0.10	0.00	-0.02	-0.04	-0.08	-0.01	-0.05	-0.03	-0.10
LV early peak filling rate	-0.06	-0.10	-0.04	-0.05	-0.05	-0.08	-0.04	-0.04	-0.09	-0.04	-0.04	-0.05	-0.03	-0.03	-0.03	-0.05	-0.06	-0.03	-0.04	-0.06	-0.10	-0.04	-0.05
LV active peak filling rate	0.05	0.15	0.02	0.16	0.05	0.12	0.01	0.15	0.26	0.06	0.04	0.07	-0.02	0.12	0.21	0.04	0.10	0.00	0.14	0.05	0.15	0.02	0.16
RV early peak filling rate	-0.08	-0.08	-0.09	-0.11	-0.09	-0.10	-0.10	-0.12	-0.03	-0.04	-0.10	-0.11	-0.10	-0.13	-0.08	-0.10	-0.10	-0.10	-0.12	-0.08	-0.08	-0.09	-0.11
RV active peak filling rate	0.04	0.18	0.00	0.13	0.03	0.13	-0.02	0.10	0.33	0.07	0.01	0.07	-0.04	0.06	0.18	0.02	0.10	-0.02	0.09	0.04	0.18	0.00	0.13
**Renal hemodynamics**																							
Glomerular filtration rate	0.24	0.45	0.28	0.11	0.25	0.43	0.29	0.13	0.42	0.19	0.26	0.37	0.30	0.15	0.02	0.26	0.41	0.29	0.14	0.24	0.45	0.27	0.11
Renal blood flow	0.34	0.26	0.36	0.13	0.37	0.30	0.38	0.16	-0.02	0.23	0.39	0.32	0.39	0.19	-0.01	0.38	0.31	0.38	0.18	0.34	0.26	0.36	0.13
Filtration fraction	-0.05	0.22	-0.04	0.04	-0.05	0.17	-0.03	0.03	0.42	-0.03	-0.05	0.11	-0.01	0.02	0.07	-0.05	0.15	-0.03	0.03	-0.05	0.23	-0.04	0.04
Filtration coefficient	-0.39	-0.08	-0.50	-0.04	-0.42	-0.16	-0.53	-0.08	0.34	-0.31	-0.46	-0.25	-0.56	-0.13	0.11	-0.44	-0.20	-0.54	-0.09	-0.38	-0.08	-0.50	-0.04
Renal vascular resistance	-0.21	-0.07	-0.25	0.01	-0.24	-0.14	-0.27	-0.04	0.23	-0.12	-0.28	-0.20	-0.29	-0.09	0.14	-0.26	-0.17	-0.28	-0.06	-0.21	-0.07	-0.25	0.01
Number of nephrons	0.10	0.10	0.06	0.06	0.08	0.08	0.05	0.05	0.07	0.09	0.05	0.04	0.04	0.03	0.01	0.07	0.06	0.04	0.04	0.10	0.10	0.06	0.06
Afferent arteriole diameter	0.21	0.04	0.29	0.16	0.24	0.11	0.31	0.19	-0.28	0.18	0.28	0.18	0.34	0.24	0.04	0.25	0.14	0.33	0.21	0.21	0.04	0.29	0.16
Efferent arteriole diameter	0.09	0.13	0.07	0.08	0.10	0.16	0.08	0.10	0.04	0.00	0.13	0.18	0.08	0.12	0.02	0.11	0.17	0.08	0.11	0.09	0.13	0.07	0.08
Afferent arteriole length	-0.02	0.01	-0.04	0.01	-0.01	0.01	-0.04	0.01	0.05	-0.05	-0.01	0.00	-0.05	0.01	0.02	-0.01	0.00	-0.05	0.01	-0.02	0.01	-0.04	0.01
Efferent arteriole length	-0.03	-0.08	-0.02	-0.04	-0.05	-0.10	-0.02	-0.06	-0.02	0.05	-0.08	-0.13	-0.02	-0.09	-0.01	-0.06	-0.11	-0.02	-0.08	-0.03	-0.08	-0.02	-0.04
Afferent arteriolar resistance	-0.23	-0.01	-0.28	0.05	-0.26	-0.09	-0.31	0.00	0.34	-0.14	-0.30	-0.18	-0.33	-0.06	0.19	-0.28	-0.13	-0.32	-0.02	-0.22	0.00	-0.28	0.05
Efferent arteriolar resistance	-0.15	-0.18	-0.11	-0.13	-0.16	-0.19	-0.11	-0.16	-0.04	-0.05	-0.17	-0.20	-0.11	-0.17	-0.04	-0.16	-0.20	-0.11	-0.17	-0.14	-0.17	-0.11	-0.13
Renal venous resistance	0.04	0.08	0.00	0.11	0.03	0.06	0.00	0.10	0.08	0.03	0.01	0.03	-0.01	0.08	0.09	0.02	0.04	-0.01	0.09	0.04	0.08	0.00	0.11
Renal venous pressure	-0.03	0.08	-0.07	0.03	-0.02	0.08	-0.07	0.05	0.13	-0.11	0.00	0.09	-0.07	0.06	0.04	-0.01	0.09	-0.07	0.05	-0.03	0.08	-0.07	0.03
Glomerular hydrostatic pressure	0.42	0.23	0.48	0.09	0.44	0.27	0.49	0.12	-0.10	0.33	0.47	0.29	0.50	0.14	-0.04	0.45	0.28	0.50	0.13	0.42	0.22	0.48	0.09
Glomerular capillary oncotic pressure	-0.12	-0.12	-0.18	-0.04	-0.14	-0.15	-0.20	-0.06	0.02	-0.07	-0.17	-0.19	-0.23	-0.10	0.04	-0.15	-0.17	-0.21	-0.08	-0.12	-0.12	-0.18	-0.04
Bowman's space hydrostatic pressure	-0.04	-0.04	-0.12	0.04	-0.05	-0.05	-0.14	0.03	-0.01	-0.07	-0.06	-0.06	-0.16	0.02	0.06	-0.05	-0.05	-0.14	0.02	-0.04	-0.04	-0.12	0.04
Macula densa sodium flow rate	0.07	0.30	0.12	0.61	0.07	0.25	0.12	0.56	0.45	0.14	0.06	0.17	0.12	0.51	0.67	0.06	0.22	0.12	0.54	0.07	0.31	0.12	0.61
Fractional proximal sodium reabsorption	0.03	-0.13	-0.01	-0.57	0.04	-0.08	0.00	-0.52	-0.28	-0.06	0.05	-0.03	0.01	-0.45	-0.67	0.04	-0.06	0.00	-0.49	0.03	-0.13	-0.01	-0.57
Fractional distal sodium reabsorption	0.09	0.36	0.16	0.49	0.11	0.34	0.18	0.49	0.44	0.05	0.15	0.32	0.21	0.48	0.52	0.13	0.33	0.19	0.49	0.08	0.36	0.16	0.49
**Blood parameters**																							
Hematocrit	-0.15	0.01	-0.16	-0.01	-0.15	-0.01	-0.15	-0.02	0.19	-0.16	-0.14	-0.03	-0.11	-0.02	0.06	-0.14	-0.02	-0.14	-0.02	-0.15	0.01	-0.16	-0.01
Hemoglobin	0.02	-0.01	0.01	0.01	0.02	0.01	0.01	0.01	-0.04	-0.01	0.03	0.02	0.00	0.01	-0.01	0.02	0.01	0.00	0.01	0.02	-0.01	0.01	0.01
Sodium	-0.10	0.13	-0.06	0.36	-0.09	0.09	-0.05	0.33	0.34	-0.06	-0.08	0.05	-0.02	0.30	0.46	-0.09	0.08	-0.04	0.32	-0.10	0.13	-0.06	0.36
Potassium	-0.14	-0.07	-0.15	-0.05	-0.15	-0.08	-0.15	-0.06	0.07	-0.12	-0.15	-0.09	-0.15	-0.06	0.01	-0.15	-0.09	-0.15	-0.06	-0.14	-0.07	-0.15	-0.05
Glucose	0.01	-0.05	0.03	0.01	0.01	-0.04	0.03	0.01	-0.10	0.05	0.01	-0.03	0.03	0.01	-0.02	0.01	-0.04	0.03	0.01	0.01	-0.05	0.03	0.01
Total protein	-0.10	-0.18	-0.15	-0.05	-0.11	-0.19	-0.17	-0.07	-0.11	-0.06	-0.14	-0.20	-0.20	-0.10	0.01	-0.12	-0.20	-0.18	-0.08	-0.10	-0.18	-0.15	-0.05
Urea	0.16	0.07	0.14	0.06	0.14	0.06	0.12	0.05	-0.04	0.18	0.12	0.03	0.08	0.03	0.00	0.13	0.05	0.11	0.05	0.16	0.07	0.14	0.06
Blood viscosity	-0.16	0.02	-0.17	0.00	-0.15	-0.01	-0.15	0.00	0.21	-0.17	-0.14	-0.03	-0.12	-0.01	0.08	-0.15	-0.02	-0.14	-0.01	-0.16	0.02	-0.17	0.00
Arterial oxygen content	0.02	-0.03	0.01	0.00	0.02	-0.02	0.01	0.00	-0.07	0.02	0.01	-0.02	0.00	0.00	-0.01	0.02	-0.02	0.00	0.00	0.02	-0.03	0.01	0.00
Venous oxygen content	0.06	-0.05	0.08	0.05	0.08	-0.01	0.08	0.06	-0.16	0.02	0.09	0.02	0.07	0.07	0.01	0.08	0.00	0.08	0.07	0.06	-0.05	0.08	0.05
Arterial oxygen saturation	-0.02	-0.10	-0.01	-0.06	-0.05	-0.13	-0.02	-0.09	-0.08	0.08	-0.08	-0.16	-0.02	-0.12	-0.04	-0.06	-0.14	-0.02	-0.10	-0.02	-0.10	-0.01	-0.06
Oxygen capacity of hemoglobin	0.03	-0.01	0.04	0.06	0.03	-0.01	0.04	0.06	-0.07	0.05	0.02	-0.01	0.03	0.05	0.03	0.03	-0.01	0.04	0.05	0.03	-0.01	0.04	0.06

Parameters	A150	A150 A5	A150 B5	A150 H12.5	A300	A300 A5	A300 B5	A300 H12.5	A5	B5	E20	E20 A5	E20 B5	E20 H12.5	H12.5	L100	L100 A5	L100 B5	L100 H12.5	L50	L50 A5	L50 B5	L50 H12.5
**Renin–angiotensin–aldosterone system**																							
Plasma renin activity	0.71	0.46	0.58	0.14	0.68	0.46	0.53	0.16	-0.13	0.61	0.61	0.40	0.45	0.17	-0.21	0.66	0.44	0.50	0.17	0.72	0.46	0.58	0.14
Plasma renin concentration	0.12	0.05	0.06	-0.09	0.11	0.05	0.06	-0.08	-0.08	0.08	0.09	0.04	0.04	-0.06	-0.18	0.10	0.05	0.05	-0.07	0.12	0.05	0.06	-0.09
Plasma angiotensin I concentration	0.71	0.46	0.58	0.14	0.68	0.46	0.53	0.16	-0.13	0.61	0.61	0.40	0.45	0.17	-0.21	0.66	0.44	0.50	0.17	0.72	0.46	0.58	0.14
Plasma angiotensin II concentration	0.71	0.46	0.58	0.14	0.68	0.46	0.53	0.16	-0.13	0.61	0.61	0.40	0.45	0.17	-0.21	0.66	0.44	0.50	0.17	0.72	0.46	0.58	0.14
Plasma aldosterone concentration	0.02	0.13	0.01	0.16	0.03	0.12	0.02	0.16	0.17	-0.01	0.04	0.11	0.02	0.16	0.17	0.03	0.11	0.02	0.16	0.02	0.13	0.01	0.16
**Hormones**																							
Plasma antidiuretic hormone	0.00	0.21	0.04	0.51	-0.01	0.15	0.04	0.47	0.37	0.07	-0.01	0.09	0.04	0.41	0.58	-0.01	0.13	0.04	0.45	0.00	0.21	0.04	0.51
Plasma atrial natriuretic peptide	-0.02	0.01	0.02	-0.05	0.00	0.02	0.04	-0.04	0.01	-0.05	0.02	0.05	0.07	-0.02	-0.04	0.01	0.03	0.05	-0.03	-0.02	0.00	0.02	-0.05

Table S11. Correlation coefficients of virtual population parameters with reduction of mean arterial pressure in simulated treatment with aliskiren 150 mg (A150) and 300 mg (A300), amlodipine 5 mg (A5), bisoprolol 5 mg (B5), enalapril 20 mg (E20), HCTZ 12.5 mg (H12.5), losartan 100 mg (L100) and 50 mg (L50), and combinations of these drugs.

Parameters	A150	A150 A5	A150 B5	A150 H12.5	A300	A300 A5	A300 B5	A300 H12.5	A5	B5	E20	E20 A5	E20 B5	E20 H12.5	H12.5	L100	L100 A5	L100 B5	L100 H12.5	L50	L50 A5	L50 B5	L50 H12.5
**General characteristics**																							
Body mass index	-0.14	-0.11	-0.16	-0.01	-0.15	-0.13	-0.16	-0.03	0.00	-0.14	-0.15	-0.15	-0.16	-0.05	0.03	-0.15	-0.14	-0.16	-0.04	-0.14	-0.11	-0.16	-0.01
Total body water	-0.04	-0.06	-0.04	-0.09	-0.04	-0.06	-0.04	-0.09	-0.01	-0.04	-0.05	-0.06	-0.04	-0.09	-0.06	-0.04	-0.06	-0.04	-0.09	-0.04	-0.06	-0.04	-0.09
Sodium intake	-0.08	-0.02	-0.09	0.21	-0.08	-0.05	-0.10	0.19	0.10	-0.08	-0.08	-0.08	-0.11	0.16	0.25	-0.08	-0.06	-0.10	0.18	-0.08	-0.02	-0.09	0.21
Water intake	0.09	0.16	0.08	0.04	0.09	0.15	0.07	0.04	0.14	0.09	0.08	0.14	0.07	0.05	0.02	0.09	0.15	0.07	0.04	0.09	0.16	0.08	0.04
Total exchangeable sodium	-0.03	0.00	-0.04	0.01	-0.04	-0.02	-0.04	0.00	0.06	-0.03	-0.04	-0.03	-0.04	0.00	0.03	-0.04	-0.02	-0.04	0.00	-0.03	0.00	-0.04	0.01
Body oxygen demand	-0.08	-0.01	-0.12	-0.01	-0.09	-0.04	-0.12	-0.02	0.10	-0.10	-0.10	-0.08	-0.13	-0.04	0.02	-0.10	-0.06	-0.13	-0.03	-0.08	-0.01	-0.12	-0.01
Total metabolic intensity	0.16	0.17	0.19	0.09	0.17	0.19	0.19	0.10	-0.01	0.18	0.17	0.21	0.20	0.13	0.03	0.17	0.20	0.19	0.12	0.16	0.17	0.19	0.09
**Systemic hemodynamics**																							
Systolic blood pressure	0.17	0.27	0.06	0.16	0.14	0.21	0.04	0.15	0.38	0.14	0.10	0.13	0.02	0.12	0.18	0.13	0.18	0.04	0.14	0.17	0.27	0.06	0.16
Diastolic blood pressure	-0.01	0.02	-0.18	0.04	-0.06	-0.08	-0.20	0.00	0.34	-0.06	-0.12	-0.17	-0.23	-0.06	0.15	-0.08	-0.12	-0.22	-0.02	-0.01	0.02	-0.18	0.05
Heart rate	-0.02	-0.10	-0.01	-0.02	-0.02	-0.09	-0.01	-0.02	-0.09	-0.01	-0.02	-0.07	-0.01	-0.02	0.00	-0.02	-0.08	-0.01	-0.02	-0.02	-0.10	-0.01	-0.02
Systemic arterial tone	0.14	0.22	0.16	0.13	0.15	0.23	0.16	0.15	0.13	0.15	0.15	0.22	0.16	0.17	0.09	0.15	0.22	0.16	0.15	0.14	0.22	0.16	0.13
Sympathetic sensitivity of microvessels	0.00	-0.07	0.00	0.07	-0.01	-0.07	-0.01	0.06	-0.05	0.02	-0.01	-0.07	-0.01	0.05	0.09	-0.01	-0.07	-0.01	0.06	0.00	-0.07	0.00	0.07
Basic systemic arterial elasticity	0.11	0.14	0.14	0.06	0.12	0.16	0.14	0.08	0.02	0.12	0.13	0.17	0.14	0.10	0.01	0.12	0.16	0.14	0.08	0.11	0.14	0.14	0.06
Basic systemic venous elasticity	0.05	0.07	0.05	0.17	0.05	0.07	0.04	0.17	0.06	0.05	0.05	0.06	0.04	0.17	0.16	0.05	0.07	0.04	0.17	0.05	0.07	0.05	0.17
Systemic vascular resistance	0.12	0.10	0.09	0.03	0.12	0.10	0.09	0.04	0.04	0.11	0.11	0.10	0.07	0.05	0.02	0.11	0.10	0.08	0.04	0.12	0.10	0.09	0.03
**Pulmonary hemodynamics**																							
Systolic blood pressure	0.06	0.02	0.04	-0.08	0.06	0.02	0.04	-0.08	-0.02	0.06	0.05	0.02	0.04	-0.08	-0.10	0.06	0.02	0.04	-0.08	0.06	0.02	0.04	-0.08
Diastolic blood pressure	0.03	0.04	0.02	0.00	0.02	0.03	0.01	0.00	0.04	0.03	0.02	0.02	0.01	0.00	0.00	0.02	0.02	0.01	0.00	0.03	0.04	0.02	0.00
Basic pulmonary arterial elasticity	0.03	-0.06	0.03	-0.10	0.03	-0.04	0.03	-0.10	-0.12	0.03	0.03	-0.02	0.03	-0.09	-0.12	0.03	-0.03	0.03	-0.10	0.03	-0.06	0.03	-0.10
Basic pulmonary venous elasticity	0.10	0.05	0.10	0.08	0.10	0.06	0.10	0.08	-0.01	0.10	0.10	0.07	0.10	0.09	0.07	0.10	0.06	0.10	0.09	0.10	0.05	0.10	0.08
Pulmonary vascular resistance	0.13	0.04	0.11	-0.07	0.13	0.06	0.10	-0.06	-0.07	0.12	0.12	0.07	0.10	-0.04	-0.11	0.13	0.07	0.10	-0.05	0.13	0.04	0.11	-0.07
**Left ventricle (LV)**																							
Stroke volume	-0.09	-0.01	-0.10	-0.03	-0.09	-0.04	-0.10	-0.05	0.12	-0.09	-0.10	-0.07	-0.10	-0.07	0.00	-0.10	-0.05	-0.10	-0.05	-0.09	0.00	-0.10	-0.03
Ejection fraction	0.00	-0.03	0.00	-0.02	0.00	-0.03	-0.01	-0.02	-0.04	0.00	0.00	-0.02	-0.01	-0.02	-0.01	0.00	-0.03	-0.01	-0.02	-0.01	-0.03	0.00	-0.02
Cardiac output	-0.13	-0.12	-0.13	-0.05	-0.13	-0.15	-0.13	-0.07	0.03	-0.12	-0.14	-0.16	-0.13	-0.09	0.01	-0.13	-0.16	-0.13	-0.08	-0.13	-0.12	-0.13	-0.05
LV peak systolic pressure	0.15	0.22	0.04	0.14	0.12	0.16	0.02	0.12	0.35	0.12	0.08	0.09	0.00	0.10	0.17	0.10	0.13	0.02	0.11	0.15	0.23	0.04	0.14
LV end-diastolic pressure	-0.01	0.02	0.01	0.02	0.00	0.02	0.02	0.03	0.00	0.00	0.01	0.02	0.02	0.03	0.02	0.00	0.02	0.02	0.03	-0.01	0.02	0.01	0.02
LV end-systolic volume	-0.03	0.03	-0.03	0.00	-0.03	0.01	-0.03	-0.01	0.08	-0.03	-0.03	0.00	-0.03	-0.01	0.01	-0.03	0.01	-0.03	-0.01	-0.03	0.03	-0.03	0.00
LV end-diastolic volume	-0.07	0.02	-0.08	-0.02	-0.07	-0.01	-0.08	-0.03	0.13	-0.07	-0.08	-0.04	-0.08	-0.04	0.01	-0.08	-0.02	-0.08	-0.03	-0.07	0.02	-0.08	-0.02
LV wall elasticity	0.00	-0.02	0.02	-0.02	0.01	-0.01	0.02	-0.02	-0.07	0.01	0.01	0.01	0.02	-0.01	-0.03	0.01	0.00	0.02	-0.01	0.00	-0.02	0.02	-0.02
LV inotropic state	-0.03	-0.05	0.00	0.04	-0.02	-0.04	0.00	0.04	-0.09	-0.02	-0.01	-0.02	0.00	0.05	0.03	-0.02	-0.03	0.00	0.04	-0.03	-0.05	0.00	0.04
**Right ventricle (RV)**																							
RV peak systolic pressure	0.06	0.02	0.03	-0.08	0.05	0.02	0.03	-0.08	0.00	0.05	0.04	0.01	0.03	-0.07	-0.09	0.05	0.01	0.03	-0.08	0.06	0.02	0.03	-0.08
RV end-diastolic pressure	0.03	0.05	0.02	-0.04	0.03	0.05	0.02	-0.04	0.01	0.03	0.03	0.05	0.02	-0.03	-0.06	0.03	0.05	0.02	-0.03	0.03	0.05	0.02	-0.04
RV end-systolic volume	-0.02	0.00	-0.01	0.05	-0.02	0.00	0.00	0.04	0.01	-0.02	-0.02	0.00	0.00	0.04	0.05	-0.02	0.00	0.00	0.04	-0.02	0.00	-0.01	0.05
RV end-diastolic volume	-0.06	-0.01	-0.05	0.02	-0.06	-0.02	-0.05	0.02	0.07	-0.05	-0.06	-0.04	-0.05	0.01	0.04	-0.06	-0.03	-0.05	0.01	-0.06	0.00	-0.05	0.02
RV wall elasticity	0.01	-0.01	0.02	-0.07	0.01	0.00	0.02	-0.07	-0.05	0.01	0.02	0.02	0.02	-0.06	-0.08	0.01	0.01	0.02	-0.07	0.01	-0.01	0.02	-0.07
RV inotropic state	-0.01	0.06	-0.02	-0.04	-0.01	0.05	-0.02	-0.04	0.07	-0.01	-0.01	0.04	-0.02	-0.03	-0.05	-0.01	0.05	-0.02	-0.04	-0.01	0.06	-0.02	-0.04

Parameters	A150	A150 A5	A150 B5	A150 H12.5	A300	A300 A5	A300 B5	A300 H12.5	A5	B5	E20	E20 A5	E20 B5	E20 H12.5	H12.5	L100	L100 A5	L100 B5	L100 H12.5	L50	L50 A5	L50 B5	L50 H12.5
**Peak flow rates through heart valves**																							
Aortic valve peak flow	0.03	0.09	0.05	0.04	0.03	0.09	0.05	0.04	0.06	0.04	0.04	0.08	0.05	0.05	0.02	0.03	0.08	0.05	0.05	0.03	0.09	0.05	0.04
Pulmonary valve peak flow	-0.04	-0.07	-0.05	-0.12	-0.04	-0.08	-0.05	-0.13	-0.03	-0.04	-0.05	-0.08	-0.05	-0.14	-0.11	-0.05	-0.08	-0.05	-0.13	-0.04	-0.07	-0.05	-0.12
LV early peak filling rate	-0.01	-0.03	0.02	-0.02	0.00	-0.02	0.03	-0.02	-0.07	0.01	0.01	0.00	0.03	-0.02	-0.02	0.00	-0.01	0.03	-0.02	-0.01	-0.04	0.02	-0.02
LV active peak filling rate	-0.11	-0.03	-0.19	0.02	-0.14	-0.09	-0.20	-0.01	0.22	-0.13	-0.17	-0.16	-0.22	-0.05	0.10	-0.15	-0.12	-0.21	-0.02	-0.11	-0.02	-0.19	0.02
RV early peak filling rate	-0.01	-0.01	0.00	-0.03	-0.01	0.00	0.00	-0.03	-0.02	-0.01	-0.01	0.00	0.00	-0.03	-0.03	-0.01	0.00	0.00	-0.03	-0.01	-0.01	0.00	-0.03
RV active peak filling rate	-0.10	0.01	-0.20	0.02	-0.13	-0.07	-0.21	-0.02	0.26	-0.13	-0.16	-0.14	-0.23	-0.06	0.09	-0.14	-0.10	-0.22	-0.03	-0.10	0.01	-0.20	0.02
**Renal hemodynamics**																							
Glomerular filtration rate	0.32	0.59	0.29	0.08	0.32	0.56	0.28	0.11	0.49	0.31	0.31	0.49	0.27	0.14	0.00	0.31	0.53	0.28	0.12	0.32	0.60	0.29	0.08
Renal blood flow	0.43	0.25	0.40	0.03	0.42	0.30	0.40	0.06	-0.07	0.42	0.42	0.34	0.40	0.11	-0.06	0.42	0.32	0.40	0.08	0.43	0.24	0.40	0.03
Filtration fraction	0.01	0.35	-0.02	0.09	0.00	0.28	-0.03	0.09	0.48	0.00	-0.01	0.20	-0.04	0.09	0.08	0.00	0.25	-0.03	0.09	0.01	0.36	-0.02	0.09
Filtration coefficient	-0.54	-0.07	-0.64	0.02	-0.57	-0.21	-0.64	-0.03	0.44	-0.58	-0.60	-0.36	-0.64	-0.11	0.12	-0.59	-0.27	-0.64	-0.06	-0.54	-0.06	-0.64	0.02
Renal vascular resistance	-0.40	-0.20	-0.43	0.02	-0.41	-0.28	-0.44	-0.03	0.22	-0.41	-0.42	-0.36	-0.45	-0.09	0.14	-0.41	-0.31	-0.44	-0.05	-0.39	-0.19	-0.43	0.02
Number of nephrons	0.08	0.08	0.02	0.02	0.07	0.06	0.01	0.02	0.05	0.06	0.05	0.04	0.01	0.02	-0.01	0.06	0.05	0.01	0.02	0.08	0.08	0.02	0.02
Afferent arteriole diameter	0.49	0.39	0.59	0.36	0.52	0.48	0.60	0.42	-0.11	0.54	0.56	0.55	0.61	0.49	0.21	0.54	0.51	0.60	0.45	0.49	0.38	0.59	0.36
Efferent arteriole diameter	0.17	0.19	0.16	0.07	0.16	0.20	0.16	0.09	0.04	0.16	0.16	0.21	0.16	0.11	0.01	0.16	0.21	0.16	0.10	0.17	0.19	0.16	0.07
Afferent arteriole length	-0.01	0.01	-0.03	0.04	-0.02	-0.01	-0.03	0.03	0.05	-0.02	-0.02	-0.02	-0.03	0.02	0.04	-0.02	-0.01	-0.03	0.03	-0.01	0.01	-0.03	0.04
Efferent arteriole length	-0.06	-0.05	-0.06	0.01	-0.06	-0.06	-0.06	0.01	0.02	-0.06	-0.06	-0.07	-0.06	-0.01	0.03	-0.06	-0.07	-0.06	0.00	-0.06	-0.05	-0.06	0.01
Afferent arteriolar resistance	-0.45	-0.17	-0.52	0.03	-0.47	-0.28	-0.53	-0.02	0.31	-0.47	-0.49	-0.39	-0.54	-0.10	0.16	-0.48	-0.33	-0.53	-0.05	-0.45	-0.17	-0.52	0.03
Efferent arteriolar resistance	-0.24	-0.23	-0.18	-0.07	-0.23	-0.24	-0.18	-0.09	-0.02	-0.22	-0.21	-0.24	-0.18	-0.12	0.01	-0.22	-0.24	-0.18	-0.10	-0.24	-0.23	-0.18	-0.07
Renal venous resistance	0.12	0.18	0.08	0.15	0.11	0.16	0.07	0.16	0.11	0.11	0.10	0.14	0.06	0.16	0.10	0.10	0.16	0.07	0.16	0.12	0.18	0.08	0.15
Renal venous pressure	0.00	0.07	-0.03	-0.01	-0.01	0.05	-0.03	-0.01	0.09	-0.01	-0.02	0.03	-0.04	-0.02	-0.01	-0.01	0.04	-0.04	-0.01	0.00	0.07	-0.03	-0.01
Glomerular hydrostatic pressure	0.50	0.16	0.52	0.02	0.51	0.23	0.51	0.05	-0.18	0.51	0.51	0.31	0.51	0.10	-0.04	0.51	0.27	0.51	0.07	0.50	0.15	0.52	0.02
Glomerular capillary oncotic pressure	-0.22	-0.22	-0.28	-0.04	-0.24	-0.27	-0.29	-0.07	0.01	-0.24	-0.26	-0.32	-0.29	-0.13	0.04	-0.24	-0.29	-0.29	-0.09	-0.22	-0.22	-0.28	-0.04
Bowman's space hydrostatic pressure	-0.11	-0.13	-0.15	0.07	-0.12	-0.15	-0.15	0.05	-0.03	-0.12	-0.13	-0.17	-0.15	0.02	0.11	-0.13	-0.16	-0.15	0.04	-0.11	-0.13	-0.15	0.07
Macula densa sodium flow rate	0.10	0.48	0.09	0.79	0.10	0.40	0.08	0.77	0.60	0.12	0.09	0.29	0.06	0.74	0.79	0.10	0.36	0.07	0.76	0.10	0.48	0.09	0.79
Fractional proximal sodium reabsorption	0.05	-0.24	0.04	-0.75	0.05	-0.17	0.05	-0.73	-0.41	0.02	0.04	-0.09	0.05	-0.68	-0.79	0.04	-0.14	0.05	-0.71	0.05	-0.24	0.04	-0.75
Fractional distal sodium reabsorption	0.20	0.58	0.19	0.63	0.20	0.52	0.19	0.63	0.60	0.22	0.19	0.42	0.19	0.62	0.59	0.19	0.48	0.19	0.63	0.20	0.59	0.19	0.63
**Blood parameters**																							
Hematocrit	-0.04	0.05	-0.07	0.07	-0.05	0.02	-0.07	0.06	0.13	-0.06	-0.06	-0.02	-0.07	0.04	0.08	-0.06	0.00	-0.07	0.05	-0.04	0.05	-0.07	0.07
Hemoglobin	-0.06	-0.06	-0.05	-0.08	-0.05	-0.06	-0.05	-0.08	-0.03	-0.05	-0.05	-0.06	-0.04	-0.08	-0.06	-0.05	-0.06	-0.04	-0.08	-0.05	-0.06	-0.05	-0.08
Sodium	0.03	0.31	0.03	0.54	0.03	0.25	0.02	0.53	0.42	0.04	0.03	0.17	0.02	0.51	0.55	0.03	0.22	0.02	0.52	0.03	0.31	0.03	0.55
Potassium	-0.14	-0.04	-0.14	0.01	-0.15	-0.07	-0.14	0.00	0.09	-0.15	-0.15	-0.10	-0.14	-0.02	0.04	-0.15	-0.08	-0.14	-0.01	-0.14	-0.04	-0.14	0.01
Glucose	0.06	0.05	0.09	0.01	0.07	0.07	0.09	0.02	-0.05	0.07	0.08	0.09	0.09	0.04	-0.03	0.07	0.08	0.09	0.03	0.06	0.05	0.09	0.01
Total protein	-0.20	-0.30	-0.24	-0.07	-0.21	-0.33	-0.25	-0.09	-0.13	-0.21	-0.23	-0.35	-0.25	-0.14	0.01	-0.22	-0.34	-0.25	-0.11	-0.20	-0.30	-0.24	-0.06
Urea	-0.02	-0.03	-0.02	-0.01	-0.02	-0.03	-0.02	-0.01	0.01	-0.01	-0.02	-0.04	-0.03	-0.02	0.00	-0.02	-0.04	-0.02	-0.02	-0.02	-0.03	-0.02	-0.01
Blood viscosity	-0.05	0.06	-0.08	0.08	-0.06	0.02	-0.08	0.07	0.15	-0.06	-0.07	-0.02	-0.08	0.05	0.09	-0.06	0.00	-0.08	0.06	-0.05	0.06	-0.08	0.08
Arterial oxygen content	-0.06	-0.06	-0.04	-0.06	-0.05	-0.06	-0.04	-0.06	-0.03	-0.05	-0.05	-0.06	-0.03	-0.06	-0.04	-0.05	-0.06	-0.04	-0.06	-0.06	-0.06	-0.04	-0.06
Venous oxygen content	-0.02	-0.08	0.01	-0.06	-0.01	-0.06	0.02	-0.05	-0.09	0.00	0.00	-0.04	0.03	-0.05	-0.05	-0.01	-0.05	0.02	-0.05	-0.02	-0.08	0.01	-0.06
Arterial oxygen saturation	-0.05	-0.08	-0.05	0.01	-0.05	-0.08	-0.05	0.00	-0.04	-0.05	-0.05	-0.08	-0.04	-0.01	0.03	-0.05	-0.08	-0.04	0.00	-0.05	-0.08	-0.05	0.01
Oxygen capacity of hemoglobin	0.06	0.09	0.10	0.11	0.07	0.11	0.10	0.12	0.01	0.08	0.08	0.12	0.10	0.13	0.07	0.08	0.11	0.10	0.12	0.06	0.09	0.10	0.11

Parameters	A150	A150 A5	A150 B5	A150 H12.5	A300	A300 A5	A300 B5	A300 H12.5	A5	B5	E20	E20 A5	E20 B5	E20 H12.5	H12.5	L100	L100 A5	L100 B5	L100 H12.5	L50	L50 A5	L50 B5	L50 H12.5
**Renin–angiotensin–aldosterone system**																							
Plasma renin activity	0.39	0.18	0.35	-0.07	0.39	0.23	0.34	-0.03	-0.13	0.38	0.38	0.27	0.32	0.02	-0.16	0.38	0.25	0.34	-0.02	0.39	0.18	0.36	-0.07
Plasma renin concentration	0.00	-0.09	0.01	-0.14	0.01	-0.07	0.01	-0.14	-0.14	0.00	0.01	-0.05	0.00	-0.13	-0.16	0.01	-0.06	0.01	-0.14	0.00	-0.09	0.01	-0.15
Plasma angiotensin I concentration	0.39	0.18	0.35	-0.07	0.39	0.23	0.34	-0.03	-0.13	0.38	0.38	0.27	0.32	0.02	-0.16	0.38	0.25	0.34	-0.02	0.39	0.18	0.36	-0.07
Plasma angiotensin II concentration	0.39	0.18	0.35	-0.07	0.39	0.23	0.34	-0.03	-0.13	0.38	0.38	0.27	0.32	0.02	-0.16	0.38	0.25	0.34	-0.02	0.39	0.18	0.36	-0.07
Plasma aldosterone concentration	0.02	0.14	0.01	0.15	0.02	0.12	0.01	0.15	0.18	0.02	0.02	0.09	0.01	0.14	0.14	0.02	0.11	0.01	0.15	0.02	0.15	0.01	0.15
**Hormones**																							
Plasma antidiuretic hormone	0.04	0.39	0.05	0.70	0.05	0.32	0.04	0.68	0.53	0.07	0.05	0.22	0.03	0.65	0.71	0.05	0.28	0.04	0.67	0.04	0.40	0.05	0.70
Plasma atrial natriuretic peptide	0.09	0.04	0.09	-0.02	0.09	0.06	0.10	-0.01	-0.04	0.09	0.09	0.08	0.10	0.00	-0.05	0.09	0.07	0.10	-0.01	0.09	0.04	0.09	-0.02

References

Abate G, Zito M, Carbonin PU, Cocchi A, Cucinotta D, Manopulo R, Senin U, Aisa G, Savastano V, Rodegher P (1998) Pinacidil and hydrochlorothiazide alone or in combination in the treatment of hypertension in the elderly. Curr Ther Res Clin Exp 59(1):62–71.

Agabiti Rosei E, Morelli P, Rizzoni D (2005) Effects of nifedipine GITS 20 mg or enalapril 20 mg on blood pressure and inflammatory markers in patients with mild-moderate hypertension. Blood Press Suppl 1:14–22.

Ajayi AA, Oyewo EA, Ladipo GO, Akinsola A (1989) Enalapril and hydrochlorothiazide in hypertensive Africans. European Journal of Clinical Pharmacology 36:229–234.

Asmar RG, Kerihuel JC, Girerd XJ, Safar ME (1991) Effect of bisoprolol on blood pressure and arterial hemodynamics in systemic hypertension. American journal of cardiology 68:61–64.

Bakris GL, Hart P, Ritz E (2006) Beta blockers in the management of chronic kidney disease. Kidney International 70:1905–1913.

Bauer JH (1984) Role of angiotensin converting enzyme inhibitors in essential and renal hypertension. Effects of captopril and enalapril on renin-angiotensin-aldosterone, renal function and hemodynamics, salt and water excretion, and body fluid composition. The American Journal of Medicine 77(2A):43–51.

Bauer JH, Jones LB (1984) Comparative studies: enalapril versus hydrochlorothiazide as first-step therapy for the treatment of primary hypertension. American Journal of Kidney Diseases 4(1):55–62.

Blumenfeld JD, Sealey JE, Mann SJ, Bragat A, Marion R, Pecker MS, Sotelo J, August P, Pickering TG, Laragh JH (1999) Beta-adrenergic receptor blockade as a therapeutic approach for suppressing the renin-angiotensin-aldosterone system in normotensive and hypertensive subjects. American Journal of Hypertension 12(5):451–459.

Bokuda K, Morimoto S, Seki Y, Yatabe M, Watanabe D, Yatabe J, Ando T, Shimizu S, Itoh H, Ichihara A (2018) Greater reductions in plasma aldosterone with aliskiren in hypertensive patients with higher soluble (Pro)renin receptor level. Hypertension Research 41(6):435–443.

Brown MJ, McInnes GT, Papst CC, Zhang J, MacDonald TM (2011) Aliskiren and the calcium channel blocker amlodipine combination as an initial treatment strategy for hypertension control (ACCELERATE): a randomised, parallel-group trial. Lancet 377(9762):312–320.

Buter H, Navis G, Dullaart RP, de Zeeuw D, de Jong PE (2001) Time course of the antiproteinuric and renal haemodynamic responses to losartan in microalbuminuric IDDM. Nephrology Dialysis Transplantation 16:771–775.

Cherney DZ, Scholey JW, Jiang S, Har R, Lai V, Sochett EB, Reich HN (2012) The effect of direct renin inhibition alone and in combination with ACE inhibition on endothelial function, arterial stiffness, and renal function in type 1 diabetes. Diabetes Care 35(11):2324–2330.

Corea L, Cardoni O, Fogari R, Innocenti P, Porcellati C, Provvidenza M, Meilenbrock S, Sullivan J, Bodin F (1996) Valsartan, a new angiotensin II antagonist for the treatment of essential hypertension: a comparative study of the efficacy and safety against amlodipine. Clin Pharmacol Ther 60(3):341–346.

Cushman WC, Duprez DA, Weintraub HS, Purkayastha D, Zappe D, Samuel R, Izzo JL Jr (2012) Home and clinic blood pressure responses in elderly individuals with systolic hypertension. J Am Soc Hypertens 6(3):210–218.

De Rosa ML, Cardace P, Rossi M, Baiano A, de Cristofaro A (2002) Comparative effects of chronic ACE inhibition and AT1 receptor blocked losartan on cardiac hypertrophy and renal function in hypertensive patients. Journal of Human Hypertension 16:133–140.

Delles C, Klingbeil AU, Schneider MP, Handrock R, Weidinger G, Schmieder RE (2003) Direct comparison of the effects of valsartan and amlodipine on renal hemodynamics in human essential hypertension. American Journal of Hypertension 16:1030–1035.

Derosa G, Bonaventura A, Romano D, Bianchi L, Fogari E, D'Angelo A, Maffioli P (2014) Effects of enalapril/lercanidipine combination on some emerging biomarkers in cardiovascular risk stratification in hypertensive patients. J Clin Pharm Ther 39(3):277–285.

Devineni D, Vaccaro N, Polidori D, Rusch S, Wajs E (2014) Effects of hydrochlorothiazide on the pharmacokinetics, pharmacodynamics, and tolerability of canagliflozin, a sodium glucose co-transporter 2 inhibitor, in healthy participants. Clinical Therapeutics 36(5):698–710.

Dickstein K, Chang P, Willenheimer R, Haunsø S, Remes J, Hall C, Kjekshus J (1995) Comparison of the effects of losartan and enalapril on clinical status and exercise performance in patients with moderate or severe chronic heart failure. Journal of the American College of Cardiology 26(2):438–445.

Digne-Malcolm H, Frise MC, Dorrington KL (2016) How do antihypertensive drugs work? Insights from studies of the renal regulation of arterial blood pressure. Frontiers in Physiology 7:320.

Dobovisek J, Kanic V, Niegowska J, Rekic S, Soucek M, Spinar J (2005) Efficacy and tolerability of losartan in patients with essential hypertension: a multicentre, double-blind comparison of losartan with metoprolol. Journal of Clinical and Basic Cardiology 8(1-4):43–46.

Dussol B, Moussi-Frances J, Morange S, Somma-Delpero C, Mundler O, Berland Y (2005) A randomized trial of furosemide vs hydrochlorothiazide in patients with chronic renal failure and hypertension. Nephrology Dialysis Transplantation 20(2):349–353.

Dussol B, Moussi-Frances J, Morange S, Somma-Delpero C, Mundler O, Berland Y (2012) A pilot study comparing furosemide and hydrochlorothiazide in patients with hypertension and stage 4 or 5 chronic kidney disease. The Journal of Clinical Hypertension 14(1):32–37.

Eguchi K, Hoshide S, Kario K (2015) Effects of celiprolol and bisoprolol on blood pressure, vascular stiffness, and baroreflex sensitivity. American Journal of Hypertension 28(7):858–867.

Fagher B, Henningsen N, Hulthén L, Katzman P, Thulin T (1990) Antihypertensive and renal effects of enalapril and slow-release verapamil in essential hypertension. A double-blind, randomized study. European Journal of Clinical Pharmacology 39(1):S41–S43.

Fermé I, Djian J, Tcherdakoff P (1990) Comparative study on monotherapy with sustained-release diltiazem 300 mg and enalapril 20 mg in mild to moderate arterial hypertension. J Cardiovasc Pharmacol 16 Suppl 1:S46–S50.

Fisher NDL, Danser AHJ, Nussberger J, Dole WP, Hollenberg NK (2008) Renal and hormonal responses to direct renin inhibition with aliskiren in healthy humans. Circulation 117:3199–3205.

Glück Z, Reubi FC (1986) Acute changes in renal function induced by bisoprolol, a new cardioselective beta-blocking agent. European Journal of Clinical Pharmacology 31:107–111.

Goldberg MR, Tanaka W, Barchowsky A, Bradstreet TE, McCrea J, Lo MW, McWilliams EJJr, Bjornsson TD (1993) Effects of losartan on blood pressure, plasma renin activity, and angiotensin II in volunteers. Hypertension 21:704–713.

González-Abraldes J, Albillos A, Bañares R, Del Arbol LR, Moitinho E, Rodríguez C, González M, Escorsell A, García-Pagán JC, Bosch J (2001) Randomized comparison of long-term losartan versus propranolol in lowering portal pressure in cirrhosis. Gastroenterology 121:382–388.

Gottlieb SS, Dickstein K, Fleck E, Kostis J, Levine TB, LeJemtel T, DeKock M (1993) Hemodynamic and neurohormonal effects of the angiotensin II antagonist losartan in patients with congestive heart failure. Circulation 88(part 1):1602–1609.

Gradman AH, Schmieder RE, Lins RL, Nussberger J, Chiang Y, Bedigian MP (2005) Aliskiren, a novel orally effective renin inhibitor, provides dose-dependent antihypertensive efficacy and placebo-like tolerability in hypertensive patients. Circulation 111(8):1012–1018.

Higashi Y, Oshima T, Sasaki S, Nakano Y, Kambe M, Matsuura H, Kajiyama G (1998) Angiotensin-converting enzyme inhibition, but not calcium antagonism, improves a response of the renal vasculature to L-arginine in patients with essential hypertension. Hypertension 32:16–24.

Honore P (1987) Bisoprolol versus hydrochlorothiazide plus amiloride in essential hypertension, a randomized double-blind study. European Heart Journal 8(Suppl. M):95–102.

Houlihan CA, Allen TJ, Baxter AL, Panangiotopoulos S, Casley DJ, Cooper ME, Jerums G (2002) A low-sodium diet potentiates the effects of losartan in type 2 diabetes. Diabetes Care 25:663–671.

Iñigo P, Campistol JM, Lario S, Piera C, Campos B, Bescós M, Oppenheimer F, Rivera F (2001) Effects of losartan and amlodipine on intrarenal hemodynamics and TGF-beta(1) plasma levels in a crossover trial in renal transplant recipients. Journal of the American Society of Nephrology 12:822–827.

Kamishirado H, Inoue T, Fujito T, Kase M, Shimizu M, Sakai Y, Takayanagi K, Morooka S, Natsui S (1997) Effect of enalapril maleate on cerebral blood flow in patients with chronic heart failure. Angiology 48(8):707–713.

Klein IHHT, Ligtenberg G, Oey PL, Koomans HA, Blankestijn PJ (2003) Enalapril and losartan reduce sympathetic hyperactivity in patients with chronic renal failure. Journal of the American Society of Nephrology 14:425–430.

Koh KK, Quon MJ, Han SH, Chung WJ, Ahn JY, Seo YH, Kang MH, Ahn TH, Choi IS, Shin EK (2004) Additive beneficial effects of losartan combined with simvastatin in the treatment of hypercholesterolemic, hypertensive patients. Circulation 110(24):3687–3692.

Konstam MA, Kronenberg MW, Rousseau MF, Udelson JE, Melin J, Stewart D, Dolan N, Edens TR, Ahn S, Kinan D, et al. (1993) Effects of the angiotensin converting enzyme inhibitor enalapril on the long-term progression of left ventricular dilatation in patients with asymptomatic systolic dysfunction. SOLVD (Studies of Left Ventricular Dysfunction) Investigators. Circulation 88(part 1):2277–2283.

Kutumova E, Kiselev I, Sharipov R, Lifshits G, Kolpakov F (2021) Thoroughly Calibrated Modular Agent-Based Model of the Human Cardiovascular and Renal Systems for Blood Pressure Regulation in Health and Disease. Front Physiol 12:746300.

Kwakernaak AJ, Roksnoer LC, Lambers Heerspink HJ, van den Berg-Garrelds I, Lochorn GA, van Embden Andres JH, Klijn MA, Kobori H, Danser AH, Laverman GD, Navis GJ (2017) Effects of direct renin blockade on renal and systemic hemodynamics and on RAAS activity, in weight excess and hypertension: a randomized clinical trial. PLoS One 12(1):e0169258.

Lee SE, Kim YJ, Lee HY, Yang HM, Park CG, Kim JJ, Kim SK, Rhee MY, Oh BH, Investigators (2012) Efficacy and tolerability of fimasartan, a new angiotensin receptor blocker, compared with losartan (50/100 mg): a 12-week, phase III, multicenter, prospective, randomized, double-blind, parallel-group, dose escalation clinical trial with an optional 12-week extension phase in adult Korean patients with mild-to-moderate hypertension. Clin Ther 34(3):552–568, 568.e1–e9.

Leeman M, van de Borne P, Collart F, Vandenhoven G, Peeters L, Mélot C, Degaute JP (1993) Bisoprolol and atenolol in essential hypertension: effects on systemic and renal hemodynamics and on ambulatory blood pressure. Journal of Cardiovascular Pharmacology and Therapeutics 2:785–791.

Leth A (1970) Changes in plasma and extracellular fluid volumes in patients with essential hypertension during long-term treatment with hydrochlorothiazide. Circulation 42:479–485.

Licata G, Scaglione R, Ganguzza A, Parrinello G, Costa R, Merlino G, Corrao S, Amato P (1993) Effects of amlodipine on renal haemodynamics in mild to moderate hypertensive patients. European Journal of Clinical Pharmacology 45:307–311.

Lijnen P, Fagard R, Staessen J, Amery A (1981) Effect of chronic diuretic treatment on the plasma renin-angiotensin-aldosterone system in essential hypertension. British Journal of Clinical Pharmacology 12:387–392.

Lithell H, Selinus I, Hosie J, Frithz G, Weiner L (1987) Efficacy and safety of bisoprolol and atenolol in patients with mild to moderate hypertension: a double-blind, parallel group international multicentre study. Eur Heart J 8 Suppl M:55–64.

Mallion JM (2007) An evaluation of the initial and long-term antihypertensive efficacy of zofenopril compared with enalapril in mild to moderate hypertension. Blood Press Suppl 2:13–18.

Martina B, Dieterle T, Weinbacher M, Battegay E (1999) Effects of losartan titrated to Losartan/Hydrochlorothiazide and amlodipine on left ventricular mass in patients with mild-to-moderate hypertension. A double-blind randomized controlled study. Cardiology 92(2):110–114.

Materson BJ, Reda DJ, Williams DW (1998) Comparison of effects of antihypertensive drugs on heart rate: changes from baseline by baseline group and over time. Department of Veterans Affairs Cooperative Study Group on Antihypertensive Agents. American Journal of Hypertension 11:597–601.

Moore FD (1967) Body composition and its measurement in vivo. British Journal of Surgery 54(13):431–435.

Morales E, Caro J, Gutierrez E, Sevillano A, Auñón P, Fernandez C, Praga M (2015) Diverse diuretics regimens differentially enhance the antialbuminuric effect of renin-angiotensin blockers in patients with chronic kidney disease. Kidney International 88:1434–1441.

Morrone LF, Ramunni A, Fassianos E, Saracino A, Coratelli P, Passavanti G (2003) Nitrendipine and amlodipine mimic the acute effects of enalapril on renal haemodynamics and reduce glomerular hyperfiltration in patients with chronic kidney disease. Journal of Human Hypertension 17:487–493.

Mountokalakis TD (1997) The renal consequences of arterial hypertension. Kidney International 51:1639–1653.

Nadler SB, Hidalgo JU, Bloch T (1962) Prediction of blood volume in normal human adults. Surgery 51:224–232.

Narkiewicz K (2007) Comparison of home and office blood pressure in hypertensive patients treated with zofenopril or losartan. Blood Press Suppl 2:7–12.

Natarajan AR, Eisner GM, Armando I, Browning S, Pezzullo JC, Rhee L, Dajani M, Carey RM, Jose PA (2016) The renin-angiotensin and renal dopaminergic systems interact in normotensive humans. Journal of the American Society of Nephrology 27:265–279.

Nedogoda SV, Ledyaeva AA, Chumachok EV, Tsoma VV, Mazina G, Salasyuk AS, Barykina IN (2013) Randomized trial of perindopril, enalapril, losartan and telmisartan in overweight or obese patients with hypertension. Clin Drug Investig 33(8):553–561.

Nussberger J, Brunner DB, Waeber B, Brunner HR (1986) Specific measurement of angiotensin metabolites and in vitro generated angiotensin II in plasma. Hypertension 8:476–482.

Nussberger J, Brunner D, Keller I, Brunner HR (1992) Measurement of converting enzyme activity by antibody-trapping of generatedangiotensin II. Comparison with two other methods. American journal of hypertension 5:393–398.

Nussberger J, Wuerzner G, Jensen C, Brunner HR (2002) Angiotensin II suppression in humans by the orally active renin inhibitor Aliskiren (SPP100): comparison with enalapril. Hypertension 39(1):E1–E8.

Ogawa S, Nako K, Okamura M, Senda M, Mori T, Ito S (2011) Aliskiren reduces albuminuria and oxidative stress, and elevates glomerular filtration rates in Japanese patients with advanced diabetic nephropathy. Hypertension Research 34:400–401.

Ohashi H, Oda H, Ohno M, Watanabe S, Itou H, Araki H, Yokoyama H, Sakata S (2002) Losartan reduces proteinuria and preserves renal function in hypertensive patients with IgA nephropathy. Clinical and Experimental Nephrology 6:224–228.

Okada Y, Shibata S, Fujimoto N, Best SA, Levine BD, Fu Q (2017) Long-term effects of a renin inhibitor versus a thiazide diuretic on arterial stiffness and left ventricular diastolic function in elderly hypertensive patients. American Journal of Physiology-Regulatory, Integrative and Comparative Physiology 313:R400–R409.

Oparil S, Barr E, Elkins M, Liss C, Vrecenak A, Edelman J (1996) Efficacy, tolerability, and effects on quality of life of losartan, alone or with hydrochlorothiazide, versus amlodipine, alone or with hydrochlorothiazide, in patients with essential hypertension. Clin Ther 18(4):608–625.

Oparil S, Abate N, Chen E, Creager MA, Galet V, Jia G, Julius S, Lerman A, Lyle PA, Pool J, Tershakovec AM (2008) A double-blind, randomized study evaluating losartan potassium monotherapy or in combination with hydrochlorothiazide versus placebo in obese patients with hypertension. Curr Med Res Opin 24(4):1101–1114.

Ott C, Schneider MP, Raff U, Ritt M, Striepe K, Alberici M, Schmieder RE (2012) Effects of manidipine vs. amlodipine on intrarenal haemodynamics in patients with arterial hypertension. British Journal of Clinical Pharmacology 75(1):129–135.

Ozeki A, Amiya E, Watanabe M, Hosoya Y, Takata M, Watanabe A, Kawarasaki S, Nakao T, Watanabe S, Omori K, Yamada N, Tahara Y, Hirata Y, Nagai R (2014) Effect of add-on aliskiren to type 1 angiotensin receptor blocker therapy on endothelial function and autonomic nervous system in hypertensive patients with ischemic heart disease. J Clin Hypertens (Greenwich) 16(8):591–598.

Pareek AK, Messerli FH, Chandurkar NB, Dharmadhikari SK, Godbole AV, Kshirsagar PP, Agarwal MA, Sharma KH, Mathur SL, Kumbla MM (2016) Efficacy of Low-Dose Chlorthalidone and Hydrochlorothiazide as Assessed by 24-h Ambulatory Blood Pressure Monitoring. J Am Coll Cardiol 67(4):379–389.

Parrinello G, Paterna S, Torres D, Di Pasquale P, Mezzero M, La Rocca G, Cardillo M, Trapanese C, Caradonna M, Licata G (2009) One-year renal and cardiac effects of bisoprolol versus losartan in recently diagnosed hypertensive patients: a randomized, double-blind study. Clinical Drug Investigation 29(9):591–600.

Paterna S, Parrinello G, Scaglione R, Costa R, Bova A, Palumbo VA, Pinto A, Amato P, Licata G (2000) Effect of long-term losartan administration on renal haemodynamics and function in hypertensive patients. Cardiovascular Drugs and Therapy 14:529–532.

Paterna S, Parrinello G, Di Pasquale P, Torres D, La Rocca G, Antona R, Vernuccio L, Fornaciari E, Tarantino A, Piccione E, Fasullo S, Licata G (2007) Medium-term effects of bisoprolol administration on renal hemodynamics and function in mild to moderate essential hypertension. Advances in Therapy 24(6):1260–1270.

Persson F, Rossing P, Reinhard H, Juhl T, Stehouwer CDA, Schalkwijk C, Danser AHJ, Boomsma F, Frandsen E, Parving H-H (2009) Renal effects of aliskiren compared with and in combination with irbesartan in patients with type 2 diabetes, hypertension, and albuminuria. Diabetes Care 32(10):1873–1879.

Pool JL, Schmieder RE, Azizi M, Aldigier JC, Januszewicz A, Zidek W, Chiang Y, Satlin A (2007) Aliskiren, an orally effective renin inhibitor, provides antihypertensive efficacy alone and in combination with valsartan. Am J Hypertens 20(1):11–20.

Porthan K, Viitasalo M, Hiltunen TP, Vaananen H, Dabek J, Suonsyrja T, Hannila-Handelberg T, Virolainen J, Nieminen MS, Toivonen L, Kontula K, Oikarinen L (2009) Short-term electrophysiological effects of losartan, bisoprolol, amlodipine, and hydrochlorothiazide in hypertensive men. Ann Med 41(1):29–37.

Puig JG, Calvo C, Luurila O, Luurila H, Sulosaari S, Strandberg A, Ghezzi C (2007) Lercanidipine, enalapril and their combination in the treatment of elderly hypertensive patients: placebo-controlled, randomized, crossover study with four ABPM. J Hum Hypertens 21(12):917–924.

Reams GP, Lau A, Hamory A, Bauer JH (1987) Amlodipine therapy corrects renal abnormalities encountered in the hypertensive state. American Journal of Kidney Diseases 10(6):446–451.

Roman MJ, Alderman MH, Pickering TG, Pini R, Keating JO, Sealey JE, Devereux RB (1998) Differential effects of angiotensin converting enzyme inhibition and diuretic therapy on reductions in ambulatory blood pressure, left ventricular mass, and vascular hypertrophy. American Journal of Hypertension 11:387–396.

Satomura A, Fujita T, Fuke Y, Wada Y, Matsumoto K (2009) Change of glomerular hemodynamics in patients with advanced chronic kidney disease after cilnidipine therapy. The Open Clinical Chemistry Journal 2:31–36.

Scaglione R, Indovina A, Parrinello G, Lipari R, Mulè LG, Ganguzza A, Capuana G, Stampino CG, Licata G (1992) Antihypertensive efficacy and effects of nitrendipine on cardiac and renal hemodynamics in mild to moderate hypertensive patients: randomized controlled trial versus hydrochlorothiazide. Cardiovascular Drugs and Therapy 6:141–146.

Scaglione R, Ganguzza A, Corrao S, Costa R,釘Paternà S, Cannavo MG, Parrinello G, Di Chiara T, D'Aubert MD, Cottone C, et al. (1995) Effects of cilazapril on renal haemodynamics and function in hypertensive patients: a randomised controlled study versus hydrochlorothiazide. Blood Pressure 4:363–368.

Schmitt F, Natov S, Martinez F, Lacour B, Hannedouche TP (1996) Renal effects of angiotensin I-receptor blockade and angiotensin convertase inhibition in man. Clinical Science 90: 205–213.

Schroten NF, Damman K, Hemmelder MH, Voors AA, Navis G, Gaillard CAJM, van Veldhuisen DJ, Van Gilst WH, Hillege HL (2015) Effect of additive renin inhibition with aliskiren on renal blood flow in patients with Chronic Heart Failure and Renal Dysfunction (Additive Renin Inhibition with Aliskiren on renal blood flow and Neurohormonal Activation in patients with Chronic Heart Failure and Renal Dysfunction). American Heart Journal 169(5):693–701.e3.

Semplicini A, Rossi GP, Bongiovì S, Perissinotto F, Samà B, Mozzato MG, Pessina AC (1986) Time course of changes in blood pressure, aldosterone and body fluids during enalapril treatment: a double-blind randomized study vs hydrochlorothiazide plus propranolol in essential hypertension. Clinical and Experimental Pharmacology and Physiology 13:17–24.

Siddiqi L, Oey PL, Blankestijn PJ (2011) Aliskiren reduces sympathetic nerve activity and blood pressure in chronic kidney disease patients. Nephrology Dialysis Transplantation 26:2930–2934.

Simon G, Morioka S, Snyder DK, Cohn JN (1983) Increased renal plasma flow in long-term enalapril treatment of hypertension. Clinical Pharmacology and Therapeutics 34(4):459–465.

Stanton A, Jensen C, Nussberger J, O'Brien E (2003) Blood pressure lowering in essential hypertension with an oral renin inhibitor, aliskiren. Hypertension 42:1137–1143.

Sun F, Song Y, Liu J, Ma LJ, Shen Y, Huang J, Zhou YL (2016) Efficacy of losartan for improving insulin resistance and vascular remodeling in hemodialysis patients. Hemodial Int 20(1):22–30.

Suojanen L, Haring A, Tikkakoski A, Koskela JK, Tahvanainen AM, Huhtala H, Kähönen M, Sipilä K, Eräranta A, Mustonen JT, Kivistö K, Pörsti IH (2017) Haemodynamic influences of bisoprolol in hypertensive middle-aged men: a double-blind, randomized, placebo-controlled cross-over study. Basic and Clinical Pharmacology and Toxicology 121:130–137.

Tham TC, Herity N, Guy S, Silke B (1993) Haemodynamic comparison of amlodipine and atenolol in essential hypertension using the quantascope. British Journal of Clinical Pharmacology 36:555–560.

Toupance O, Lavaud S, Canivet E, Bernaud C, Hotton JM, Chanard J (1994) Antihypertensive effect of amlodipine and lack of interference with cyclosporine metabolism in renal transplant recipients. Hypertension 24:297–300.

van Brummelen P, Woerlee M, Schalekamp MA (1979) Long-term versus short-term effects of hydrochlorothiazide on renal haemodynamics in essential hypertension. Clinical Science 56:463–469.

van Brummelen P, Man in't Veld AJ, Schalekamp MA (1980) Hemodynamic changes during long-term thiazide treatment of essential hypertension in responders and nonresponders. Clinical Pharmacology and Therapeutics 27(3):328–336.

van Campen JSJA, de Boer K, van de Veerdonk MC, van der Bruggen CEE, Allaart CP, Raijmakers PG, Heymans MW, Marcus JT, Harms HJ, Handoko ML, de Man FS, Noordegraaf AV, Bogaard H-J (2016) Bisoprolol in idiopathic pulmonary arterial hypertension: an explorative study. European Respiratory Journal 48(3):787–796.

Venkat-Raman G, Feehally J, Elliott HL, Griffin P, Moore RJ, Olubodun JO, Wilkinson R (1998) Renal and haemodynamic effects of amlodipine and nifedipine in hypertensive renal transplant recipients. Nephrology Dialysis Transplantation 13:2612–2616.

Venkat Raman G, Feehally J, Coates RA, Elliott HL, Griffin PJ, Olubodun JO, Wilkinson R (1999) Renal effects of amlodipine in normotensive renal transplant recipients. Nephrology Dialysis Transplantation 14:384–388.

Villamil A, Chrysant SG, Calhoun D, Schober B, Hsu H, Matrisciano-Dimichino L, Zhang J (2007) Renin inhibition with aliskiren provides additive antihypertensive efficacy when used in combination with hydrochlorothiazide. Journal of Hypertension 25:217–226.

Weber MA, Byyny RL, Pratt JH, Faison EP, Snavely DB, Goldberg AI, Nelson EB (1995) Blood pressure effects of the angiotensin II receptor blocker, losartan. Arch Intern Med 155(4):405–411.

Wiggins KJ, Kelly DJ (2009) Aliskiren: a novel renoprotective agent or simply an alternative to ACE inhibitors? Kidney Int 76(1):23–31.

Williams B, MacDonald TM, Morant S, Webb DJ, Sever P, McInnes G, Ford I, Cruickshank JK, Caulfield MJ, Salsbury J, Mackenzie I, Padmanabhan S, Brown MJ, British Hypertension Society's PATHWAY Studies Group (2015) Spironolactone versus placebo, bisoprolol, and doxazosin to determine the optimal treatment for drug-resistant hypertension (PATHWAY-2): a randomised, double-blind, crossover trial. Lancet 386(10008):2059–2068.

Wing LMH, Arnolda LF, Upton J, Molloy D (2003) Candesartan and hydrochlorothiazide in isolated systolic hypertension. Blood Press 12(4):246–254.
